# Domain-specific participant recruitment exceeds the application of “Targeted” advertisement from common online advertising platforms

**DOI:** 10.1017/cts.2025.61

**Published:** 2025-04-10

**Authors:** Joseph Powell, Kyle Webster, Siobhan Efionayi, Timothy Engelman, W.H. Wilson Tang, P. Xiao Li

**Affiliations:** 1 Department of Biochemistry, Case Western Reserve University, Cleveland, OH, USA; 2 Center for RNA Science and Therapeutics, Case Western Reserve University, Cleveland, OH, USA; 3 Department of Computer and Data Sciences, Case Western Reserve University, Cleveland, OH, USA; 4 Department of Cardiovascular and Metabolic Sciences, Lerner Research Institute, Cleveland Clinic, Cleveland, OH, USA; 5 Department of Cardiovascular Medicine, Heart Vascular and Thoracic Institute, Cleveland Clinic, Cleveland, OH, USA

**Keywords:** Study design, recruitment, implementation, diversity, engagement, retention

## Abstract

**Introduction:**

Insufficient sample sizes threatened the fidelity of the primary research trials. Even if the research group recruits a sufficient sample size, the sample may lack diversity, reducing the generalizability of the results of the study. Evaluating the effectiveness of online advertising platforms (e.g., Facebook & Google Ads) versus traditional recruitment methods (e.g., flyers, clinical participation) is essential.

**Methods:**

Patients were recruited through email, electronic direct message, paper advertisements, and word-of-mouth advertisement (traditional) or through Google Ads and Facebook Ads (advertising) for a longitudinal study on monitoring COVID-19 using wearable devices. Participants were asked to wear a smart watch-like wearable device for ∼ 24 hours per day and complete daily surveys.

**Results:**

The initiation conversion rate (ICR, impressions to pre-screen ratio) was better for traditional recruitment (24.14) than for Google Ads, 28.47 ([0.80, 0.88]; p << 0.001). The consent conversion rate (CCR, impressions to consent ratio) was also higher for traditional recruitment (66.54) than for Google Ads, 2961.20 ([0.015, 0.030]; p << 0.001). Participants recruited through recommendations or by paper flier were more likely to participate initially (Χ^2^ = 23.65; p < 0.005). Clinical recruitment led to more self-reporting white participants, while other methods yielded great diversity (Χ^2^ = 231.47; p << 0.001).

**Conclusions:**

While Google Ads target users based on keywords, they do not necessarily improve participation. However, our findings are based on a single study with specific recruitment strategies and participant demographics. Further research is needed to assess the generalizability of these findings across different study designs and populations.

## Introduction

Insufficient subject recruitment threatens clinical research success, with a substantial proportion of trials failing to meet their initial recruitment targets, thereby compromising the reliability and generalizability of study findings [1,2]. Inadequate sample sizes undermine statistical analyses and jeopardize the validity of primary research questions, forcing researchers to consider extending recruitment timelines, revising study objectives, or terminating trials prematurely [3–6]. However, such decisions entail ethical dilemmas and resource implications, further complicating the research process [7].

While traditional recruitment methods, such as advertisements and word-of-mouth, remain effective, they can be costly and prone to bias, limiting their utility in reaching diverse populations [8–11]. Consequently, researchers are increasingly exploring online recruitment strategies, including patient portals and paid advertisements, which offer advantages like broader reach but also present challenges such as cost-effectiveness and demographic biases [12–17]. Balancing these recruitment approaches is crucial for ensuring the success and integrity of clinical research endeavors while striving for inclusivity and representation across diverse populations.

We initially recruited participants using electronic mail, electronic direct messaging, paper advertisements, word-of-mouth advertising, Google Ads, Facebook advertisements, and electronic mail via Electronic Medical Records (EMR) software. We analyzed data from our recruitment campaign to observe various recruitment methods’ relative efficiency and cost-effectiveness. We hypothesized that we would reach more people through electronic means, specifically Google Ads, potentially creating access to additional population groups that are not usually available by traditional means.

## Methods

### Recruitment

This study was approved by the Case Western Reserve University IRB (IRB Protocol Numbers: STUDY20191761; STUDY20200415) and the Cleveland Clinic IRB (IRB 20-872). Participants were initially recruited to participate as part of a study investigating the use of wearable device technology in detection and prediction of COVID-19 and other common infectious diseases.

**Figure 1.**
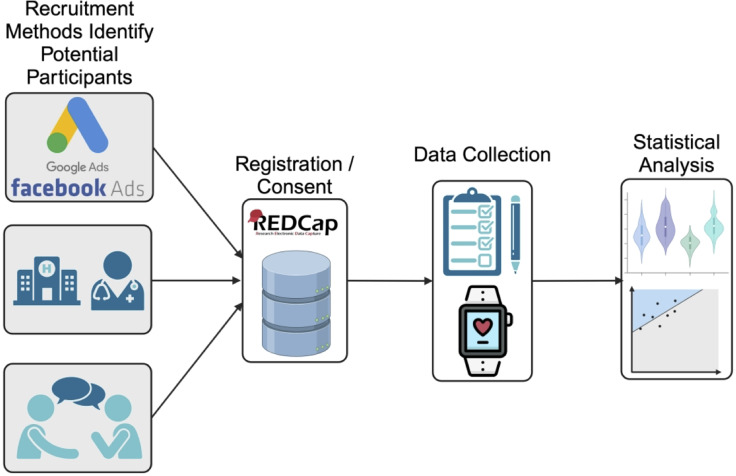
Brief overview of the study protocol. Participants were recruited from one of the following sources (top to bottom) advertising platforms (e.g. Google Ads), clinical sources, and from recommendations or active recruitment (i.e. participants reaching out to the study team about participating in the study). Registration and consent were completed on a HIPAA compliant REDCap server. Daily survey and wearable data were collected on participants who completed consent. Statistical analysis was then performed on both demographics, survey, and wearable data.

Participants were encouraged to wear the watch for up to 2 years for approximately 24 hours per day, but were explicitly told that they can remove the watch as needed for comfort and charging. Participants were also encouraged to fill out a daily survey regarding their general health, taking on average 1 minute to complete with exceptions for missing days. The presence of data from both the wearable device and the survey were recorded and used to evaluate the participants adherence at 1 year following consent. A proportion of available data was calculated as the number of observations / the number of expected observations for survey data (Figure 1).

### Traditional recruitment

Traditional recruitment methods included electronic mail, electronic direct message via Cleveland Clinic’s MyChart system, paper advertisements, and word-of-mouth advertisement. Electronic mail was sent out using CWRU available mailing lists. MyChart messages were sent out in batches to patients registered in the Cleveland Clinic COVID-19 registry, which includes all patients tested for COVID-19 at a Cleveland Clinic site. Paper advertisements were placed in high-traffic areas in both CWRU, such as student commons, and in clinics so as to be easily visible by patients.

Following an impression, i.e. the potential participant seeing or hearing about the study from one of the above methods, participants voluntarily navigated through the provided hyperlink or QR code to the study’s REDCap website, starting with a prescreening questionnaire. Participants completed a prescreening questionnaire to establish eligibility for the study, followed by the informed consent and participant demographics form. The method of recruitment was collected in the prescreening questionnaire. The window of analysis for traditional recruitment was be from 1January 2020 through 30 June 2023.

### Advertising recruitment

Following initial recruitment, a Google Ads account was created and activated. The study team worked with a Google Ads account specialist to curate a list of keywords based upon the language used in our “traditional recruitment” materials and the specialist’s expertise. When potential participants searched for one of these terms, our advertisement had a chance to be shown based upon the Google Ads proprietary preference algorithm. Participants were directed through the advertisement hyperlink to our study page on the lab’s website as desired by Google. Participants then navigated to the study REDcap page. Participants completed the remaining study prescreening and consent process as described above to maintain consistency with the rest of the study. A tiered approach was adopted to evaluate maximization of budget and visibility.

Ad performance was based upon Google Analytics keywords and impression numbers (Supp. Table 1). Keywords were varied based on the ad’s performance. Keywords were added based on similarity to other high-performance keywords. Likewise, keywords were removed if they showed low overall performance, i.e. low impression and/or the price associated with search importance (bidding values). We worked with our assigned Google Ads team member to identify potential keywords that were similar to our traditional recruitment methods and were most likely to be used by patient’s search for studies or information that could lead to studies similar to this study. Over 400 keyword changes were made for the ad during the duration of the ad and are shown in Supp. Table 1. Ads were shown in the following states (abbreviated and in no particular order): WV, DC, VA, TX, TN, SC, PA, OK, OH, NC, NY, MO, MI, MS, MD, LA, KY, IN, IL, GA, FL, DE, AR, and AK. The Google campaign ran from 8 November 2021 to 30 May 2022.

Similarly, a Facebook ad account was also created; however, we decided to approach Facebook advertising in a more cost-effective manner. We identified various COVID-related Facebook groups, and participants of these groups were invited to join the study. Relevant groups were identified by keyword searches related to the study performed by the study team. Advertisements were posted in these groups with the permission of the group moderators. Ad activity was monitored, and the ad was reposted in the Facebook group when ad traffic began to decrease.

### Statistical analysis

To evaluate the success in reaching potential participants through traditional recruitment or advertising recruitment (Google Ads / Facebook Ads), we investigated the conversion rate of each method. We evaluated conversion percentage as the number of consented individuals/the number of impressions, via bootstrap t-test analysis.

We compared demographic group distributions of consented individuals to evaluate if there were any enriched populations from any recruitment method. Categorical variables were evaluated using the Fisher’s Exact test for proportions, and continuous variables were evaluated using nonparametric Kruskal–Wallis Test. The Fisher’s Exact test is used as an alternative for the Chi-Square test of proportions due to the expected frequency of one or more of the cells less than 5.

All statistical tests were performed using R Statistical Software version 4.1.2 (R Core Team).

## Results

We evaluated each group of recruitment methods for enriched demographic characteristics. There was no statistically significant difference in any recruitment method when comparing whether a participant has a chronic comorbidity or continued participation in the study 1-year after consent (*p >* 0.05). There are no significant differences in the gender distribution when a participant is recommended to join our study (Χ^2^ = 31.88; *p* = 0.0602). There are statistically significant differences in the participation of the study between recruitment methods, which suggest that participants who actively seek out the study (i.e. participants who found the study through recommendations or by paper flier) are more likely to participate initially (Χ^2^ = 23.65; *p <* 0.005). There is also a statistically significant difference in the self-reported ethnicity distribution of participants (Χ^2^ = 231.47; *p <*< 0.001), age (*p* < 0.01), and proportion of surveys completion (*p* < 0.005). We found that through recruitment via the Cleveland Clinic’s MyChart system, there are significantly more people self-reporting as white, and there are more diverse participants that consent to the study from other recruitment methods (Table 1).

**Table 1.** Demographic overview of participants from the three main recruitment groups (left to right) traditional (clinical and active) and advertising

	Traditional		Online Ad
	Clinical	Active	Google/Facebook
	(n = 1397)	(n = 14)	(n = 15)
Sex/gender			
Male	399 (28.6%)	2 (14.35)	5 (33.3%)
Female	994 (71.2%)	12 (85.7%)	10 (66.7%)
Other	1 (0.1%)	0 (0%)	0 (0%)
Prefer not to say	3 (0.2%)	0 (0%)	0 (0%)
Age (years)			
Mean (SD)	49.6 (14.9)	45.6 (16.6)	41.2 (15.0)
Median [Min, Max]	49.0 [18.0, 92.0]	49.0 [18.0, 72.0]	39.0 [18.0, 78.0]
Ethnicity			
American Indian or Alaskan Native	5 (0.4%)	0 (0%)	0 (0%)
Asian	16 (1.1%)	1 (7.1%)	1 (6.7%)
Black or African America	145 (10.4%)	1 (7.1%)	3 (20.0%)
Native Hawaiian or Pacific Islander	1 (0.1%)	0 (0%)	0 (0%)
White	1118 (80.0%)	11 (78.6%)	7 (46.7%)
Hispanic or Latino or of Spanish Origin	61 (4.4%)	1 (7.1%)	3 (20.0%)
Not Hispanic or Latino or of Spaish Origin	24 (1.7%)	0 (0%)	0 (0%)
Other	13 (0.9%)	0 (0%)	0 (0%)
Prefer not to say	14 (1.0%)	0 (0%)	1 (6.7%)
Has a chronic condition			
No	970 (69.4%)	11 (78.6%)	10 (66.7%)
Yes	427 (30.6%)	3 (21.4%)	5 (33.3%)
Submitted wearable data			
Yes	277 (19.8%)	7 (50.0%)	4 (26.7%)
No	1120 (80.2%)	7 (50.0%)	11 (73.3%)
Submitted survey data			
Yes	337 (24.1%)	6 (42.9%)	0 (0%)
No	1060 (75.9%)	8 (57.1%)	15 (100%)

**Figure 2.**
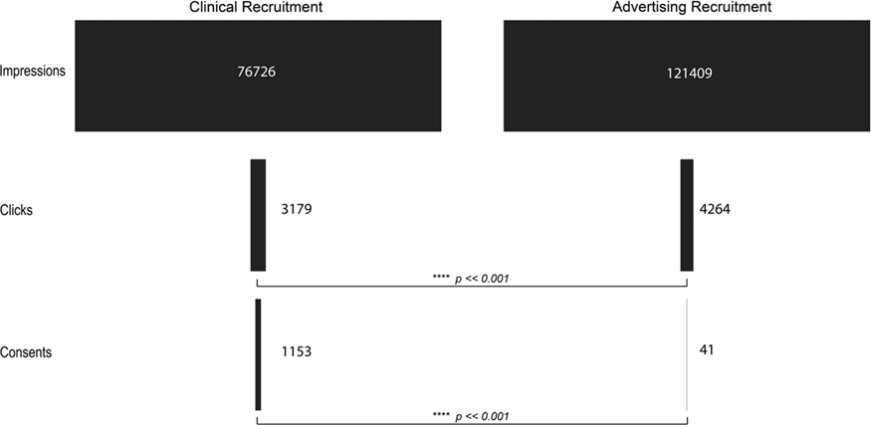
Visualization of the flow of participants from the impression or initiation of recruitment to completion of consent for clinical recruitment (left) and advertising recruitment (right). *p-values* indicate the result of a bootstrap *t*-test analysis.

**Figure 3.**
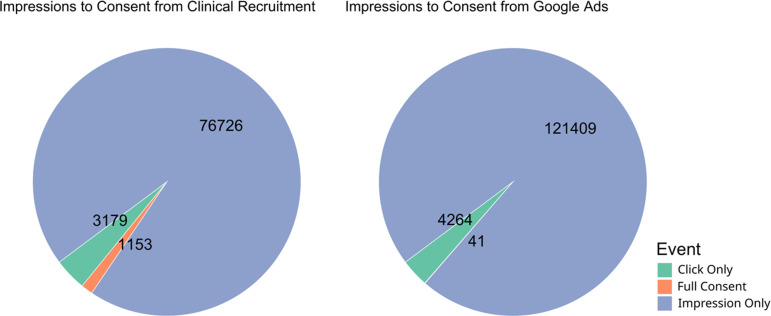
Pie chart of proportion participants belonging to each part of the recruitment process from impression to consent. Numbers represent the total number of participants that reached each stage, while color and size of the pie chart represent the proportion of total participants.

We evaluated the viability of using Google Ads, due to the ease of tracking contact and progression through this method, for recruitment in a scientific study where the primary purpose was not related to the advertisements themselves. To accomplish this, we analyzed the conversion rate of each of the methods used for recruitment. The initiation conversion rate (ICR, impressions to pre-screen ratio) for clinical recruitment was better, 24.14, compared to the ICR for Google Ads, 28.47 ( [0.80, 0.88]; *p <*< 0.001). The consent conversion rate (CCR, impressions to consent ratio) for clinical recruitment was also better, 66.54, compared to the CCR for Google Ads, 2961.20 ( [0.015, 0.030]; *p <*< 0.001) (Table 2; Figures 2 and 3). Evaluation of other traditional recruitment methods such as flyers and personal recommendations were not evaluated here due to the difficulty of accurately evaluating the impression column of the traditional recruitment. Flyers were placed in high-traffic areas and recommendations can occur at unknown frequencies based on participant enthusiasm.

**Table 2.** The flow of potential participants from the impression to consent for clinical recruitment (top) and advertising (bottom). Absolute numbers (left) indicate that advertising platforms are better at reaching a larger audience in a shorter time frame. However, when comparing the ratio of impressions (right) to either click or consent, advertising platforms are significantly worse at converting an impression to a consent

Event	Number of Participants	Impression to event ratio
*Clinical*		
Impression	76,726	1
Click	3179	24.14
Consent	1153	66.54
*Advertising*		
Impression	121409	1
Click	4264	28.47****
Consent	41	2961.2****

**** *p* << 0.001.

## Discussion

There are multiple resources available for researchers to use for the recruitment of patients, such as flyers, clinical collaborations, and targeted advertising, but it’s still unclear which method is the best for producing adherent and unbiased sample populations. We attempted to answer this by comparing traditional recruitment methods, e.g. flyers and clinical invitations, and online advertising, e.g. Google Ads. We found that the ICR (impressions to prescreen ratio) for traditional recruitment was better, 24.14, compared to the ICR for Google Ads, 28.47 ( [0.80, 0.88]; *p <*< 0.001). The CCR (impressions to consent ratio) for traditional recruitment was also better, 66.54, compared to the CCR for Google Ads, 2961 ( [0.015, 0.030]; *p <*< 0.001). In addition, we found that participants who actively seek out the study (i.e. participants who found the study through recommendations or by paper flier) are more likely to participate initially (Χ^2^ = 23.65; *p <* 0.005), and there are significantly more people self-reporting as white when recruited clinically, while there are more diverse participants that consent to the study from other recruitment methods (Χ^2^ = 231.47; *p <*< 0.001).

The difference in the ICR and CCR between clinical recruitment and advertising recruitment indicates that for getting a participant to initiate the prescreening, the difference between traditional and Google recruitment is small but still meaningful. However, there is a large difference in the completion of the informed consent between the two groups. This indicates that, while Google Ads are targeted based upon keywords, while the desire to participate in medical studies may be high from patients searching for medically related research, the financial burden on the researchers may stifle the ability of these patients to find research studies. In our study, for example, we spent $USD 20,032.16 over the course of our advertising campaign, which without a large amount of funding could prove intractable for many research groups. In contrast, we spent approximately $USD 20.00 on our recruitment through traditional means.

For our Facebook advertising, in order to keep our costs to a minimum, we decided against a formal Facebook advertising campaign. Instead, we identified several Facebook groups that represented our desired patient population. We then proceed to ask for permission to recruit directly from these groups by making advertising posts in the group message boards. Notably, the selection of groups we identified and reached out to may introduce bias, as it was based on our knowledge of existing communities and their visibility. Additionally, group moderators’ decisions on whether to allow recruitment posts may reflect their own goals for the community, further influencing participant selection. Furthermore, patients who find these studies in medically related situations, e.g. through a clinic or in medically related areas like a medical school, are more likely to find the research that they are interested in participating in. Additionally, there may also be an increase in patient trust when research is presented to them from a medical source. All potential participants regardless of recruitment method eventually landed at our REDcap study site. Our collaboration with the Google Ads team required us to link the Ad to our lab website before the participant could proceed to the REDcap site to complete the informed consent and demographics surveys. Participants recruited from traditional methods and Facebook were not required to go to our lab site prior to visiting the REDcap site, which potentially added another barrier to entry for participants.

While our research suggests that traditional methods were more effective than online advertising. Recent literature has suggested the opposite [15, 18–21]. Interestingly, in all the contravening studies Facebook was the most effective online recruitment tool [15, 20, 21, 22]. As an example, Watson et al. [15 ] recruited 49.3% and 3.84% of their participants using Facebook and Google Ads, respectively. On the other hand, Sato et al. [23 ] found web advertisements less successful than the other used modes of recruitment. Additionally, Topolovec-Vranic et al. [24 ] found Google Ads similarly ineffective compared to other recruitment methods. As such, our results might reflect our preference for Google Ads instead of Facebook.

Looking at the population’s diversity, we found that participants recruited clinically were significantly more likely to self-report as white. In contrast, other recruitment methods drew a more diverse population. These results track with the literature. As the results from Table 2 show, clinical recruitment, especially EMR-based recruitment, can be highly efficient. They can also be cost-effective and fast. However, clinical recruitment is limited to the patients in a given clinic’s healthcare system, some of which service populations that are more or less diverse [25]. However, a review of the Cleveland Clinic Foundation patient population’s demographics falls outside this article’s scope. With that said, EMR-based recruiting has underrepresented minority populations [26–28]. Aware of this, research groups often employ supplemental recruitment methods, targeting underrepresented populations [16]. Online recruitment methods enable research groups access to individuals of different ethnicities, socioeconomic backgrounds, and populations that are notoriously difficult to reach with traditional recruitment methods [16,29,31,32]. Looking at our results, we are encouraged that the nonclinical recruitment methods produced a more diverse population.

### Limitations

We recognize that there are some limitations to the analysis for this study at this time, and in the future, we hope to address these concerns. Firstly, we cannot rule out the impact of the COVID-19 pandemic on our recruitment methods. Our traditional recruitment occurred primarily during the beginning of the pandemic, while the advertising recruitment occurred primarily during the second and third years of the pandemic. Secondly, we note that recruitment time frames differ by the nature of the recruitment method. For traditional recruitment methods such as flyers and word-of-mouth advertising, the timeframe is indefinite. Whereas, for advertising recruitment, the campaigns lasted 6 months (Nov 2021–May 2022). Due to resource and study design constraints, we are unable to fully resolve this issue. However, we conducted an additional analysis by restricting the timeframe for traditional recruitment to align more closely with advertising recruitment. Encouragingly, the findings on impressions remain consistent. Specifically, the clinical ICR is 26.0 and the clinical CCR is 73.8, both of which are significantly different from the advertising ICR and CCR. Thirdly, we recognize that for the online advertising recruitment, we are in part reliant on the Google Ads team for help in keyword design. This Google Ads employee may be biased without their knowledge, which could lead to differences in how participants see the advertisement. Fourthly, we are unable to identify the exact position, or the relative ranking of our ads compared to others as Google and Facebook do not provide this information. Knowledge of the relative positioning of our advertisements compared to others would allow us to potentially normalize our results for better insights.

In the future, we hope to create a more balanced dataset that can provide a more robust statistical understanding of the population we are working with. Continued cooperation with our collaborators, as well as improvements to our documentation infrastructure of the study, can help to improve our identification of potential participants and our documentation of where those participants are coming from.

Funding: This project was supported by the Clinical and Translational Science Collaborative of Northern Ohio which is funded by the National Institutes of Health, National Center for Advancing Translational Sciences, Clinical and Translational Science Award grant, UM1TR004528. The content is solely the responsibility of the authors and does not necessarily represent the official views of the NIH. The study was also funded by NIH R01 Grants 1R01HL159170 and 1R01NR02010501.

Disclosure: Dr Tang has served as consultant for Sequana Medical, Cardiol Therapeutics, Genomics plc, Zehna Therapeutics, WhiteSwell, Boston Scientific, CardiaTec Biosciences, Bristol Myers Squibb, Alleviant Medical, Alexion Pharmaceuticals, Salubris Biotherapeutics, BioCardia, and has received honorarium from Springer, Belvoir Media Group, and American Board of Internal Medicine.

## Supporting information

Powell et al. supplementary material Powell et al. supplementary material

## Supplementary material

The supplementary material for this article can be found at https://doi.org/10.1017/cts.2025.61.
