# Supplementary material for: Domain-specific participant recruitment exceeds the application of “Targeted” advertisement from common online advertising platforms
Source: J Clin Transl Sci. 2025 Apr 10;9(1):e106. doi: 10.1017/cts.2025.61 (PMC12209964; doi:10.1017/cts.2025.61)
Supplement: Powell et al. supplementary material [file S2059866125000615sup001.pdf]

Search term	Match type
**vaccination medical appointment scheduling software**	Broad match
**hipaa scheduling software**	Broad match
**healthstream learning center**	Broad match
**masters in health informatics and information management**	Broad match
**archiving medical records**	Broad match
**johns hopkins lockdown research**	Broad match
**registered health information technician**	Broad match
**personal health record online**	Broad match
**personal medical records**	Broad match
**health commerce system new york state**	Broad match
**health records management ehr**	Exact match
**assessing health literacy in patients**	Broad match
**care coordination platforms**	Broad match
**phreesia healthcare**	Broad match
**where to get a titer test**	Phrase match (close variant)
**value based care readiness assessment**	Broad match
**caretracker patient portal**	Broad match
**what are electronic health records ehrs emrs**	Exact match
**iqvia studies**	Broad match
**touting virus cure**	Broad match
**physician billing software**	Phrase match (close variant)
**hospital**	Phrase match (close variant)
**hospital report card**	Broad match
**medical history questionnaire**	Broad match
**value based kpis for the healthcare industry**	Broad match
**mit vaccine study**	Broad match
**medicaid disease management programs**	Broad match
**medical device data analytics**	Broad match
**https nextmd enroll now**	Broad match
**patient access training program**	Broad match
**natural immunity israel study**	Broad match

**health studies near me**	Broad match
**emr psychology**	Broad match
**how to get my medical records online**	Phrase match (close variant)
**healthcare workflow process**	Broad match
**healthcare training platform**	Broad match
**nychart**	Broad match
**home health aide training**	Broad match
**what is an emr**	Exact match
**medical device qms**	Broad match
**population health manager job**	Broad match
**sigmacare emr**	Phrase match (close variant)
**easy clinic software**	Broad match
**novavax us approval timeline**	Broad match
**is there a vaccine for pneumonia**	Broad match
**complex case management program description**	Broad match
**klas population health**	Broad match
**pcr test with results in 24 hours near me**	Broad match
**epic clinical applications**	Broad match
**patient admission**	Broad match
**medical assist programs**	Broad match
**new patient registration forms printable**	Broad match
**view my medical records online usa**	Broad match
**meaningful use standards**	Broad match
**health information technology**	Broad match
**patient health records**	Exact match
**duke research studies**	Broad match
**janssen clinical trials**	Broad match
**best hospital management software**	Phrase match (close variant)
**health information management him**	Broad match
**vision emr software**	Broad match
**prognocis**	Broad match
**medical records clerk**	Broad match

**rsv vaccine study**	Broad match
**eclinicalworks 11 training**	Broad match
**emr digital**	Phrase match (close variant)
**nha electronic health records specialist certification exam st**	Phrase match
**how to assess your health**	Broad match
**patient screening questions**	Broad match
**epic medical records training**	Broad match
**vaccine 4th**	Broad match
**how do you check your immune system**	Exact match (close variant)
**health data standards**	Broad match
**asset management hospital**	Broad match
**electronic health record ehr software**	Broad match
**modulemd**	Broad match
**practice code healow**	Broad match
**identify medical office software systems**	Broad match
**free tuberculosis testing near me**	Broad match
**health information and management systems**	Phrase match (close variant)
**where to get a tb test**	Phrase match (close variant)
**how to get into quality assurance in healthcare**	Broad match
**software for healthcare**	Exact match (close variant)
**surgical information systems**	Broad match
**what is a patient health questionnaire**	Broad match
**population health management solutions**	Broad match
**define population health management**	Broad match
**azzley**	Broad match
**maestro software healthcare**	Phrase match (close variant)
**patients records**	Exact match
**enhanced care management providers**	Broad match
**epic charting system for nurses**	Broad match
**hmis health management information system**	Phrase match (close variant)
**guide to clinical documentation**	Broad match
**healthcare medical billing**	Broad match

**wound care software**	Broad match
**masters in population health**	Broad match
**telehealth app for pc**	Broad match
**clinicaltrials gov moderna**	Broad match
**medical manager software demo**	Phrase match (close variant)
**free medical practice**	Broad match
**clinical information systems**	Broad match
**pfizer trial data release**	Broad match
**what is a population health manager**	Broad match
**patient case management software**	Exact match
**healthstream**	Broad match
**healthcare software testing**	Phrase match (close variant)
**health services**	Broad match
**elation emr**	Phrase match (close variant)
**medical records technician**	Phrase match (close variant)
**healthcare consulting companies new york**	Broad match
**nextmd patient portal**	Broad match
**electronic health record system**	Exact match
**flatiron health**	Broad match
**medical records conversion**	Exact match
**molnupiravir human trials**	Broad match
**paid study trials**	Broad match
**mips healthcare**	Broad match
**naat pcr rapid test**	Broad match
**emr vs epic**	Exact match
**emr solution**	Phrase match (close variant)
**health analytics**	Broad match
**healthcare software solutions**	Exact match (close variant)
**medhost training**	Broad match
**medical records management**	Exact match (close variant)
**healthcare academy learning management system**	Broad match
**epic medical billing software training**	Broad match

**mynovant**	Broad match
**patient registration system**	Broad match
**what is a special type of health information**	Broad match
**electronic medical records request**	Exact match
**what is mdvip program**	Broad match
**clinical information systems director**	Broad match
**open source clinical trials**	Broad match
**reporting medical errors**	Broad match
**modmed competitors**	Broad match
**hospital information system providers**	Broad match
**ge healthcare software**	Phrase match (close variant)
**healthcare case studies**	Broad match
**titre test**	Exact match (close variant)
**online diagnostic lab reporting system**	Broad match
**facets healthcare application**	Broad match
**ensemble study clinical trial**	Broad match
**electronic health records policy**	Exact match
**medical billing systems**	Exact match (close variant)
**medical information management**	Broad match
**epic software tutorial**	Exact match
**c 19 study**	Exact match (close variant)
**patient medical records software**	Broad match
**nch mychart account**	Broad match
**psychiatric emr**	Broad match
**clinical studies kansas city**	Broad match
**free patient intake form template**	Broad match
**advancedmd pm**	Broad match
**digital medical records companies**	Exact match
**hospital web application**	Broad match
**patient online pre registration**	Broad match
**online mha program**	Broad match
**autoimmune questionnaire**	Broad match

**vaccination healthcare appointment scheduling service**	Broad match
**electronic health declaration form**	Phrase match
**drug trials**	Broad match
**using epic for research**	Broad match
**medical concierge**	Broad match
**health management platform**	Exact match
**patients form**	Broad match
**homecare software**	Broad match
**what is a health screening questionnaire**	Broad match
**free downloadable medical forms**	Broad match
**emr for psychologists**	Broad match
**custom hospital software development**	Broad match
**how to use medical manager**	Broad match
**healthcare database administrator**	Broad match
**software for medical records**	Phrase match (close variant)
**in healthcare**	Broad match
**where can i go to get a tb test**	Phrase match (close variant)
**clarify health**	Broad match
**health care survey questionnaire**	Broad match
**centricity ge healthcare**	Broad match
**epic medical software**	Broad match
**long term care ehr software**	Broad match
**phytel**	Broad match
**epic electronic health records system**	Exact match
**eclinicalworks login for providers**	Broad match
**helios virtual health**	Broad match
**how to use electronic medical records**	Exact match
**health assessment questionnaire pdf**	Broad match
**medical record laws**	Broad match
**for my health**	Broad match
**phizer vaccine study**	Broad match
**clinical documentation system**	Broad match

**benefits of business intelligence in healthcare**	Broad match
**stellar health**	Broad match
**patient medical record system**	Exact match
**nextgen healthcare practice management software**	Phrase match (close variant)
**cdc digital health**	Phrase match (close variant)
**patient management**	Exact match (close variant)
**northwell edu medical records**	Broad match
**fdoh patient portal org registration**	Broad match
**electronic health records system**	Exact match (close variant)
**aetna population health management**	Broad match
**how to use epic charting**	Broad match
**electronic medical record history**	Exact match
**vision emr**	Phrase match (close variant)
**meditech certification**	Broad match
**smart emr login**	Broad match
**health records technician**	Phrase match (close variant)
**medical records work**	Phrase match (close variant)
**patient registration software**	Exact match (close variant)
**home health charting software**	Broad match
**wound care emr software**	Broad match
**emr analyst**	Broad match
**free hospital management system**	Broad match
**business intelligence in healthcare industry**	Broad match
**epic emr system demo**	Phrase match (close variant)
**what is epic emr system**	Exact match
**patient healthcare monitoring software pricing canada**	Phrase match (close variant)
**healthcare assessment questions**	Broad match
**electronic medical record**	Broad match
**healthcare appointment scheduling software**	Broad match
**physician survey**	Broad match
**best emr for physical therapy**	Phrase match (close variant)
**health learning management system**	Broad match

**practice management software**	Broad match
**electronic health record**	Broad match
**school health management software**	Broad match
**patient safety and risk management software united states**	Broad match
**electronic health record vs electronic medical record**	Exact match
**ehr health records management system**	Exact match
**ob gyn ehr**	Broad match
**care 21 hospital management system**	Phrase match (close variant)
**employee health software for hospitals**	Broad match
**hospital system management**	Broad match
**point click care progress notes**	Broad match
**population health insights**	Broad match
**what is epic healthcare**	Exact match
**benefits of using an electronic health record**	Exact match
**what is emr**	Exact match
**patient electronic health records**	Exact match (close variant)
**novavax vaccine**	Broad match
**home health care time tracking**	Broad match
**medicare beneficiary care management program**	Broad match
**epic healthcare software training**	Broad match
**nephrology software**	Broad match
**hospital management software system**	Exact match (close variant)
**medical records retention by state**	Broad match
**who manages electronic health records**	Exact match
**patient forms pdf**	Broad match
**herpes trials**	Broad match
**healthcare leadership program**	Broad match
**my health rec**	Exact match (close variant)
**medical manager**	Broad match
**nyu my chart login**	Broad match
**paxlovid trial nejm**	Broad match
**fauci admits gain of function research**	Broad match

**what is a disease management program**	Broad match
**epic corporation**	Exact match
**how to use epic healthcare software**	Exact match
**mayo clinic health information**	Phrase match (close variant)
**my healthcare online**	Broad match
**nextgen ehr**	Broad match
**medical health**	Broad match
**top ehr systems 2020**	Phrase match (close variant)
**psychiatric software**	Broad match
**new patient registration software**	Broad match
**information systems health care**	Broad match
**ecw software training**	Broad match
**xsolis healthcare**	Broad match
**medical office software programs**	Broad match
**crimson software healthcare**	Phrase match (close variant)
**kareo practice manager download**	Broad match
**healthcare software vendors**	Broad match
**how to use emr software**	Broad match
**hospital standardization program**	Broad match
**what is epic in healthcare**	Exact match
**virtual health**	Broad match
**healthcare content management system**	Phrase match (close variant)
**scheduling software medical**	Phrase match (close variant)
**epic billing tutorial**	Broad match
**electronic medical records assessment**	Exact match
**how to use epic charting system**	Exact match
**healthcare software development**	Broad match
**meditech training**	Broad match
**remdesivir clinical trial**	Broad match
**electronic health record platforms**	Exact match (close variant)
**medical chart notes**	Broad match
**epic company wisconsin**	Exact match

**long term health management**	Broad match
**epic ehr for small practice**	Phrase match (close variant)
**patient acess**	Broad match
**patient medical report**	Broad match
**my beaumont health chart**	Broad match
**immunization screening questionnaire**	Broad match
**medication management software**	Broad match
**what is emr**	Exact match (close variant)
**best ehr for solo practice**	Phrase match (close variant)
**sigma care emr**	Phrase match (close variant)
**mychart com login**	Broad match
**clinicaltrials**	Broad match
**health fusion emr**	Phrase match (close variant)
**what is epic systems**	Exact match
**patient files**	Broad match
**ehr software**	Phrase match (close variant)
**how to get a medical release form**	Broad match
**epic ehr training video**	Phrase match (close variant)
**patient admission forms**	Broad match
**what are ehr**	Exact match
**how to be a responsible student in this pandemic**	Broad match
**health systems management**	Broad match
**medical records online free**	Broad match
**healthcare portal**	Broad match
**benefits of electronic health records**	Exact match
**cook county vaccine mandate**	Broad match
**population health training**	Broad match
**software physical therapy**	Broad match
**emr record**	Exact match
**electronic health record definition**	Broad match
**electronic personal health record**	Broad match
**population health management case study**	Broad match

**point click care aide charting**	Broad match
**skilled nursing progress note**	Broad match
**case studies in healthcare**	Broad match
**test results and electronic medical records**	Phrase match (close variant)
**electronic health and billing records**	Broad match
**practice management in healthcare**	Broad match
**care assessment need score**	Broad match
**case management software social services**	Broad match
**kareo kareo**	Broad match
**hospital management application**	Exact match (close variant)
**medical record management**	Phrase match
**new patient medical forms**	Phrase match
**idc healthcare management**	Broad match
**wtc health program providers**	Broad match
**virtual care management**	Broad match
**medent software training**	Broad match
**healthcare classes**	Broad match
**medication therapy management programs**	Broad match
**free online ehr training**	Broad match
**ehr for small practice**	Broad match
**medical scheduling**	Broad match
**epic emr demo**	Broad match
**marketplace healthcare contact number**	Broad match
**six components of health information system**	Broad match
**urology ehr**	Broad match
**advance md**	Exact match
**physcian**	Broad match
**patient assessment test**	Broad match
**epic emr systems**	Exact match
**health care tech companies**	Broad match
**health information solutions**	Broad match
**healthcare experience**	Broad match

**access to hospital medical records**	Broad match
**pfizer studies**	Exact match (close variant)
**request of medical records form**	Phrase match (close variant)
**what is digital healthcare**	Broad match
**heath management systems**	Exact match (close variant)
**c19clinicaltrials com**	Phrase match (close variant)
**smart hospital solutions**	Broad match
**crm healthcare**	Broad match
**medtrainer reviews**	Broad match
**john hopkins study**	Phrase match (close variant)
**care pathway software**	Broad match
**meaningful use of electronic health records**	Exact match
**telehealth survey questions**	Broad match
**hospital accounting system**	Broad match
**electronic medical record system**	Broad match
**medical forms**	Broad match
**online medical asst programs**	Broad match
**how to build a telemedicine platform**	Broad match
**medical development**	Broad match
**patient safety certificate program**	Broad match
**nextmd com**	Broad match
**my health patient portal**	Broad match
**business analyst in healthcare domain**	Broad match
**occupational therapy software**	Broad match
**titers and immunity testing**	Phrase match (close variant)
**free medical records software**	Broad match
**patient records**	Exact match (close variant)
**epic documentation training**	Broad match
**patient billing**	Broad match
**health catalyst**	Broad match
**crm for healthcare**	Phrase match (close variant)
**online healthcare degree programs**	Broad match

**livehealth**	Broad match
**mychart login**	Broad match
**c19 study website**	Phrase match (close variant)
**health information website**	Phrase match (close variant)
**medical referral system**	Broad match
**patient manager software**	Exact match (close variant)
**electronic documentation in nursing**	Broad match
**healthcare contract management**	Broad match
**moderna study**	Exact match (close variant)
**epic laboratory information system**	Broad match
**pfizer clinical trials**	Exact match (close variant)
**lab results online**	Broad match
**clinical management program**	Broad match
**point care manager**	Broad match
**medical record certification**	Broad match
**agile in healthcare**	Broad match
**medical records 2020**	Broad match
**epic health software**	Exact match
**titer test**	Exact match (close variant)
**epic electronic health record software**	Broad match
**beaumont health mychart**	Broad match
**cdc clinical trials**	Broad match
**strata software healthcare**	Phrase match (close variant)
**ehr health and medical records system**	Exact match (close variant)
**healthgrades**	Broad match
**study at home during pandemic**	Broad match
**nursing documentation handouts**	Broad match
**clinical application**	Broad match
**patient care report template**	Broad match
**epic healthcare systems**	Exact match
**free personal health record template**	Phrase match (close variant)
**population health studies**	Broad match

**software engineer healthcare**	Broad match
**create hospital discharge papers online**	Broad match
**log in to mychart account**	Broad match
**ehr electronic health**	Exact match
**health care programs in the us**	Broad match
**urmc mychart login**	Broad match
**insync ehr**	Phrase match (close variant)
**volunteer for vaccine trials**	Broad match
**online mha programs no gre**	Broad match
**portal patient test results**	Broad match
**the virus**	Broad match
**mychart online access**	Broad match
**medical records course online**	Broad match
**emr systems for hospitals**	Exact match (close variant)
**epic patient registration**	Broad match
**nextgen practice management user guide**	Broad match
**crm for healthcare**	Broad match
**apply technology and information management tools to supp**	Broad match
**electronic health records certification online**	Phrase match (close variant)
**hospital management system project**	Broad match
**arhena health**	Broad match
**why population health is important**	Broad match
**novant health my chart**	Broad match
**healthcare information system vendors**	Broad match
**health care training programs**	Broad match
**online forms hipaa compliant**	Broad match
**epic medical system training**	Broad match
**electronic health record patient access**	Exact match
**personal health record service**	Exact match (close variant)
**caresuite quickmar training**	Broad match
**medical charting software**	Broad match
**medical appointment booking system**	Broad match

**my personal health record printable**	Phrase match (close variant)
**new study natural immunity**	Broad match
**study medical coding online**	Broad match
**microsoft access medical records database**	Broad match
**health systems manager**	Broad match
**how to get medical records from hospital**	Broad match
**novavax**	Broad match
**how to access blood test results online**	Broad match
**jiva zeomega**	Broad match
**get paid for studies near me**	Broad match
**electronic health records in healthcare**	Exact match (close variant)
**healthe**	Broad match
**medical forms for new patients**	Broad match
**health information companies**	Broad match
**hospital care management**	Broad match
**managing hospital**	Broad match
**mmr titer test**	Exact match
**population health services organization**	Broad match
**populatio health**	Broad match
**virtual clinical research**	Broad match
**healthcare business intelligence**	Broad match
**patient pre registration process**	Broad match
**johnson and johnson vaccine springfield il**	Broad match
**vaccinw**	Broad match
**healthcare mobile solutions**	Broad match
**home health billing software**	Phrase match (close variant)
**livehelth**	Broad match
**how to use epic medical software**	Broad match
**medical training platform**	Broad match
**cehrs certification**	Broad match
**ehr patient portal**	Broad match
**how does ehr improve patient care**	Broad match

**ecw program**	Broad match
**epic emr training 2019**	Broad match
**maderna trials**	Exact match (close variant)
**patient fusion**	Broad match
**where statistical application has greatly influenced or chang**	Broad match
**best study app for medical students**	Broad match
**how to qualify for home health care in texas**	Broad match
**health provider**	Broad match
**cerner healthcare system**	Phrase match (close variant)
**health access programs**	Broad match
**lab electronic health records ehr system**	Phrase match (close variant)
**epic charting system**	Broad match
**telemedicone**	Broad match
**epic ehr**	Phrase match (close variant)
**medical software development**	Broad match
**patient crm software**	Broad match
**form to release medical records**	Phrase match (close variant)
**center of excellence healthcare**	Broad match
**how to learn epic healthcare software**	Phrase match (close variant)
**digital health association**	Broad match
**website for hospital management system**	Broad match
**greenway ehr training**	Phrase match (close variant)
**epec software**	Exact match
**in home care software**	Broad match
**what is practice management software**	Broad match
**hedis overview**	Broad match
**top telehealth platforms**	Broad match
**elm healthcare information service**	Broad match
**medical app development**	Broad match
**epic ehr system**	Exact match
**mychart information**	Broad match
**optum population health**	Broad match

**connecture**	Broad match
**israel study natural immunity**	Broad match
**electronic prior authorization**	Broad match
**emr health care software**	Phrase match (close variant)
**what is the electronic health record**	Exact match
**health care information technology**	Broad match
**free emr training certification**	Broad match
**population health management examples**	Broad match
**novant health people soft**	Broad match
**difference between electronic health records and electronic**	Exact match
**epic systems corporation**	Exact match
**how to access your medical records**	Broad match
**medical records coding course**	Broad match
**delivering health care in america a systems approach 7th ed**	Broad match
**shared medical system**	Broad match
**epic emr demo**	Phrase match (close variant)
**free new patient medical forms pdf**	Phrase match
**research studies near me**	Broad match
**janssen vaccine**	Broad match
**how to learn medical billing and coding for free**	Broad match
**patient interviews**	Broad match
**emr for therapists**	Broad match
**ehr emr software**	Phrase match (close variant)
**phizer study**	Exact match (close variant)
**ehr certification training**	Broad match
**patient partner program**	Broad match
**healthcare it case study**	Broad match
**medical appointment scheduling programs**	Broad match
**healthcare management app**	Broad match
**patient care management pdf**	Broad match
**epic software wisconsin**	Exact match
**iqvia trials**	Broad match

**medical record en español**	Exact match
**antibody titer test results**	Phrase match (close variant)
**healthcare records**	Exact match
**u s healthcare problems and solutions**	Broad match
**pratice fusion**	Broad match
**medical software companies**	Phrase match (close variant)
**cdc vaccination testing**	Phrase match (close variant)
**moderna+**	Broad match
**health information systems**	Broad match
**patient access to ehr**	Exact match
**medical billing system software**	Phrase match (close variant)
**hospital information systems**	Broad match
**athenapractice**	Broad match
**healthcare domain knowledge for business analyst**	Broad match
**cdc natural immunity study 2022**	Broad match
**my chart health records**	Broad match
**healthcare ehr systems**	Exact match (close variant)
**carevue charting**	Broad match
**digital health policy**	Broad match
**clinical research trials near me**	Broad match
**immune system test questions**	Phrase match (close variant)
**health systems management**	Exact match (close variant)
**mvhs mychart login**	Broad match
**how to get a health screening**	Broad match
**care management vendors**	Broad match
**remdesiver**	Broad match
**simplify emr**	Phrase match (close variant)
**population health management certificate**	Broad match
**epiccare**	Broad match
**healthcare analytics certificate program**	Broad match
**patient notes software**	Broad match
**what is medical record**	Exact match

**emr documentation guidelines**	Broad match
**patient appointment scheduler**	Broad match
**epic medical research**	Broad match
**how to take patient vital signs**	Broad match
**top clinical information systems executive**	Broad match
**one year health care programs**	Broad match
**digital health business and technology**	Broad match
**health record software**	Phrase match (close variant)
**therapy emr software**	Phrase match (close variant)
**study planner app for medical students**	Broad match
**medical websites for information webmd**	Phrase match (close variant)
**electronic health records system**	Exact match
**patient encounter form template**	Broad match
**epicare**	Broad match
**epic computer program for hospitals**	Broad match
**clinical information system**	Broad match
**set up patient portal**	Broad match
**ehr project manager**	Broad match
**what is a patient health record**	Exact match
**chiropractor software**	Broad match
**care management services**	Broad match
**partners population health management**	Broad match
**hiv functional cure clinical trials**	Broad match
**johns hopkins health information**	Phrase match (close variant)
**medical manager software manual**	Broad match
**download healow app**	Broad match
**measurement based care software**	Broad match
**hospital management model**	Broad match
**medical record fees**	Exact match
**soarian tutorial**	Broad match
**patient check in system**	Broad match
**emr for therapists**	Phrase match (close variant)

**healthcare advocacy programs**	Broad match
**medical assisting with ms k**	Broad match
**custom medical software development**	Broad match
**mychart trinityhealth org**	Broad match
**electronic health records benefits and challenges**	Exact match
**health medical records**	Broad match
**moderna clinical trials phase 3**	Broad match
**diagnotes**	Broad match
**big data and healthcare**	Broad match
**examples of disease management programs**	Broad match
**elixia clinical research**	Broad match
**mychart home**	Broad match
**population health plan**	Broad match
**best healthcare crm software**	Phrase match (close variant)
**johnson and johnson vaccine**	Broad match
**athena emr tutorial**	Broad match
**syapse**	Broad match
**telehealth case study**	Broad match
**health data services**	Broad match
**medical record coding auditor**	Phrase match
**outpatient module**	Broad match
**fitness management software**	Broad match
**is the pandemic over**	Broad match
**medical exam room checklist**	Broad match
**emr programs**	Exact match (close variant)
**free electronic medical records training**	Phrase match
**electronic health records project**	Exact match
**health care review management**	Broad match
**medical research studies in st louis**	Broad match
**geriatric health assessment**	Broad match
**who is epic**	Exact match
**epic health wisconsin**	Broad match

**software for doctors**	Broad match
**hospital electronic medical records**	Broad match
**hospital in usa**	Broad match
**vaccine rewards management software**	Broad match
**mychar**	Broad match
**health population management**	Broad match
**welkin health**	Broad match
**health first app**	Broad match
**patient log**	Broad match
**healthcare provider data**	Broad match
**altered medical records**	Phrase match (close variant)
**free patient portal**	Broad match
**patient management apps**	Broad match
**how to use epic software**	Exact match
**patients medical records**	Exact match
**epic charting system for nurses training**	Broad match
**fusion web clinic login**	Broad match
**medical records company**	Exact match
**excelcare health management**	Broad match
**epic computer program**	Exact match
**what is emr systems**	Exact match
**hospital software companies**	Broad match
**best lab software**	Broad match
**labdaq**	Broad match
**medical software**	Exact match (close variant)
**health informatics terminology**	Broad match
**psychiatry emr templates**	Broad match
**epic system training certification**	Broad match
**medical records retrieval**	Broad match
**emr systems in healthcare**	Exact match (close variant)
**population health manager careers**	Broad match
**hospital operating systems**	Broad match

**evident charting system**	Broad match
**novavax approval**	Broad match
**microsoft medical software**	Phrase match (close variant)
**documentation for nurses**	Broad match
**medical screening exam**	Broad match
**meritus mychart login**	Broad match
**doctors forms for patients**	Broad match
**emrs**	Exact match (close variant)
**checklist for electronic health records**	Exact match
**nccare360 unite us**	Broad match
**telehealth**	Broad match
**clinic and hospital management**	Broad match
**project management for healthcare**	Broad match
**advancedmd ehr tutorial**	Broad match
**practice fusion emr**	Broad match
**healthcare training software**	Broad match
**cleveland clinic study natural immunity**	Broad match
**medication review software**	Broad match
**hospital management information system**	Broad match
**healthcare leadership training programs**	Broad match
**unite us sdoh**	Broad match
**kipu health log in**	Broad match
**enli**	Broad match
**epic healthcare software certification**	Broad match
**disease management software**	Broad match
**access medical records online**	Broad match
**ehr practice test**	Broad match
**mychart customer service**	Broad match
**functional medicine software**	Broad match
**medical emr**	Exact match
**paid research studies near me**	Broad match
**healthcare emr**	Exact match

**patient management software free**	Phrase match (close variant)
**eclinicalworks**	Broad match
**top healthcare it companies**	Broad match
**healthcare payer software**	Phrase match (close variant)
**free hospital records patients**	Broad match
**online patient registration**	Broad match
**medication adherence program**	Broad match
**how to schedule patients effectively**	Broad match
**medical questions answered free**	Broad match
**online medical assessment**	Broad match
**digital medicine companies**	Broad match
**training programs for healthcare professionals**	Broad match
**youtube epic emr**	Exact match
**point click care care plans**	Broad match
**sunrise charting system**	Broad match
**how do emrs benefit population health management**	Broad match
**2 step tb test process**	Broad match
**athena helth**	Broad match
**health care management system**	Exact match (close variant)
**practice management software for healthcare**	Exact match (close variant)
**athena health new patient portal**	Broad match
**what is cdi in healthcare**	Broad match
**find paid clinical trials near me houston**	Broad match
**mrna vaccine studies**	Broad match
**employee health software systems**	Broad match
**vaccine test record management**	Phrase match
**hospital sitter program**	Broad match
**medical report services**	Broad match
**patient web portals**	Broad match
**athna health**	Broad match
**electronic health records standards**	Exact match
**medical coder online program**	Broad match

**physician time study**	Broad match
**free medical billing and coding course**	Broad match
**healthcare crm companies**	Broad match
**reliable emr**	Phrase match (close variant)
**mckesson home health software**	Broad match
**health insight**	Broad match
**patient portal registration**	Broad match
**clinical laboratory system**	Broad match
**management in hospital**	Broad match
**healthcare payer software**	Broad match
**clinical decision support system pdf**	Broad match
**modernizing medicine practice management software**	Phrase match (close variant)
**regen cov clinical trial**	Broad match
**sign in to mychart**	Broad match
**how to use epic healthcare system**	Exact match
**sample health assessment form**	Broad match
**medical software packages**	Exact match (close variant)
**allscripts professional ehr tutorial**	Broad match
**trinity health mychart sign up**	Broad match
**pra health sciences clinical trials**	Broad match
**digital health studies**	Broad match
**hospital software**	Broad match
**health information**	Exact match (close variant)
**patient medical record**	Broad match
**medical health questionnaire**	Broad match
**new patient**	Broad match
**iqvia current studies**	Broad match
**certified electronic health records specialist practice exam**	Broad match
**digital healthcare platform**	Exact match (close variant)
**emr epic**	Exact match
**medical records emr**	Exact match (close variant)
**what is healthcare data**	Broad match

**hospital management software download free**	Phrase match (close variant)
**types of medical software**	Broad match
**free new patient registration form**	Broad match
**clinical software**	Broad match
**best emr for psychiatry**	Phrase match (close variant)
**view my medical records online free**	Broad match
**epic software training online**	Broad match
**database project on hospital management system**	Broad match
**meaningful use of emr**	Exact match
**vacsen**	Broad match
**what is pop health**	Broad match
**patient relationship management**	Broad match
**covidstudy**	Exact match (close variant)
**wtc health program login**	Broad match
**vaccine credential**	Broad match
**medical office software systems**	Broad match
**what is a nuclear scan**	Broad match
**healthcare management industry**	Broad match
**health care information management**	Exact match (close variant)
**physical therapy ehr**	Broad match
**ehr template**	Broad match
**priviahealth**	Broad match
**ehr mental health**	Phrase match (close variant)
**chronic care management software**	Phrase match (close variant)
**issues in healthcare**	Broad match
**electronic health records issues**	Exact match
**sih mychart login**	Broad match
**ehr certification**	Broad match
**care management software**	Exact match (close variant)
**clinical research studies**	Broad match
**patient medical education**	Phrase match
**millennia healthcare**	Broad match

**healthcare informatics programs**	Broad match
**medical information research san antonio tx**	Broad match
**healthcare software providers**	Broad match
**myhealthone pre registration**	Broad match
**how to see patients faster**	Broad match
**clinical education software**	Broad match
**patient registration online**	Broad match
**cerner practice charting**	Broad match
**outpatient tracking system**	Broad match
**medical records management**	Broad match
**healthcare software jobs**	Broad match
**healthcare management programs**	Exact match (close variant)
**electronic health records history**	Exact match
**consent management healthcare**	Broad match
**medical billing software**	Broad match
**epic software**	Exact match
**digital health software**	Exact match (close variant)
**med save**	Broad match
**disease management vendors**	Broad match
**point click care skilled nursing login**	Broad match
**ehr**	Exact match
**j and j vaccine**	Broad match
**onco emr login**	Broad match
**hospital billing systems**	Broad match
**health catalyst office locations**	Broad match
**medical operative report**	Phrase match (close variant)
**phr**	Phrase match (close variant)
**value based care software**	Broad match
**remote patient monitoring platforms**	Broad match
**personal health record for patient**	Broad match
**university medical portal**	Broad match
**epic emr system cost**	Exact match

**mynovant org login**	Broad match
**medical records coding manager**	Broad match
**patient portal setup account**	Broad match
**what is medical manager software**	Exact match (close variant)
**behavioral health ehr software**	Phrase match (close variant)
**epic certification program**	Broad match
**health care system management**	Exact match (close variant)
**vaccine clinical trials**	Exact match (close variant)
**patient managment**	Broad match
**health services organization**	Broad match
**what is health systems management**	Exact match (close variant)
**emr for psychiatry**	Phrase match (close variant)
**patient financing programs**	Broad match
**epic medical program**	Exact match
**medical office software**	Exact match (close variant)
**emr epic**	Phrase match (close variant)
**hopkins study lockdowns**	Broad match
**paid clinical trial**	Broad match
**case studies healthcare**	Broad match
**applying quality management in healthcare a systems appro**	Broad match
**clinical software companies**	Broad match
**medical records patient**	Broad match
**data quality healthcare**	Broad match
**moderna vaccine study**	Exact match (close variant)
**epic electronic health record software**	Exact match
**learn how to use epic emr**	Exact match
**free hospital management software**	Phrase match (close variant)
**school health management software**	Phrase match
**clinician software**	Broad match
**e medical records**	Broad match
**medical management app**	Broad match
**sweden mrna vaccine study**	Broad match

**population health applications**	Broad match
**free hospital management software india**	Phrase match (close variant)
**e health**	Broad match
**health management apps**	Exact match (close variant)
**healthcare management description**	Broad match
**free online emr course**	Broad match
**praxis emr**	Phrase match (close variant)
**healthcare analyst**	Broad match
**physical therapy emr software**	Phrase match (close variant)
**interpoint health**	Broad match
**emr for physical therapy**	Phrase match (close variant)
**it health**	Broad match
**mychart martin health mychart**	Broad match
**teach yourself medical coding**	Broad match
**emr record**	Exact match (close variant)
**healthwire**	Broad match
**medical records certification form**	Phrase match (close variant)
**electronic health records and quality of care**	Exact match
**counseling practice software**	Broad match
**are there any new variants of concern**	Broad match
**ministry of health pcr test**	Broad match
**pfizer clinical trial kids**	Phrase match (close variant)
**wvu mychart login**	Broad match
**kareo**	Broad match
**physician software**	Broad match
**improving access to healthcare**	Broad match
**caradigm**	Broad match
**myhealthone registration**	Broad match
**nextmd patient portal app**	Broad match
**epic emr tutorial**	Broad match
**how to get mental health records**	Broad match
**what is an electronic medical record emr system**	Exact match

**my health one login**	Broad match
**home care software**	Broad match
**healthcare lms**	Broad match
**electronic health record ehr credentialed trainer**	Phrase match
**who created epic emr**	Exact match
**electronic health records association**	Exact match
**promed**	Broad match
**medical practice key performance indicators**	Broad match
**health care incentives programs**	Broad match
**cerner training courses**	Broad match
**healthcare data collection**	Broad match
**technidata medical software**	Broad match
**healthcare electronic records**	Exact match
**eclinical emr**	Phrase match (close variant)
**patient communication software**	Broad match
**patient data management**	Broad match
**allscripts emr tutorial**	Broad match
**mychart jefferson healthcare**	Broad match
**electronic medical records training free**	Broad match
**electronic health records what's in it for everyone**	Exact match
**hospital appointment scheduling software**	Broad match
**electronic medical records**	Exact match
**phreesia pre registration**	Broad match
**occupational health emr**	Broad match
**questions to ask your physician**	Broad match
**healthcare management analytics**	Broad match
**patient medical record release form**	Phrase match (close variant)
**ehr for physicians**	Exact match
**excel medical spreadsheets**	Broad match
**medical data extraction jobs**	Broad match
**austin medical study**	Broad match
**radiology information system**	Broad match

**medication adherence software**	Broad match
**basic physical exam forms printable**	Broad match
**outpatient monitoring**	Broad match
**screening health assessment**	Broad match
**health information specialist**	Broad match
**healthcare leadership development program**	Broad match
**mymedical**	Broad match
**cdac healthcare**	Broad match
**medicare connected care program**	Broad match
**epic electronic**	Exact match
**hospital management system website**	Broad match
**stage 3 meaningful use**	Broad match
**medical software download**	Phrase match (close variant)
**medical record database**	Broad match
**open source health software**	Broad match
**clinical lab software**	Broad match
**electronic health records jobs**	Exact match
**pfizer side effects released**	Broad match
**chances of surviving icu**	Broad match
**medical software system**	Broad match
**your optimum healthcare member portal**	Broad match
**kansas city research studies**	Broad match
**data analytics in population health management**	Broad match
**hospitalist program**	Broad match
**digital health study**	Broad match
**scientific trials near me**	Broad match
**epic tutorials for nurses**	Broad match
**medical practice management software systems**	Exact match (close variant)
**my baycare org**	Broad match
**accuro**	Broad match
**medical record**	Exact match
**epic medical records tutorial**	Broad match

**pfizer clinical trial documents**	Phrase match (close variant)
**medical health record**	Exact match
**tuberculosis testing**	Exact match (close variant)
**mychart trinity health org mychart**	Broad match
**find paid clinical trials near me**	Broad match
**business intelligence medical**	Broad match
**electronic health records in the news**	Exact match
**mobihealth**	Broad match
**emr systems training**	Phrase match (close variant)
**veterans electronic health records ehr system**	Phrase match (close variant)
**patient check in**	Broad match
**clinical information systems vs electronic health records**	Exact match
**workplace health promotion programs**	Broad match
**patient registry software united states**	Phrase match (close variant)
**online patient scheduling system**	Broad match
**medical record classes online**	Broad match
**interacting with patients**	Broad match
**johns hopkins study on lockdowns**	Broad match
**medfusion intuit**	Broad match
**emr test questions**	Broad match
**free emr practice test**	Broad match
**healthcare analytics**	Broad match
**aprima medical software**	Broad match
**online health assessment free**	Broad match
**how to use sigmacare**	Broad match
**care coordination software**	Broad match
**electronic health record policy**	Broad match
**php hospital management system**	Phrase match (close variant)
**free medical encounter forms**	Broad match
**medent training classes**	Broad match
**free health care training programs**	Broad match
**medical questions free**	Broad match

**issues facing healthcare**	Broad match
**better you virtual health**	Broad match
**vaccine research**	Broad match
**medical record of patient**	Exact match
**healthcare it programs**	Broad match
**pediatric emr software**	Phrase match (close variant)
**epic emr review**	Broad match
**mynm**	Broad match
**laboratory information systems**	Broad match
**patient access team lead**	Broad match
**ehr software**	Exact match (close variant)
**patient health portal login**	Broad match
**medical credentialing software**	Broad match
**ehr behavioral health**	Phrase match (close variant)
**kipu health emr login**	Broad match
**epic emr training manual**	Broad match
**implementing electronic health records**	Exact match
**epic software healthcare training manual**	Broad match
**medical check up checklist**	Broad match
**medical records online**	Exact match
**health medical records**	Exact match
**my medical chart**	Broad match
**pfizer vaccine document**	Broad match
**mental health record keeping**	Broad match
**remote hedis clinical data abstractor**	Broad match
**revenue cycle management software**	Broad match
**free emr systems**	Broad match
**my uh care personal health record**	Phrase match (close variant)
**chiropractic computer software**	Broad match
**medical records online classes**	Broad match
**recommendation for hospital management system**	Broad match
**database in healthcare**	Broad match

**free medical record search**	Broad match
**health information management program**	Broad match
**caretracker emr**	Broad match
**pfizer clinical study**	Broad match
**medical notes template**	Broad match
**epic health record software**	Exact match
**epic software medical**	Exact match
**population healthcare**	Broad match
**psychiatry emr systems**	Phrase match (close variant)
**primary care doctor**	Broad match
**electronic medical records course**	Broad match
**epic software company**	Exact match
**ehr system**	Exact match
**hospital information**	Broad match
**nurse led care**	Broad match
**screenings for women's health**	Broad match
**clinical data abstractor**	Broad match
**health assessment software**	Phrase match (close variant)
**chronic care management call script**	Broad match
**digital health initiatives**	Broad match
**emr systems training free**	Broad match
**medical histories**	Exact match (close variant)
**epic billing training**	Broad match
**cpoe**	Broad match
**healthcare statement**	Broad match
**medical marketing software**	Broad match
**online medical appointment scheduling software**	Broad match
**pfizer report**	Broad match
**clinical peer review template**	Broad match
**digital health community**	Broad match
**how to run a hospital successfully**	Broad match
**uhmychart org mychart**	Broad match

**castlight**	Broad match
**what are electronic medical records**	Exact match
**health record systems**	Exact match
**healthcare workflow**	Broad match
**free ehr training online**	Broad match
**best ehr for small practice**	Phrase match (close variant)
**hospital data management**	Broad match
**cerner software training**	Broad match
**home health care software**	Phrase match
**goal of population health**	Broad match
**gsk vaccine**	Broad match
**school health knowledge base software**	Broad match
**meditech electronic medical record system**	Phrase match (close variant)
**nanthealth**	Broad match
**free epic training modules**	Broad match
**medicare health risk assessment questionnaire**	Broad match
**health dashboard**	Broad match
**emr systems for therapy**	Broad match
**point and click emr**	Phrase match (close variant)
**medisoft medical billing software**	Broad match
**blank medical forms**	Broad match
**medicare wellness questionnaire**	Broad match
**online disease learning management system**	Broad match
**case studies in health information management**	Broad match
**forward medical**	Broad match
**how to set up mychart**	Broad match
**hospital information management system hims**	Broad match
**world council for health vaccines**	Broad match
**electronic health records classes**	Phrase match (close variant)
**www myhealthone com**	Broad match
**free medical billing training online**	Broad match
**medical database systems**	Broad match

**clinical decision support systems**	Broad match
**medical records chart**	Broad match
**patient registration systems healthcare**	Broad match
**health questionnaire example**	Broad match
**emmi patient education**	Broad match
**best clinic software**	Broad match
**carelogic training**	Broad match
**hospital management system web based project**	Broad match
**himss**	Broad match
**health info**	Broad match
**access care management**	Broad match
**digital health services**	Broad match
**my pinnaclehealth org**	Broad match
**medical record templates**	Broad match
**ehr for private practice**	Phrase match (close variant)
**health one pass**	Broad match
**mayo clinic screening**	Broad match
**telemedicine questionnaire**	Broad match
**training emr**	Broad match
**healthcare referral management system**	Phrase match (close variant)
**health care analyst**	Broad match
**physician credentialing software**	Broad match
**emr systems epic**	Exact match
**medical referral tracking system**	Broad match
**pfizer dose**	Broad match
**best healthcare software**	Broad match
**top geriatric doctors near me**	Broad match
**medical record program**	Broad match
**employee health management software**	Phrase match
**medical weight management programs**	Broad match
**medical billing coding software**	Broad match
**medical assessment scenarios**	Broad match

**online medical scheduling**	Broad match
**free medical software**	Phrase match (close variant)
**population health partners**	Broad match
**free patient registration form template**	Broad match
**therapy software**	Broad match
**health management system**	Exact match (close variant)
**electronic health records ehr software**	Broad match
**ehr software**	Broad match
**ehr certification practice test**	Phrase match (close variant)
**epic online training**	Broad match
**personal health record software**	Broad match
**healthit analytics**	Broad match
**information about healthcare**	Exact match (close variant)
**diabetes education questionnaire**	Broad match
**patient protocol**	Broad match
**novavax usa**	Broad match
**electronic health records benefits**	Exact match
**verisma medical records**	Broad match
**noreply followmyhealth com**	Broad match
**pharmacy management system**	Broad match
**online learning during pandemic**	Broad match
**lilly clinical trials**	Broad match
**ehealth**	Broad match
**clinical requisition**	Broad match
**my online patient portal**	Broad match
**becoming emt certified**	Broad match
**hospital information system united states**	Broad match
**epic electronic medical record system**	Broad match
**paramedic report**	Broad match
**medical case management software free**	Phrase match (close variant)
**xg health solutions**	Broad match
**general health information**	Phrase match (close variant)

**care plan software**	Broad match
**patient education resources**	Broad match
**workflow in healthcare**	Broad match
**via healthcare consulting**	Broad match
**mychart university health**	Broad match
**pfizer side effects released 2022**	Broad match
**emt patient scenarios and answers**	Broad match
**mayo clinic disease information**	Broad match
**therapy billing software**	Broad match
**health performance management**	Broad match
**methodist healthcare patient portal**	Broad match
**wound care emr**	Broad match
**what is epic systems for hospitals**	Exact match
**healthcare credentialing software**	Broad match
**epic medical software tutorial**	Phrase match (close variant)
**urgent care ehr**	Broad match
**medisys login**	Broad match
**epic software healthcare training manual**	Phrase match (close variant)
**medical records**	Exact match (close variant)
**jefferson school of population health**	Broad match
**population health management centers**	Broad match
**tb test form**	Broad match
**patient medical forms**	Broad match
**erp for healthcare**	Broad match
**physicians survey**	Broad match
**hospital discharge papers sample**	Broad match
**use case diagram for hospital management system**	Broad match
**pophealthcare locations**	Broad match
**healthcare compliance software**	Broad match
**optima hospice software**	Broad match
**health information clerk**	Broad match
**pa health wellness provider portal**	Broad match

**patient health record**	Exact match
**myhealthmanagement com**	Broad match
**emr for mental health**	Phrase match (close variant)
**sample patient information sheet**	Broad match
**healthcare analytics platform**	Broad match
**his healthcare**	Broad match
**health program**	Broad match
**vaccine study participants**	Broad match
**nextgen medical billing**	Broad match
**soarian healthcare software**	Phrase match (close variant)
**how to organize medical records**	Exact match (close variant)
**health research**	Broad match
**vaccine record sheet**	Broad match
**envision medical software**	Phrase match (close variant)
**health technology management**	Broad match
**how to develop a hospital management system**	Broad match
**medical crm**	Broad match
**digital health monitoring**	Broad match
**kareo electronic health record**	Broad match
**allscripts training pdf**	Broad match
**moderna vaccine long term side effects**	Broad match
**opd management**	Broad match
**herpes cure 2022**	Broad match
**vaccine nyc**	Broad match
**blank medical record form**	Phrase match
**standards of ehr**	Exact match
**how to register for one health pass**	Broad match
**united healthcare case management program**	Broad match
**healthcare delivery system**	Broad match
**sweden pfizer study**	Broad match
**clinical testing**	Broad match
**iot hospital**	Broad match

**phreesia medical**	Broad match
**medical history forms**	Broad match
**using epic software**	Exact match
**university hospital my chart**	Broad match
**free medical records**	Broad match
**electronic care planning**	Broad match
**emr systems examples**	Broad match
**kepro**	Broad match
**www centrahealth com healthyu**	Broad match
**e prescribing system**	Broad match
**u s health**	Broad match
**asking doctors questions**	Broad match
**healthcare information**	Exact match (close variant)
**telemed**	Broad match
**population health management**	Broad match
**patient fusion login**	Broad match
**mental health emr**	Phrase match (close variant)
**health catalyst vitalware**	Broad match
**wellcentive login**	Broad match
**medcompass assurecare**	Broad match
**health start program**	Broad match
**therapy emr**	Broad match
**activ 6 study**	Broad match
**electronic health records news**	Exact match
**pfizer data**	Broad match
**top healthcare vendors**	Broad match
**health companies**	Broad match
**natural immunity study**	Broad match
**population health management programs**	Exact match (close variant)
**medical records on line**	Exact match
**can you have your immune system tested**	Phrase match (close variant)
**training for emr**	Broad match

**population health manager jobs**	Broad match
**patient advocacy in healthcare**	Broad match
**certified associate in healthcare information and manageme**	Broad match
**my health records**	Broad match
**lms healthcare academy com**	Broad match
**medical records training**	Broad match
**jefferson mychart login**	Broad match
**healthtrust**	Broad match
**conversa health screening lvhn**	Broad match
**pfizer clinical trial documents released**	Broad match
**disease management program**	Broad match
**digital health screening**	Phrase match (close variant)
**surgery appointment setting system**	Broad match
**epic emr software**	Phrase match (close variant)
**point care software**	Broad match
**epic electronic health record system**	Exact match
**patient self check in**	Broad match
**point click care sign in**	Broad match
**healthfinch**	Broad match
**allscripts professional ehr training**	Broad match
**new study on natural immunity**	Broad match
**health care systems**	Broad match
**health information system**	Broad match
**prevounce health**	Broad match
**epic electronic medical record**	Exact match
**american health information management association**	Broad match
**patient forms for doctors**	Broad match
**will pandemic end this year**	Broad match
**medical home program**	Broad match
**best emr for primary care**	Phrase match (close variant)
**healow health and online**	Broad match
**charm health ehr**	Phrase match (close variant)

**continuing medical education software**	Broad match
**new patient health questionnaire**	Broad match
**free epic emr training online**	Phrase match (close variant)
**human service software**	Broad match
**ob gyn emr software**	Broad match
**paid research trials**	Broad match
**practice management software practice**	Broad match
**dermatology ehr**	Broad match
**crm for healthcare industry**	Broad match
**privia health**	Broad match
**medical claim processing software**	Broad match
**e medical records**	Exact match (close variant)
**healthcare facility maintenance software**	Broad match
**how to use epic ehr**	Broad match
**nih mix and match study**	Broad match
**physical therapy billing software**	Broad match
**electronic medical record programs**	Exact match (close variant)
**healthcare or health care**	Broad match
**healt care**	Broad match
**healthcare onboarding software**	Broad match
**healthcare software engineer**	Phrase match (close variant)
**hospital service management**	Broad match
**health records course online**	Exact match (close variant)
**what is care management**	Broad match
**medinotes**	Broad match
**research studies in houston**	Broad match
**partners health management**	Broad match
**epic charting training for nurses**	Broad match
**patient application**	Broad match
**magnified health systems**	Broad match
**soarian scheduling**	Broad match
**how to create a patient portal**	Broad match

**what is epic software for hospitals**	Exact match
**epic healthcare management system**	Phrase match (close variant)
**online electronic medical records**	Exact match
**what is a medical record**	Exact match
**micromedex**	Broad match
**bnt162b2 clinical trial**	Broad match
**master patient index specialist**	Broad match
**electronic medical record vs electronic health record**	Exact match
**healthy clinical studies**	Broad match
**new patient registration form**	Broad match
**healthrecords**	Broad match
**population health tool**	Exact match (close variant)
**free online ehr training courses**	Broad match
**home health computer software**	Phrase match (close variant)
**medical information & research orlando fl**	Phrase match (close variant)
**pm ehr is**	Broad match
**walgreens vaccination records**	Broad match
**ecompliance**	Broad match
**how does emr work**	Phrase match (close variant)
**incomplete medical records**	Broad match
**sign up for mychart online**	Broad match
**medical billing app**	Broad match
**currenthealth**	Broad match
**health management app**	Broad match
**practice partner**	Broad match
**my medical record**	Phrase match (close variant)
**epic training cheat sheets**	Broad match
**what is eclinicalworks**	Broad match
**annual health screening recommendations**	Broad match
**public health vs population health**	Broad match
**developing medical software**	Broad match
**crm for clinics**	Broad match

**medical billing and coding online programs**	Broad match
**medical software programmer**	Broad match
**standardized nursing terminology**	Broad match
**patient health records online**	Exact match
**what is epic hospital**	Broad match
**social determinants of health screening tool pdf**	Broad match
**healow health**	Broad match
**pre screening for surgery**	Broad match
**sign in to mychart account bsw**	Broad match
**medical care center**	Broad match
**free chronic care management software**	Phrase match (close variant)
**patient intake form**	Broad match
**population care management**	Broad match
**patient chart**	Broad match
**medical outreach program**	Broad match
**any new variants of concern**	Broad match
**labreqs**	Broad match
**treatment forms**	Broad match
**health care computer programs**	Broad match
**clinical trial research**	Broad match
**medical audit software**	Broad match
**secure onco emr**	Phrase match (close variant)
**health risk assessment software**	Broad match
**covidence uk study**	Phrase match (close variant)
**medical records processor jobs**	Phrase match (close variant)
**medication management program**	Broad match
**hospital discharge papers**	Broad match
**free immunization clinic near me**	Broad match
**patient portal benefits**	Broad match
**summa mychart login**	Broad match
**health it companies near me**	Broad match
**emr system**	Broad match

**driven care telemedicine**	Broad match
**electronic patient record**	Exact match
**real time clinical trials**	Broad match
**health education for patients**	Broad match
**his software**	Broad match
**patient access certification**	Broad match
**best medical software**	Broad match
**breaking down silos in healthcare**	Broad match
**mha programs online**	Broad match
**healthcare software documentation**	Phrase match (close variant)
**israeli studies on natural immunity**	Broad match
**moderna pfizer differences**	Broad match
**ehr project manager sr**	Phrase match (close variant)
**population healthy llc**	Broad match
**vaccine studies**	Exact match (close variant)
**cost of ehr systems**	Phrase match (close variant)
**patient profile form**	Broad match
**software hospitals use**	Broad match
**healthcare credentialing software market**	Broad match
**optum population health management**	Broad match
**voalte**	Broad match
**john hopkins antibody study**	Broad match
**proficient healthcare**	Broad match
**patient care practice test**	Broad match
**health information management systems tucson**	Phrase match (close variant)
**discharge plan template**	Broad match
**crm health**	Broad match
**medical appointment software**	Broad match
**healthcare tech companies**	Broad match
**nethealth**	Broad match
**emory healthcare patient portal**	Broad match
**software medical**	Broad match

**medication reconciliation programs**	Broad match
**outpatient practice**	Broad match
**epic software training**	Broad match
**medicalhistory**	Broad match
**healthcare systems**	Broad match
**bi for healthcare**	Broad match
**medical patient**	Exact match (close variant)
**pre op checklist for patients**	Broad match
**remdesivir studies**	Broad match
**citrix emr system**	Phrase match (close variant)
**hospital management system modules**	Broad match
**patience access**	Broad match
**mental health billing software**	Broad match
**vera whole health castlight**	Broad match
**university of miami health portal**	Broad match
**healthcare crm**	Phrase match (close variant)
**care management applications**	Exact match (close variant)
**free ehr software online**	Phrase match (close variant)
**epic healthcare system**	Phrase match (close variant)
**john hopkins studies**	Broad match
**electronic health records practice test**	Broad match
**medical charting forms**	Broad match
**antibody test research**	Broad match
**quest direct labs**	Broad match
**university of kansas health system**	Broad match
**coley's vaccine**	Broad match
**university of chicago clinical trials**	Broad match
**mychart login methodist**	Broad match
**telehealth for windows 10**	Broad match
**healthcare benefits platform**	Broad match
**ehr training video**	Broad match
**viverae health management system**	Phrase match (close variant)

**electronic health records technology**	Exact match
**new patient intake form**	Broad match
**health informatics companies**	Broad match
**electronic healthcare record**	Exact match
**athena health care patient portal**	Broad match
**care manager population health**	Broad match
**how to sign up for patient portal**	Broad match
**infinitt pacs**	Broad match
**patient tracking database**	Broad match
**medical record system**	Exact match
**current health monitor**	Broad match
**epic electronic health records**	Phrase match (close variant)
**advent health cerner login**	Broad match
**medaxion anesthesia**	Broad match
**medical records service**	Phrase match (close variant)
**epic software healthcare demo**	Phrase match (close variant)
**medical data management**	Broad match
**ehr application analyst**	Phrase match (close variant)
**primary care new patient forms**	Broad match
**best medical records software**	Exact match
**what is health platform**	Broad match
**best ehr for small practices**	Broad match
**medisoft medical software**	Phrase match (close variant)
**physical therapy ehr**	Phrase match (close variant)
**healthcare software vendors**	Phrase match (close variant)
**epic ehr software**	Phrase match (close variant)
**carevue**	Broad match
**certified ehr trainer**	Broad match
**nyu langone mychart**	Broad match
**emr for behavioral health**	Broad match
**health management tools**	Exact match (close variant)
**clinical notes software**	Broad match

**nih mixed vaccine study**	Broad match
**epic healthcare**	Broad match
**epic health information system**	Exact match
**eclinical emr**	Broad match
**free medical coding classes online**	Broad match
**medical management system**	Broad match
**allscripts tutorial**	Broad match
**software for doctors office**	Broad match
**healow patient portal registration**	Broad match
**medical records manager**	Exact match (close variant)
**hospital information systems his**	Broad match
**free medical office forms**	Broad match
**allscripts clinical module training manual**	Broad match
**athena hralth**	Broad match
**hospital contract management software**	Broad match
**nextgen ehr training**	Broad match
**covidout study**	Phrase match (close variant)
**health history assessment**	Broad match
**health insurance crm**	Broad match
**optum phs**	Broad match
**healthcare info**	Exact match (close variant)
**vaccine analysis**	Broad match
**epic medical software**	Exact match
**best websites for medical information**	Phrase match (close variant)
**epic electronic health records**	Broad match
**best home health software**	Phrase match (close variant)
**what is health management information system**	Phrase match (close variant)
**cehrs**	Broad match
**personal medical record form**	Broad match
**practice fusion emr cost**	Phrase match (close variant)
**what programs are available to support the aging population**	Broad match
**personal health record**	Broad match

**health care software**	Broad match
**lilly cares patient assistance program**	Broad match
**medical case studies for students**	Broad match
**healthcare data companies**	Broad match
**athena billing training**	Broad match
**free emr templates**	Broad match
**meditech charting for nurses**	Broad match
**research studies in miami**	Broad match
**health plan management system**	Phrase match (close variant)
**healthcare crm software**	Broad match
**what is titer testing**	Phrase match
**health record vs medical record**	Exact match
**pareto population health management practice**	Broad match
**list of emr systems**	Broad match
**what is epic in healthcare**	Broad match
**using epic**	Exact match
**covide updates**	Broad match
**care4 charting system**	Broad match
**upic software**	Exact match
**epic electronic health record**	Exact match
**chart retrieval**	Broad match
**pfizer clinical trial data**	Broad match
**health risk assessment questionnaire pdf**	Broad match
**medical records full time**	Broad match
**software for medical records**	Exact match (close variant)
**free epic training modules courses**	Broad match
**epic emr training video**	Broad match
**updox sign in with practice fusion**	Broad match
**app for clinic**	Broad match
**billing medical**	Broad match
**healthcare information management system**	Phrase match (close variant)
**best medical lab software**	Broad match

**my chart patient portal**	Broad match
**strategic healthcare program**	Broad match
**test for tb screening**	Exact match (close variant)
**the medical manager software**	Exact match (close variant)
**what is patient advocacy in healthcare**	Broad match
**telehealth study**	Broad match
**medical records requirements by state**	Phrase match (close variant)
**free printable patient education handouts**	Broad match
**healthcare population**	Broad match
**community health programs**	Broad match
**health communication programs**	Broad match
**best medical billing software for home based business**	Phrase match (close variant)
**navinet**	Broad match
**ehr electronic health record**	Broad match
**electronic patient registration**	Broad match
**intouch health**	Broad match
**epic training manual**	Broad match
**nextgen ehr**	Phrase match (close variant)
**free practice management software**	Broad match
**builtin health platform**	Broad match
**ims medical software training**	Broad match
**medical laboratory system**	Broad match
**health information management department**	Broad match
**healthcare saas companies**	Broad match
**medical reviewer**	Broad match
**electronic health records program**	Exact match
**electronic health record ehr credentialed trainer**	Broad match
**saas healthcare companies**	Broad match
**epic optime tutorial**	Broad match
**sample patient assessment**	Broad match
**how to improve patient flow in clinic**	Broad match
**second opinion medical**	Broad match

**online medical appointment booking system**	Broad match
**pfizer vs astrazeneca**	Broad match
**blood test results**	Broad match
**clinic management software free download**	Phrase match (close variant)
**sweden study pfizer**	Broad match
**portal patient**	Broad match
**experity emr download**	Broad match
**augmedix**	Broad match
**free medical forms**	Broad match
**kareo system**	Broad match
**population health manager**	Broad match
**tb testing**	Phrase match (close variant)
**statistics software for medical research**	Broad match
**e health services**	Broad match
**health pathway**	Broad match
**hospice ehr**	Broad match
**two step tb test timeline**	Broad match
**remote medical records medical data reviewer**	Broad match
**university hosptial**	Broad match
**medical office scheduling software**	Broad match
**view my health records**	Broad match
**do you have to be vaccinated to go to boston**	Broad match
**mychart novant**	Broad match
**epic emr training online course**	Broad match
**emr software**	Broad match
**my health record online**	Phrase match (close variant)
**healthcare app for patients**	Broad match
**epic electronic medical records**	Exact match
**population health questions**	Broad match
**clinical study trials**	Broad match
**moderna trials**	Broad match
**personal health record online**	Phrase match (close variant)

**my borgess health sign in**	Broad match
**advantages of electronic medical records**	Broad match
**healthcare survey questions**	Broad match
**testing for t cell immunity**	Phrase match (close variant)
**population health analytics examples**	Broad match
**medical business intelligence**	Broad match
**how to get your heart checked**	Broad match
**healthcare workflow automation**	Broad match
**clinic management software free download full version**	Phrase match (close variant)
**medical software programs**	Exact match (close variant)
**candelis**	Broad match
**certification of medical records form**	Phrase match (close variant)
**health benefits software**	Broad match
**palliative care software**	Broad match
**electronic medical records knowledge**	Exact match
**ehr healthcare**	Exact match
**ehr testing**	Broad match
**accuro medical software**	Broad match
**how to use epic emr system**	Broad match
**what is population health in healthcare**	Broad match
**patient database software**	Broad match
**emr systems**	Broad match
**netsmart**	Broad match
**health administration programs**	Broad match
**medhost charting**	Broad match
**patient medical forms**	Phrase match
**electronic health records and meaningful use**	Exact match
**epic radiant certification**	Broad match
**medical billing api**	Broad match
**scid screening**	Broad match
**counselor software**	Broad match
**population health analytics**	Broad match

**saas for doctors**	Broad match
**healthcare scheduling software**	Broad match
**healthcare management companies near me**	Broad match
**philips health suite digital platform**	Phrase match (close variant)
**atgena health**	Broad match
**medhost**	Broad match
**population health organization**	Broad match
**patient record**	Exact match
**medical management system**	Exact match (close variant)
**what are the 4 components of population health**	Broad match
**clinical studies**	Broad match
**patient onboarding software**	Broad match
**electronic health records ehr system**	Exact match
**epic medical software training**	Broad match
**lark health**	Broad match
**santa clara study**	Broad match
**jefferson my chart portal**	Broad match
**how to use the epic system**	Exact match
**medical form software**	Broad match
**first responder training online free**	Broad match
**medical records samples**	Broad match
**healthedge**	Broad match
**healthcare marketing software**	Broad match
**healthcare vendor management software**	Phrase match (close variant)
**online patient scheduling**	Broad match
**healthcare informatics companies**	Broad match
**health care information services**	Broad match
**medical software reviews**	Phrase match (close variant)
**what is health information management system**	Broad match
**resident learning management system**	Broad match
**covisafe test**	Broad match
**how to use epic**	Exact match

**multiple symptom checker**	Broad match
**healthcare cmms software**	Phrase match (close variant)
**limitations of electronic health records**	Exact match
**doctor office software**	Phrase match (close variant)
**hospital concierge programs**	Broad match
**johns hopkins immunity study**	Broad match
**hospital software system**	Exact match (close variant)
**healthrecord**	Exact match
**patient reported outcome measures**	Broad match
**electronic health records journal articles**	Exact match
**himms**	Broad match
**how to organize medical records**	Broad match
**what is an electronic health record**	Exact match
**free emr training**	Exact match
**who owns epic emr**	Exact match
**test laboratory**	Broad match
**free clinical notes software**	Broad match
**what is meaningful use of ehr**	Exact match
**tb test forms**	Broad match
**health care survey questions examples**	Broad match
**ahrq program**	Broad match
**healthagen**	Broad match
**how can the growing use of personal health records be used**	Broad match
**atlanta clinical trials**	Broad match
**where to study during pandemic**	Broad match
**ehr updates**	Exact match
**learn epic charting system**	Broad match
**surescripts competitors**	Broad match
**findings announced from uk study that purposefully infected**	Phrase match (close variant)
**medical patient registration forms**	Broad match
**mental health ehr**	Phrase match
**study health services**	Broad match

**programs for cancer patients**	Broad match
**disease management apps**	Broad match
**nana healthcare management**	Broad match
**medical billing programs**	Broad match
**doctor questionnaire**	Broad match
**electronic health and medical records**	Exact match
**emr systems for therapy**	Phrase match (close variant)
**sample medical records**	Phrase match (close variant)
**personal health record companies**	Broad match
**electronic medical record training**	Broad match
**population health initiatives**	Broad match
**types of electronic medical records**	Exact match
**medical learning management system**	Broad match
**report medical**	Broad match
**how to get epic certification**	Broad match
**medical consulting companies**	Broad match
**tb test diagnosis**	Broad match
**medical forms for patients**	Broad match
**creating india's digital health ecosystem**	Broad match
**cov002 vaccine trial**	Phrase match (close variant)
**what is mmis in healthcare**	Broad match
**healthviewx**	Broad match
**clinical data registries**	Broad match
**electronic health records for students**	Exact match
**carevoyant**	Broad match
**free medical record**	Broad match
**moderna clinical study**	Broad match
**scrum in healthcare**	Broad match
**ehr advantages**	Broad match
**medical image management**	Broad match
**how to check medical result online**	Broad match
**aprima medical software training**	Broad match

**mental health emr systems**	Phrase match (close variant)
**utilization management**	Broad match
**johns hopkins study on natural immunity**	Broad match
**health care resources**	Broad match
**the diabetes management program by livongo**	Broad match
**health information technologists**	Broad match
**epic system for healthcare**	Broad match
**healthcare software consultant**	Broad match
**doxy me app for patients**	Broad match
**cdc natural immunity study**	Broad match
**best lms for healthcare**	Broad match
**phreesia patient check in**	Broad match
**medical records online**	Broad match
**online hospital management system**	Broad match
**free emr software download**	Broad match
**medical records lookup**	Exact match
**view medical records online free**	Exact match
**care compass software**	Broad match
**electronic health records for schools**	Exact match
**e prescribing software**	Broad match
**population health ppt**	Broad match
**hospital software solutions**	Broad match
**ehr mandate**	Exact match
**free medical billing and coding training online**	Broad match
**epic computer system for hospitals**	Broad match
**medical records supervisor**	Broad match
**medical records**	Broad match
**family medical history questionnaire**	Broad match
**health care crisis solutions**	Broad match
**billing and coding software**	Broad match
**ohiohealth mychart account**	Broad match
**novarex vaccine**	Broad match

**medical clinic software**	Broad match
**health ec**	Broad match
**cerner care tracker**	Broad match
**kinser home health documentation**	Broad match
**health information coordinator**	Broad match
**health electronic record**	Exact match
**doctor survey questions**	Broad match
**how to apply for scolarship**	Broad match
**exlinical works**	Broad match
**mychart 24lik**	Broad match
**hospital at home program**	Broad match
**learning central university health system**	Broad match
**home care software providers**	Broad match
**free therapy notes software**	Broad match
**free epic training online courses**	Broad match
**tigerconnect clinical collaboration platform**	Broad match
**ehr monitoring**	Exact match
**what is ehr**	Broad match
**health data analyst**	Broad match
**free medical billing software**	Phrase match (close variant)
**medication program**	Broad match
**what is epic ehr**	Exact match
**athenahealth emr training manual courses**	Phrase match (close variant)
**population health management jobs**	Broad match
**population health programs**	Exact match (close variant)
**johns hopkins lockdown study**	Broad match
**health care scenarios**	Broad match
**eclinicalworks provider training**	Broad match
**searchable doctor database software**	Broad match
**clinical study near me**	Broad match
**new patient form**	Broad match
**health professional program**	Broad match

**electronic health records problems**	Exact match
**eclinical**	Broad match
**emt training**	Broad match
**top 10 ehr systems**	Phrase match (close variant)
**medical practice management software free download**	Phrase match (close variant)
**johns hopkins natural immunity study**	Broad match
**healthcare data service**	Broad match
**hospital bed management systems**	Broad match
**rural health medical program**	Broad match
**digital health benefits**	Broad match
**health literacy software**	Broad match
**athenahealth emr training manual**	Phrase match (close variant)
**standardized patient**	Broad match
**smart hospital solution**	Broad match
**athena healthcare software**	Phrase match (close variant)
**epic ehr**	Broad match
**cehrs practice exam free**	Broad match
**importance of ehr**	Exact match
**health software jobs**	Broad match
**emr vs emr serverless**	Broad match
**cancer navigation programs**	Broad match
**ehrgo**	Phrase match (close variant)
**bed management system in hospital**	Broad match
**care management process**	Broad match
**chartmaxx login**	Broad match
**iweb greenway health**	Broad match
**electronic health record jobs**	Phrase match
**patient engagement vendors**	Broad match
**mental health ehr**	Phrase match (close variant)
**medicare wellness exam checklist**	Broad match
**upmc icu**	Broad match
**epic in healthcare**	Exact match

**medicine study app**	Broad match
**ehr importance**	Exact match
**is there still a pandemic**	Broad match
**ehr for psychiatry**	Broad match
**dr fauci funded chinese lab**	Broad match
**updox competitors**	Broad match
**care coordination solutions**	Broad match
**examples of digital health**	Phrase match (close variant)
**social distancing 2025**	Broad match
**health information records**	Exact match
**electronic health records meaningful use**	Exact match
**hospital workforce management**	Broad match
**patient records management**	Broad match
**how to test immune system**	Phrase match (close variant)
**e health technology**	Broad match
**medical manager software**	Exact match
**patient portal com**	Broad match
**medical it companies**	Broad match
**hospital**	Broad match
**software in medicine**	Broad match
**crm for healthcare providers**	Broad match
**c19 studies**	Exact match (close variant)
**find a hospital patient online**	Broad match
**healthcare it system providers**	Broad match
**software for home health**	Phrase match (close variant)
**top 10 healthcare revenue cycle management companies in**	Broad match
**health record management**	Broad match
**how can i get my medical records**	Exact match (close variant)
**modmed training videos**	Broad match
**ehr certified application analyst ii**	Broad match
**electronic medical record errors**	Exact match
**crm monitoring medical**	Broad match

**epic clinical research**	Broad match
**medical chronology**	Broad match
**clinical software applications**	Broad match
**best emr for pain management**	Phrase match (close variant)
**population health alliance**	Broad match
**hutzel women's hospital medical records**	Broad match
**do i need to get vaccinated to go to cuba**	Broad match
**private check up**	Broad match
**medical errors**	Broad match
**lms for healthcare**	Broad match
**urgent care emr**	Phrase match
**how many days do you have to wait to get tested for covid a**	Broad match
**care plan management**	Broad match
**electronic health records exam**	Exact match
**mychart cleveland clinic**	Broad match
**definition of electronic health record**	Exact match
**health records management program**	Broad match
**medical records system**	Broad match
**free online medical records**	Broad match
**meditech**	Broad match
**medical and health services management**	Broad match
**personal medical records management**	Broad match
**about health information management**	Exact match (close variant)
**ebix health administration exchange provider portal**	Broad match
**hsv 2 cure clinical trials 2021**	Broad match
**software for home care**	Broad match
**general health**	Broad match
**arcadia healthcare**	Broad match
**patient fill out form**	Broad match
**complete care management partners**	Broad match
**electronic health records study guide**	Exact match
**electronic medical records emr**	Exact match

**program health dashboard**	Broad match
**hospital administration certification programs**	Broad match
**patient healthcare information**	Broad match
**importance of stakeholders in healthcare**	Broad match
**free medical assistance program**	Broad match
**hospital management services**	Broad match
**johnson and johnson vaccine near me**	Broad match
**patient portal healow**	Broad match
**vital signs procedure in nursing**	Broad match
**benefits of electronic health records system**	Exact match
**caslight**	Broad match
**epic hospital system training manual courses**	Broad match
**health healow patient portal sign in**	Broad match
**matrix charting system**	Broad match
**hms software for hospitals**	Phrase match (close variant)
**point click care nursing documentation**	Broad match
**pzifer data**	Broad match
**ghana healthcare system**	Broad match
**software for health care**	Exact match (close variant)
**what is emr system**	Exact match (close variant)
**www mychart**	Broad match
**cms meaningful use criteria**	Broad match
**doctor medical software**	Broad match
**medical software companies**	Broad match
**electronic health records systems**	Exact match
**lab software**	Broad match
**clinical pathway software**	Broad match
**pcare software**	Exact match (close variant)
**population health management definitions**	Broad match
**what are meaningful use requirements**	Broad match
**epic emr implementation**	Exact match
**digital health design**	Broad match

**my health one portal hca**	Broad match
**health screening software**	Broad match
**log in my healthevet**	Broad match
**emory health care portal**	Broad match
**epic systems for healthcare**	Exact match
**how to make electronic health record**	Exact match
**epic system software**	Broad match
**free e prescribing for physicians**	Broad match
**epic system**	Exact match
**personal health record system**	Broad match
**patient electronic medical record**	Exact match
**who qualifies for medication therapy management program**	Broad match
**lund university vaccine study**	Broad match
**electronic medical record components**	Exact match
**health risk assessment questionnaire**	Broad match
**medical software sales**	Broad match
**meaningful use ehr incentive program**	Broad match
**new patient registration software**	Exact match (close variant)
**patient health record**	Exact match
**medical document**	Broad match
**my medical records online free**	Broad match
**patient representative**	Broad match
**patient assessment questions**	Broad match
**population health strategies**	Broad match
**healthcare software companies**	Broad match
**telehealth survey**	Broad match
**website hospital management system**	Broad match
**top medical software companies**	Broad match
**health care programs**	Broad match
**emr systems training certification**	Broad match
**health informatics and information management**	Broad match
**free tb testing locations**	Broad match

**online learning during pandemic research**	Broad match
**effective population health management**	Broad match
**therapy notes ehr**	Broad match
**types of personal health records**	Phrase match (close variant)
**healthcare consulting companies**	Broad match
**healthcare practice management**	Broad match
**ehr information**	Broad match
**electronic medical records training**	Broad match
**moderna clinical trials**	Broad match
**patient crm software**	Phrase match (close variant)
**pyxis care management**	Broad match
**diabetes self management questionnaire**	Broad match
**medical software reviews**	Broad match
**medical records manager**	Broad match
**health information technology program**	Broad match
**healthcare automation**	Broad match
**automation in hospitals**	Broad match
**medical records certification**	Broad match
**mercy mychart sign in**	Broad match
**introduction to healthcare management free pdf**	Broad match
**referral management system**	Broad match
**university of michigan health portal**	Broad match
**how to see your medical records online**	Broad match
**ocf epic mychart jefferson la**	Broad match
**survey doctor questions**	Broad match
**design and implementation of health information systems**	Broad match
**bi patient assistance program**	Broad match
**patient record system**	Exact match (close variant)
**kaiser health connect log in**	Broad match
**my patient chart**	Broad match
**medical informatics company**	Broad match
**how can i check my lab results online**	Broad match

**health consulting**	Broad match
**epic program for hospitals**	Broad match
**community health worker program**	Broad match
**electronic health records tutorial**	Exact match
**medical data**	Broad match
**cortex medical management systems**	Phrase match (close variant)
**epic medical records software**	Exact match
**www phreesia**	Broad match
**mit professor vaccine**	Broad match
**electronic medical health records**	Exact match (close variant)
**srs hospital management system**	Phrase match (close variant)
**patient flow software**	Broad match
**health management systems inc**	Broad match
**point click care med pass**	Broad match
**future of electronic health records**	Broad match
**cactus credentialing**	Broad match
**medical appointment scheduling for vaccination**	Broad match
**mental health practice management software**	Phrase match (close variant)
**my chart login**	Broad match
**medical record emr**	Exact match
**patient management platform**	Exact match (close variant)
**medical management**	Broad match
**epic training for medical assistants**	Broad match
**legacy systems in healthcare**	Broad match
**vaccine studies near me**	Broad match
**population health consultants**	Broad match
**medical record review companies**	Phrase match
**healthcare scheduling software**	Phrase match (close variant)
**medical practice management systems**	Exact match (close variant)
**best crm for healthcare**	Broad match
**zhealth ehr**	Phrase match (close variant)
**hospital chargemaster software**	Broad match

**epic ehr training courses**	Broad match
**health screening form printable**	Broad match
**medical patient portals**	Phrase match (close variant)
**moderna studies**	Exact match (close variant)
**phizer documents**	Broad match
**ehr medical systems**	Broad match
**how to get a doctor**	Broad match
**phreesia me**	Broad match
**mychart uva com**	Broad match
**hospital management reports**	Broad match
**software in medical**	Exact match (close variant)
**patient record management**	Broad match
**epic medical software tutorial**	Broad match
**best ehr systems**	Exact match (close variant)
**chartlogic**	Broad match
**medical billing software for home based business**	Broad match
**healthcare it companies near me**	Broad match
**duke mychart sign up now**	Broad match
**who owns health care records**	Exact match
**average cost of ehr system**	Phrase match (close variant)
**remdesivir clinical studies**	Broad match
**health care opportunity program**	Broad match
**mdvip program**	Broad match
**point click care skilled nursing**	Broad match
**soarian charting system**	Broad match
**electronic medical record security**	Exact match
**powerchart**	Broad match
**wellness program health**	Broad match
**digital health management platform**	Exact match
**healthcare crm solutions**	Broad match
**certification programs in healthcare**	Broad match
**enli health**	Broad match

**public health software**	Phrase match (close variant)
**omicron vaccine efficacy pfizer**	Broad match
**advanced health tech**	Broad match
**new health research application**	Broad match
**healthcare erp vendors**	Broad match
**hospital preparedness program**	Broad match
**patient registration software**	Broad match
**software for medical office**	Broad match
**high paying clinical trials**	Broad match
**athena charting system**	Broad match
**medication management programs**	Broad match
**johnson and johnson clinical trials**	Broad match
**thoroughcare**	Broad match
**epic health software training**	Phrase match (close variant)
**free online emr**	Phrase match (close variant)
**who owns epic software**	Exact match
**center for information & study on clinical research participat**	Broad match
**cms requirements for hospitals**	Broad match
**primary care pre visit questionnaire**	Broad match
**healthcare delivery and clinical systems**	Broad match
**iemr**	Broad match
**mybaycare org**	Broad match
**healthcare software**	Broad match
**medical records tech**	Broad match
**emr certifications**	Phrase match (close variant)
**electronic health record benefits**	Exact match
**somos care management**	Broad match
**digital health care company**	Broad match
**access my medical records**	Exact match (close variant)
**homecare homebase charting**	Broad match
**provider care management solutions**	Broad match
**pfizer trial data**	Broad match

**behavioral health software**	Broad match
**medical chart**	Broad match
**epic database for healthcare**	Broad match
**isolation studies**	Broad match
**hospital training software**	Broad match
**obgyn practice management**	Broad match
**free medical practice management software**	Phrase match (close variant)
**epic medical records login**	Exact match
**health promotion programs**	Broad match
**how to get my emt certification**	Broad match
**medical charting systems**	Broad match
**future of pandemic**	Broad match
**printable new patient medical forms**	Phrase match
**pediatric population health management**	Broad match
**ehr reviews**	Broad match
**patient centered software**	Broad match
**patient tracking system in hospital**	Broad match
**home health scheduling tips**	Broad match
**hospital discharge software**	Broad match
**medical coding billing software**	Broad match
**medical tracking spreadsheet**	Broad match
**application health assessment**	Broad match
**healthcare consulting companies in usa**	Broad match
**patient practice**	Broad match
**healthone com**	Broad match
**hospital information management system philippines**	Broad match
**free online medical billing coding certification programs**	Broad match
**pfizer clinical studies**	Broad match
**history of electronic medical records in the healthcare indus**	Exact match
**online health database**	Broad match
**epiccare link login**	Broad match
**neurology ehr**	Broad match

**introduction to healthcare**	Broad match
**freesia medical software**	Broad match
**telemedicine software**	Broad match
**medical mobile monitoring**	Broad match
**medical record systems**	Exact match
**medicat**	Broad match
**software used in medical field**	Broad match
**disease management programs**	Broad match
**hospital platform**	Broad match
**mdsave**	Broad match
**emr medical software**	Broad match
**accessing your own medical records**	Broad match
**emr electronic medical records**	Exact match (close variant)
**printable medical record form**	Phrase match
**emr training video**	Broad match
**pfizer data march 1**	Broad match
**electronic medical records training certification**	Broad match
**patient form**	Broad match
**mdpcp program**	Broad match
**nurse triage software**	Broad match
**free electronic medical record**	Phrase match
**sap in healthcare**	Broad match
**mhealth app usability questionnaire**	Broad match
**electronic health record implementation**	Broad match
**allscripts touchworks**	Broad match
**university health my chart**	Broad match
**ivolution medical systems**	Broad match
**electronic health records and information technology**	Exact match
**therapy software programs**	Broad match
**baptist health south florida telemedicine**	Broad match
**create a patient portal**	Broad match
**hospital service line management**	Broad match

**population health management education**	Broad match
**touchworks**	Broad match
**new natural immunity study**	Broad match
**thorough care**	Broad match
**emr cloud based**	Phrase match (close variant)
**netsmart tutorial**	Broad match
**how to use epic medical software**	Phrase match (close variant)
**epic system for hospitals**	Broad match
**electronic health medical records**	Exact match
**appointment system in health information management**	Broad match
**certified electronic health records specialist practice exam**	Phrase match (close variant)
**epic medical system**	Broad match
**shareeverywhere com**	Broad match
**how to apply for medical online**	Broad match
**health policy**	Broad match
**e prescribing**	Broad match
**do electronic medical records improve quality of care**	Exact match
**free online healthcare certificate programs**	Broad match
**therapy ehr systems**	Broad match
**cov boost study**	Broad match
**software used in hospitals**	Exact match (close variant)
**how to use epic emr system**	Exact match
**medical trials for money**	Broad match
**electronic helath records**	Exact match
**patient feedback**	Broad match
**population health certification online**	Broad match
**patient number**	Broad match
**applied digital health**	Phrase match (close variant)
**medassets**	Broad match
**free health assessments**	Broad match
**new clinical trials 2022**	Broad match
**medical lab software**	Broad match

**jane app scheduling**	Broad match
**patient electronic health records**	Exact match
**medical document management**	Broad match
**practice fusion**	Broad match
**online patient registration software**	Phrase match (close variant)
**current health**	Broad match
**epic healthcare systems training**	Broad match
**health management news**	Broad match
**hospital information management**	Broad match
**medical physical exam**	Broad match
**what are medical management programs**	Broad match
**zalenko protocol**	Broad match
**acess to care**	Broad match
**epic programs**	Broad match
**medical patient advocate**	Broad match
**cehrt certification**	Broad match
**pop health solutions**	Broad match
**my one health portal**	Broad match
**apps for healthcare professionals**	Broad match
**free tb test near me**	Phrase match (close variant)
**patient portal solution**	Broad match
**epic health connect**	Broad match
**medical records specialist**	Phrase match (close variant)
**digital health network**	Broad match
**athena software medical**	Phrase match (close variant)
**meditech review**	Broad match
**epic medical charting**	Broad match
**my health record**	Broad match
**hospice optima**	Broad match
**credentialing software for healthcare**	Broad match
**health data platforms**	Broad match
**acuity rating system**	Broad match

**how to get medical records online**	Broad match
**home care agency software**	Broad match
**health care information management**	Broad match
**top medical practice management software**	Phrase match (close variant)
**medical center software**	Broad match
**best hr practices in healthcare industry**	Broad match
**www https health healow com**	Broad match
**workflow issues in healthcare**	Broad match
**detect research study**	Broad match
**department of health guidelines**	Broad match
**goldcare telemed reviews**	Broad match
**population health management framework**	Broad match
**home based palliative care program**	Broad match
**medicaid software**	Broad match
**qhr**	Broad match
**phreesia competitors**	Broad match
**free emr for small practices**	Phrase match (close variant)
**emr medical records**	Exact match
**meditech ehr system**	Phrase match (close variant)
**patient pre registration**	Broad match
**carelogic training videos**	Broad match
**up to date medical software**	Phrase match (close variant)
**what's the new variant**	Broad match
**small medical office software**	Broad match
**health services administration**	Broad match
**pcc medical records**	Broad match
**ice health systems**	Broad match
**cerner healthcare**	Broad match
**electronic health record training**	Phrase match
**paid online studies**	Broad match
**medical advocacy program**	Broad match
**provider data management platform**	Broad match

**healthcare technology companies**	Broad match
**labdaq patient portal**	Broad match
**medical notes software**	Broad match
**what is the reach program in healthcare**	Broad match
**ehr certified application analyst i**	Phrase match (close variant)
**ehr mental health**	Broad match
**what is hhaexchange**	Broad match
**ondek healthcare**	Broad match
**patient medical records request form**	Broad match
**vaccine study**	Phrase match (close variant)
**healthcare workforce management software**	Phrase match (close variant)
**new patient forms print out**	Broad match
**health policy program**	Broad match
**pop health management**	Broad match
**search medical records**	Exact match (close variant)
**online patient scheduler**	Broad match
**unified practice**	Broad match
**healthcare compliance**	Broad match
**home healthcare software solutions**	Broad match
**hospital computer programs**	Broad match
**electronic health records**	Exact match (close variant)
**care management software vendors**	Phrase match (close variant)
**what is the electronic medical record**	Exact match
**provider data management**	Broad match
**patient software**	Exact match (close variant)
**nextgen patient portal**	Broad match
**evident ehr**	Broad match
**clinical excellence program**	Broad match
**merck access program**	Broad match
**patient eligibility verification**	Broad match
**risk management in healthcare**	Broad match
**wellcentive**	Broad match

**healthcare portal company**	Broad match
**lawson healthcare**	Broad match
**world health organization online**	Broad match
**population health manager job description**	Broad match
**testing my patient**	Broad match
**lab medical billing**	Broad match
**free medical coding**	Broad match
**my health portal sign in**	Broad match
**epic software medical**	Phrase match (close variant)
**kidcove study**	Broad match
**free emr training**	Broad match
**pfizer vaccine report**	Broad match
**types of health information systems**	Broad match
**medicare pharmaceutical assistance program**	Broad match
**hospital lab software**	Broad match
**population health trends**	Broad match
**at home medical billing training**	Broad match
**health care coordinator**	Broad match
**emr systems in healthcare**	Broad match
**digital health program**	Broad match
**healthcare crm solutions**	Phrase match (close variant)
**one degree medical**	Broad match
**my health alberta**	Broad match
**healthcare management information**	Broad match
**patient report template**	Broad match
**medisoft**	Broad match
**general survey health assessment**	Broad match
**medical record keeping software**	Exact match
**allscripts charting system**	Broad match
**free medical coding course**	Broad match
**according to cdc guidelines**	Broad match
**phr medical**	Broad match

**kareo vs drchrono**	Broad match
**ibm integration bus healthcare pack**	Broad match
**patients medical rights**	Phrase match (close variant)
**athena emr**	Phrase match (close variant)
**how to use epic emr**	Broad match
**log into mychart**	Broad match
**best clinical trials near me**	Broad match
**mckesson healthcare software**	Phrase match (close variant)
**cvd 19**	Broad match
**how to fill out medical forms**	Broad match
**medical manager software**	Exact match (close variant)
**ehr next gen**	Broad match
**electronic medical records and electronic health records**	Exact match
**infor cloudsuite healthcare**	Broad match
**trucare software**	Broad match
**what is digital health examples**	Broad match
**health links**	Broad match
**free tb testing near me**	Broad match
**patient profile example**	Broad match
**research clinical trials**	Broad match
**emr medical**	Exact match
**patient klara com**	Broad match
**duke clinical trials**	Broad match
**epic ehr training**	Phrase match (close variant)
**healthcare erp**	Broad match
**provider management software**	Broad match
**athena emr training manual**	Broad match
**epic patient software**	Exact match
**point of care information systems**	Broad match
**allscripts care in motion**	Broad match
**mychart login page**	Broad match
**kipu systems login**	Broad match

**clinical study report**	Broad match
**utilization review software**	Broad match
**lab studies near me**	Broad match
**pre op questions to ask patients**	Broad match
**telehealth questionnaire**	Broad match
**healthcare consulting companies near me**	Broad match
**pfizer trial side effects**	Broad match
**documentation in healthcare**	Broad match
**my chart atlantic health org**	Broad match
**kettering my chart login**	Broad match
**healthcare management systems**	Exact match (close variant)
**my health records online**	Phrase match (close variant)
**online patient registration forms**	Broad match
**soarian emr training**	Broad match
**pathology emr**	Broad match
**aerial healthcare**	Broad match
**point click cna charting**	Broad match
**modmed log in**	Broad match
**employee health software**	Phrase match (close variant)
**4c digital health**	Broad match
**hospital dietary software**	Broad match
**open ended questions in healthcare**	Broad match
**medical manager software support**	Broad match
**tb screening**	Exact match (close variant)
**healthcare data management**	Broad match
**patient accounts representative**	Broad match
**hospital management software**	Exact match (close variant)
**hospital administrator program**	Broad match
**my health online**	Broad match
**medisys training**	Broad match
**medical records**	Exact match
**syntellis clinical exchange**	Broad match

**go to mychart**	Broad match
**penn medicine**	Broad match
**hospice emr systems**	Broad match
**managing a hospital**	Broad match
**healthy policy**	Broad match
**athena health training modules**	Broad match
**what is ehs in healthcare**	Broad match
**prac fusion**	Broad match
**how to use epic ehr system**	Phrase match (close variant)
**open source healthcare**	Broad match
**pre visit checklist**	Broad match
**urgent care emr**	Broad match
**keeping track of medical history**	Broad match
**next gen medical software**	Phrase match (close variant)
**athena emr pricing**	Broad match
**ehr requirements**	Broad match
**top healthcare software vendors**	Phrase match (close variant)
**electronic patient record**	Broad match
**payer care management**	Broad match
**medical record services**	Broad match
**premier software healthcare**	Phrase match (close variant)
**data mining in healthcare**	Broad match
**cerner ehr reviews**	Broad match
**medical appointment scheduling**	Broad match
**him health information management**	Broad match
**patient tracking spreadsheet template**	Broad match
**healthcare workforce management**	Broad match
**health information systems**	Exact match (close variant)
**health and education**	Broad match
**provider network management**	Broad match
**world health organization on remdesivir**	Broad match
**emr and ehr systems**	Exact match

**health care agent**	Broad match
**free tb test clinic**	Broad match
**hospital system integration**	Broad match
**population health management program**	Broad match
**patient registration**	Broad match
**epic medical program training**	Broad match
**epic documentation for nurses**	Broad match
**health insurance verification software**	Broad match
**tedra health management**	Broad match
**premiermychart**	Broad match
**population health software**	Exact match
**emr records**	Exact match
**epic emr system training**	Phrase match (close variant)
**lean healthcare software**	Broad match
**hospital at home programs**	Broad match
**electronic health records definition**	Exact match
**greenway health**	Broad match
**best healthcare crm**	Broad match
**population health framework**	Broad match
**ehr application analyst iii**	Phrase match (close variant)
**epic documentation**	Broad match
**medical records uams**	Phrase match (close variant)
**patient access registration**	Broad match
**healthcare data blockchain**	Broad match
**emr health care software**	Phrase match
**how to sign up for mychart**	Broad match
**case studies health**	Broad match
**epic healthcare software tutorial**	Phrase match (close variant)
**health care 2020**	Broad match
**sample medical record**	Broad match
**electronic health record mandate**	Exact match
**soarian emr system**	Phrase match (close variant)

**sign in mychart**	Broad match
**therapy practice software**	Broad match
**new patient registration forms pdf**	Broad match
**curent health**	Broad match
**medical records clerk**	Phrase match (close variant)
**medical record app**	Broad match
**moderna**	Broad match
**health software**	Broad match
**misys healthcare software**	Phrase match (close variant)
**computer based patient record**	Broad match
**healthcare testing**	Broad match
**south east care plan management**	Broad match
**best ehr for private practice**	Broad match
**chronic care model**	Broad match
**health risk assessment tool free**	Broad match
**medical practices near me**	Broad match
**who owns the medical records**	Exact match
**population health software vendors**	Broad match
**virtal health**	Broad match
**him manager**	Broad match
**electronic medical records emr system**	Broad match
**sos medical billing solutions**	Broad match
**epiccare ambulatory training**	Broad match
**emr behavioral health**	Phrase match (close variant)
**azd1222**	Broad match
**acurian health studies**	Broad match
**management information system in hospital**	Broad match
**healthcare outreach programs**	Broad match
**hospital supply chain software**	Broad match
**emmi program for patients**	Broad match
**patient health record portal**	Broad match
**medical access patient portal**	Broad match

**tb testing forms**	Broad match
**my health chart login**	Broad match
**digital health provider**	Broad match
**northwell health forms**	Broad match
**bastion omnicell**	Broad match
**epic ehr certification**	Broad match
**medical software program**	Exact match (close variant)
**research studies kansas city**	Broad match
**medical records app for patients**	Broad match
**provider management in healthcare**	Broad match
**sdoh platform**	Broad match
**scm hospital software**	Broad match
**top medical software**	Broad match
**ema electronic medical records**	Broad match
**health information records administration manager**	Phrase match
**roche diabetes care platform**	Broad match
**electronic health medical record**	Exact match
**daily management system healthcare**	Phrase match (close variant)
**chronic disease management companies**	Broad match
**population health managers**	Broad match
**carestream health**	Broad match
**medical records schools**	Phrase match (close variant)
**quarantine issues**	Broad match
**heslth**	Broad match
**ecw eclinicalworks login**	Broad match
**meditech software training online**	Broad match
**eclinicworks**	Broad match
**ehr clinical functions advisor trainer**	Phrase match (close variant)
**electronic health records regulations**	Exact match
**hospital programs**	Broad match
**epic computer system**	Broad match
**hospital spend analytics consultants**	Broad match

**trial studies near me**	Broad match
**health software companies**	Broad match
**clinical data abstraction training**	Broad match
**public healthcare saas companies**	Broad match
**medicalrecord**	Exact match
**e health**	Exact match (close variant)
**pfizer under 5 study**	Broad match
**university hospital medical records**	Broad match
**paid research studies**	Broad match
**healthcare charting**	Broad match
**greenway primesuite**	Broad match
**health careers opportunity program**	Broad match
**healthcare resource utilization questionnaire**	Broad match
**american health tech smart charting**	Broad match
**urgent care emr**	Phrase match (close variant)
**ehr behavioral health**	Broad match
**medical billing software**	Phrase match (close variant)
**medical forms to fill out**	Broad match
**medical studies near me**	Broad match
**uniteus com**	Broad match
**clinic software**	Broad match
**epic emr online training**	Broad match
**benefits of electronic medical records**	Exact match
**health information access specialist records retrieval remote**	Phrase match (close variant)
**learning health system**	Broad match
**view medical records online**	Phrase match (close variant)
**medical records programs**	Exact match (close variant)
**baylor college of medicine vaccine trial**	Broad match
**medical practice software**	Exact match (close variant)
**telemedicen**	Broad match
**medical record review template**	Broad match
**emr citrix**	Phrase match (close variant)

**healthcare qa tester**	Broad match
**cdc studies on natural immunity**	Broad match
**system healthcare**	Broad match
**electronic medical record policy**	Exact match
**access patient visit hospital database templates**	Phrase match (close variant)
**online medical records**	Broad match
**signs that you have anxiety**	Broad match
**healthcare informatics**	Broad match
**pfizer document**	Broad match
**how to use epic systems**	Exact match
**mychart trinity health care**	Broad match
**simple practice emr**	Phrase match (close variant)
**population health initiatives examples**	Broad match
**medicare wellness exam questionnaire form**	Broad match
**netsmart charting**	Broad match
**next gen healthcare**	Broad match
**healthcare risk management program**	Broad match
**eclincalworks**	Broad match
**core emr login**	Broad match
**attentive preventative care management program**	Broad match
**excellian training**	Broad match
**digital health**	Exact match (close variant)
**john hopkins study on antibodies**	Broad match
**health informatics programs**	Broad match
**medical records processor**	Broad match
**clinical information technology**	Broad match
**which training and education programs are vital for health ca**	Broad match
**ehr electronic health records system**	Exact match (close variant)
**health records online free**	Broad match
**what is population health**	Broad match
**what is epic system**	Exact match
**how to use epic ehr system**	Exact match

**vaccine incentive programs**	Broad match
**how old do you have to be to get the covid vaccine**	Broad match
**remote clinical trials**	Broad match
**health information management**	Exact match (close variant)
**swedish mrna vaccine study**	Broad match
**patient charting examples**	Broad match
**health screening online form**	Broad match
**utilization management software vendors**	Broad match
**patient care liaison**	Broad match
**human vaccine trials**	Broad match
**partnering for vaccine equity**	Broad match
**medical referral solutions**	Broad match
**medical device analytics**	Broad match
**best ehr for pain management**	Broad match
**website of hospital management system**	Broad match
**meaningful use requirements**	Broad match
**software development healthcare industry**	Broad match
**need for electronic health records**	Exact match
**health information management technology jobs**	Broad match
**us population health management**	Broad match
**twistle health catalyst**	Broad match
**emr training online free**	Broad match
**macpractice**	Broad match
**princeton hospital**	Broad match
**medical abstraction**	Broad match
**pfizer vaccine fda**	Broad match
**department of health and human services financial program**	Broad match
**salient healthcare**	Broad match
**emr for psychologists**	Phrase match (close variant)
**epic training courses healthcare**	Broad match
**medical lab test report software**	Broad match
**uhdds**	Broad match

**population health data**	Broad match
**care time**	Broad match
**what is better pfizer or moderna**	Broad match
**u of l health my chart**	Broad match
**how do they test for ms**	Broad match
**healthcare payer analytics use cases**	Broad match
**south african study omicron**	Broad match
**electronic medical record implementation**	Exact match
**population health job description**	Broad match
**remdesivir study**	Broad match
**patient education**	Broad match
**cpsi charting system**	Broad match
**emr for mental health**	Broad match
**population health management training**	Broad match
**what is an electronic health record**	Broad match
**population health management platform**	Phrase match
**api healthcare staffing and scheduling**	Broad match
**cloud based ehr systems**	Phrase match (close variant)
**electronic healthcare records**	Exact match
**health information management day in the life**	Broad match
**myhealthrecord com**	Broad match
**lawson software healthcare**	Phrase match (close variant)
**importance of real time decision making in healthcare**	Broad match
**healthsparq**	Broad match
**immunization records**	Broad match
**patient admission assessment**	Broad match
**digital health products**	Broad match
**force field analysis in healthcare**	Broad match
**printable tb test form**	Broad match
**mchart**	Broad match
**medical records database**	Broad match
**check medical records**	Broad match

**allscripts emr**	Phrase match (close variant)
**ehr certified application analyst ii**	Phrase match (close variant)
**pfizer patch**	Broad match
**health care management program**	Exact match (close variant)
**mychart franciscan healthcare**	Broad match
**learn epic healthcare software online tutorials**	Phrase match (close variant)
**medical question website**	Broad match
**swedish vaccine study**	Broad match
**volunteer vaccine trial**	Broad match
**entry level health information management**	Broad match
**health diabetic software**	Broad match
**moderna kidcove study**	Broad match
**myhealthevet**	Broad match
**electronic medical health records**	Exact match
**hospital system example**	Broad match
**patient history form**	Broad match
**healthcare database examples**	Broad match
**best ehr for small practices**	Phrase match (close variant)
**physician assessment**	Broad match
**how do you see the future of the phr**	Phrase match (close variant)
**qms software medical device**	Broad match
**software development healthcare**	Broad match
**pediatrics forms**	Broad match
**clinical laboratory software**	Broad match
**research studies at johns hopkins**	Broad match
**health information technology test**	Broad match
**best medical practice management software**	Phrase match (close variant)
**electronic health records in hospitals**	Exact match
**usf clinical trials**	Broad match
**personal health record software**	Phrase match (close variant)
**free medical billing software**	Broad match
**curemd training videos**	Broad match

**healthcare digital platform**	Exact match (close variant)
**medical records training online**	Broad match
**medical record system**	Broad match
**medicaid management information system**	Broad match
**epic emr pricing**	Broad match
**epic ehr training video 2018**	Broad match
**telemedicine app**	Broad match
**moderna booster study**	Broad match
**doctor appointment software**	Broad match
**relayhealth**	Broad match
**patient record management system documentation**	Broad match
**medical research studies near me**	Broad match
**therapy notes software**	Broad match
**medical record template**	Phrase match
**health care software**	Exact match (close variant)
**eclinical tutorial**	Broad match
**health care referrals**	Broad match
**teva patient assistance program**	Broad match
**electronic patient records**	Exact match
**medical website design**	Broad match
**patient engagement software**	Broad match
**chronic care management software pricing**	Phrase match (close variant)
**healthcare catalyst**	Broad match
**healthcare consultants**	Broad match
**what does population health mean**	Broad match
**neurology ehr**	Phrase match (close variant)
**vasafe**	Broad match
**remote clinical data abstractor**	Broad match
**population health organizations**	Broad match
**home health emr systems**	Broad match
**patientinfo**	Broad match
**electronic health records and nursing**	Exact match

**5 different types of software used in healthcare industry**	Broad match
**doctor scheduling software**	Broad match
**medical records online courses**	Exact match
**emr software free**	Phrase match (close variant)
**medical records software**	Exact match
**emr analyst**	Phrase match (close variant)
**printable medical history forms free**	Broad match
**doctor software free**	Broad match
**medication management applications**	Broad match
**how to get my medical records online free**	Broad match
**online healthcare certificate programs**	Broad match
**healthcare saas company**	Broad match
**api vaccine**	Broad match
**data management healthcare**	Broad match
**lab certification programs**	Broad match
**iclinic health**	Broad match
**oacis**	Broad match
**when will hsv 2 vaccine be available**	Broad match
**optumone**	Broad match
**physical therapy software programs**	Broad match
**pfizer smile**	Broad match
**medical appointment scheduling software**	Broad match
**phreesia**	Broad match
**check hospital admissions**	Broad match
**chronic disease management programs**	Broad match
**cancer vaccines clinical trials**	Phrase match (close variant)
**allscripts gateway**	Broad match
**health records course requirements**	Phrase match (close variant)
**digital health tool**	Broad match
**patient records software**	Phrase match (close variant)
**best pharmacology app for medical students**	Broad match
**patient check out**	Broad match

**webpt**	Broad match
**discovery trial**	Broad match
**clinical data companies**	Broad match
**trinetx**	Broad match
**free ehr for mental health**	Broad match
**practice management software healthcare**	Exact match (close variant)
**protect health information**	Broad match
**clinic management software**	Phrase match (close variant)
**care coordination companies**	Broad match
**online patient registration form**	Broad match
**template for medical case study**	Broad match
**paid trial mrna 1273**	Broad match
**hospital supply chain management software**	Broad match
**electronic health record ehr system**	Exact match (close variant)
**medical record filing system**	Phrase match (close variant)
**therapy emr software**	Broad match
**clinical documentation improvement**	Broad match
**home health charting systems**	Broad match
**kareo emr reviews**	Broad match
**medical software applications**	Exact match (close variant)
**optimum healthcare it**	Broad match
**medical laboratory management system**	Broad match
**medical patient scenarios**	Phrase match (close variant)
**population health management job description**	Broad match
**free clinic for tb test**	Broad match
**medical records coding manager**	Phrase match (close variant)
**medical record request form**	Broad match
**emt trauma assessment scenarios**	Broad match
**health maintenance system**	Broad match
**patient flow manager**	Broad match
**effective population health management can help lead to wh**	Broad match
**standards for ehr**	Exact match

**medical online learning management system**	Broad match
**cerner millennium emr**	Phrase match (close variant)
**population health management tools**	Broad match
**health and specialty care system**	Broad match
**novavax approval in usa**	Broad match
**forms for new patient**	Broad match
**patient appointment**	Broad match
**health records and information technology**	Broad match
**behavioral health ehr systems**	Broad match
**digital medical records**	Broad match
**online medical records**	Exact match
**how to test immune system**	Exact match (close variant)
**pfizer en 6201**	Broad match
**free tuberculosis test near me**	Broad match
**why should health care be free**	Broad match
**oxford trial**	Broad match
**epic ehr help**	Exact match
**medgen**	Broad match
**epic emr system**	Exact match
**electronic medical records regulations**	Exact match
**wellstar mychart**	Broad match
**nextgen training videos**	Broad match
**provider enrollment software**	Broad match
**uh population health**	Broad match
**rush university health systems management**	Broad match
**epic healthcare**	Exact match
**big data in healthcare**	Broad match
**health care management plan**	Broad match
**how to use epic hospital software**	Exact match
**population health jobs remote**	Broad match
**sample medical chart**	Phrase match (close variant)
**electronic health records law**	Exact match

**online health records**	Broad match
**healthcare referral management**	Broad match
**allscripts sunrise training**	Broad match
**syntellis healthcare**	Broad match
**non self administered pcr test**	Broad match
**digital health research**	Broad match
**become epic certified**	Broad match
**healthy studies near me**	Broad match
**epic emr systems**	Phrase match (close variant)
**healthcare apps for patients**	Broad match
**pfizer papers**	Broad match
**phizer study**	Broad match
**outpatient software**	Broad match
**find my medical records**	Broad match
**chirofusion reviews**	Broad match
**founder of epic emr**	Exact match
**population health care**	Broad match
**health information management technology**	Broad match
**medical questions webmd**	Broad match
**castlight vera health**	Broad match
**emr training program**	Exact match (close variant)
**medical management software**	Exact match (close variant)
**john hopkins study**	Broad match
**patient management software**	Broad match
**greenway practice management**	Broad match
**oak street health population health director corporate leader**	Broad match
**cbc test for immune system**	Phrase match (close variant)
**healthcare credentialing software**	Phrase match (close variant)
**population health management program**	Exact match (close variant)
**solidarity trial**	Broad match
**medical information technology**	Broad match
**healthcare learning management system**	Broad match

**unite us health**	Broad match
**mayo patient records**	Phrase match (close variant)
**pcr test in bergen county nj**	Broad match
**tb screening test**	Exact match (close variant)
**followmyhealth noreply followmyhealth com**	Broad match
**tb test for employment**	Broad match
**healthcare**	Broad match
**physician**	Broad match
**advancedmd training videos**	Broad match
**networked medical devices**	Broad match
**remote patient monitoring companies**	Broad match
**in health record systems**	Broad match
**p3 healthcare management**	Broad match
**greenway emr training**	Broad match
**patient registration portal**	Broad match
**medical data analytics company**	Broad match
**uab medicine learning system healthstream**	Broad match
**raintree emr tutorial**	Broad match
**5 segments of population health status**	Broad match
**mychart baptist health mychart**	Broad match
**clinical laboratory information systems**	Broad match
**kaiser complex care program**	Broad match
**healthcare reputation management**	Broad match
**free medical coding software**	Phrase match (close variant)
**where can i get an antibody or a diagnostic test for covid 19**	Broad match
**athena billing services**	Broad match
**vaccine study**	Exact match (close variant)
**care program**	Broad match
**medical billing saas**	Broad match
**healthcare management consulting firms**	Broad match
**medical records request form**	Broad match
**performance management in healthcare**	Broad match

**eaglesoft**	Broad match
**patient health information records coordinator**	Broad match
**digital health data**	Broad match
**computer software used in hospitals**	Broad match
**medical practice management software**	Exact match (close variant)
**nurse navigator program**	Broad match
**point of care program**	Broad match
**what is coding in healthcare**	Broad match
**medical billing software programs**	Phrase match (close variant)
**medical records conversion**	Broad match
**aerial health**	Broad match
**e healthcare management system project**	Broad match
**online doctor free**	Broad match
**meditech magic**	Broad match
**medical info on**	Exact match (close variant)
**john hopkins stidy**	Broad match
**johns hopkins population health management certificate**	Broad match
**platforms for telemedicine**	Broad match
**free online health assessment test**	Broad match
**medical health records software**	Exact match
**health innovation**	Broad match
**urgent care emr**	Exact match
**digital health pass partners**	Broad match
**react study**	Broad match
**patients scheduling system**	Broad match
**health center manager**	Broad match
**compare ehr systems**	Exact match
**hospital patient tracking systems**	Broad match
**medical necessity example**	Broad match
**patient check in app**	Broad match
**ehr benefits and challenges**	Broad match
**epic mychart patient portal account**	Broad match

**drchrono**	Broad match
**nash research study**	Broad match
**intake healthcare**	Broad match
**myhealth login**	Broad match
**cloudclinic**	Broad match
**epic healthcare application**	Broad match
**unified electronic health record**	Exact match
**pfizer sweden**	Broad match
**ehr for acupuncture**	Broad match
**johns hopkins online medical information**	Phrase match (close variant)
**epic hospital system tutorial**	Broad match
**cna charting point click care**	Broad match
**how to do medical records**	Broad match
**study during pandemic**	Broad match
**moderna clinical trial**	Broad match
**comprehensive care program**	Broad match
**student health forms**	Broad match
**covenant mychart**	Broad match
**coverscan study**	Broad match
**at home clinical trials**	Broad match
**patient lookup**	Broad match
**best home care software**	Broad match
**vaccine placebo**	Broad match
**ahrq programs**	Broad match
**health care information systems a practical approach for hea**	Broad match
**3 different formats for creating and maintaining a personal h**	Phrase match (close variant)
**medical software programs**	Broad match
**ccda healthcare**	Broad match
**health information**	Broad match
**medical screening forms**	Broad match
**medical appointment scheduling software for vaccination**	Broad match
**patient management system**	Broad match

**ehr healthcare**	Broad match
**medical records classes online**	Exact match
**ehr specialist**	Broad match
**why is the system selection process vital to the use and impl**	Broad match
**director health information management**	Broad match
**healthcare softwares**	Exact match (close variant)
**clinical software**	Phrase match (close variant)
**impact of ehr in healthcare**	Exact match
**digital health records**	Broad match
**john hopkins stufy**	Broad match
**eletronic health records**	Exact match
**diabetic care diabetes management software**	Broad match
**safe patient handling program**	Broad match
**patient management pdf**	Broad match
**cerner tutorial for nurses**	Broad match
**medical administrative software**	Broad match
**medical decision making**	Broad match
**scriptsure**	Broad match
**healthcare edi analyst sr**	Broad match
**narrative medicine**	Broad match
**nih booster study**	Broad match
**intergy ehr tutorial**	Phrase match (close variant)
**clinico website**	Broad match
**medical and health information**	Broad match
**free cerner training**	Broad match
**vaccine pfizer**	Broad match
**electronic patient intake forms**	Broad match
**customer service medical records**	Phrase match (close variant)
**i2i health**	Broad match
**medent emr tutorial**	Phrase match (close variant)
**implementing electronic health record system**	Exact match
**electronic health records usa**	Exact match

**electronic medical record standards**	Exact match
**virtual clinical research studies**	Broad match
**epic medical llc**	Exact match
**medical consulting**	Broad match
**online epic training for nurses**	Broad match
**medical records technician schools**	Phrase match (close variant)
**epic ehr systems**	Exact match
**medical case studies examples**	Broad match
**enterprise healthcare**	Broad match
**epic charting tutorial for nurses**	Broad match
**epic ehr overview**	Broad match
**epic emr training free**	Broad match
**urology appointment booking system**	Broad match
**software for home care agency**	Broad match
**medical charting software**	Exact match (close variant)
**test results and electronic health records**	Exact match
**clinical trials virtual**	Broad match
**medical billing system software**	Exact match (close variant)
**patient records software**	Broad match
**hospital billing system**	Broad match
**emr behavioral health**	Broad match
**nextgen healthcare information systems**	Broad match
**home healthcare software price**	Phrase match (close variant)
**what is digital health record**	Exact match
**healthcare it companies in usa**	Broad match
**vaccine production process**	Broad match
**raintree training**	Broad match
**electronic health record statistics**	Exact match
**emr**	Exact match (close variant)
**health care**	Broad match
**meaningful use in healthcare**	Exact match
**revel healthcare**	Broad match

**duke health information management**	Broad match
**population health vs public health**	Broad match
**medical records certification programs**	Broad match
**emr prep test**	Phrase match (close variant)
**houston methodist health portal**	Broad match
**healthcare software companies in usa**	Broad match
**health information management training programs**	Broad match
**all about epic**	Exact match
**healthcare innovation program**	Broad match
**health pop**	Broad match
**cleveland clinic mychart sign up for mychart**	Broad match
**how to get healthcare experience**	Broad match
**healthcare management software companies**	Exact match (close variant)
**moderna booster studies**	Broad match
**acumen emr**	Phrase match (close variant)
**care management health group**	Broad match
**madera side effects**	Broad match
**novavax and fda**	Broad match
**ehr medical records**	Exact match
**hallmark health care solutions inc**	Broad match
**via telemedicine**	Broad match
**pregnancy care management program**	Broad match
**adventhealth medical records portal**	Phrase match (close variant)
**multiple symptom checker for child**	Broad match
**tb blood test**	Exact match (close variant)
**eclinical training videos**	Broad match
**patient assessment forms**	Broad match
**demo electronic health record**	Broad match
**health care admission**	Broad match
**basic medical information everyone should know**	Phrase match (close variant)
**digital health us**	Broad match
**medical records 2021**	Phrase match (close variant)

**medical records classes online**	Broad match
**crm for medical practice**	Broad match
**new pfizer study**	Broad match
**http chpepiceweb health partners org mychart**	Broad match
**raintree software training**	Broad match
**health information management system**	Broad match
**free hospital bed management software**	Broad match
**athena training videos**	Broad match
**top healthcare financial management software united states**	Phrase match (close variant)
**fp in healthcare**	Broad match
**health information technology courses**	Broad match
**data warehousing healthcare**	Broad match
**eclinicalworks certification training**	Broad match
**electronic medical records controversy**	Exact match
**i patient care**	Broad match
**billing software medical**	Exact match (close variant)
**health records course online**	Broad match
**www patientportal com**	Broad match
**health administration program**	Broad match
**medication adherence programs**	Broad match
**what is facets system**	Broad match
**medical manager health systems**	Exact match (close variant)
**electronic patient record system**	Broad match
**pm system healthcare**	Phrase match (close variant)
**medical documentation**	Broad match
**healthcare management duties**	Broad match
**net health software**	Phrase match (close variant)
**19 study**	Exact match (close variant)
**medical patient management software**	Exact match (close variant)
**health billing software**	Broad match
**can you get a test to see if you have had covid 19**	Broad match
**health informatics**	Broad match

**provider led population health management**	Broad match
**remdesivir ebola study**	Broad match
**the importance of electronic health records**	Exact match
**healthcare ehr system training specialist**	Broad match
**free emr**	Phrase match (close variant)
**excelcare health management**	Phrase match (close variant)
**medical app for doctors**	Broad match
**point click care software**	Broad match
**paragon charting for nurses**	Broad match
**cdss clinical decision support system**	Broad match
**hospital information system**	Broad match
**where can i get pre op testing done**	Broad match
**novavax us approval**	Broad match
**healthcare inventory management**	Broad match
**healthcare charting systems**	Broad match
**health care management software companies**	Phrase match (close variant)
**swedish study pfizer**	Broad match
**hms charting system**	Broad match
**patient outreach**	Broad match
**johnson and johnson vaccination cards**	Broad match
**medical patient forms free**	Broad match
**medical software companies list**	Broad match
**medical cares**	Broad match
**health informatics program**	Broad match
**epic emr software**	Exact match
**how to test for natural immunity**	Phrase match (close variant)
**react study org antibody test**	Broad match
**otilimab clinical trials**	Broad match
**qualifacs**	Broad match
**digital health accelerator program**	Broad match
**healthcare hr software**	Phrase match (close variant)
**johns hopkins antibody study**	Broad match

**printable patient education handouts**	Broad match
**cleveland clinic mychart**	Broad match
**your mychart account**	Broad match
**therapy ehr systems**	Phrase match (close variant)
**smart hospital management system**	Phrase match (close variant)
**home health aide certificate programs**	Broad match
**emr services**	Phrase match (close variant)
**covidcenter**	Broad match
**learning epic medical systems**	Broad match
**e health care management system**	Phrase match (close variant)
**health screening programs**	Broad match
**intergy**	Broad match
**certified ehr trainer**	Phrase match (close variant)
**medical scheduling software**	Phrase match (close variant)
**ehr application**	Phrase match (close variant)
**pediatric patient scheduling software**	Broad match
**clinical decision support companies**	Broad match
**patient portals in healthcare**	Broad match
**caregiver management software**	Broad match
**tertiary care center near me**	Broad match
**healthcare hcm**	Broad match
**health management examples**	Broad match
**ehr electronic health records**	Exact match
**medisoft medical billing software**	Phrase match (close variant)
**tcs hospital information system**	Broad match
**ob gyn ehr**	Phrase match (close variant)
**view medical records online free**	Broad match
**emr physical therapy**	Phrase match (close variant)
**patient led research**	Broad match
**how to get medical records**	Broad match
**medicare wellness exam questions**	Broad match
**medline**	Broad match

**epic emr online training**	Phrase match (close variant)
**what is epic in the medical field**	Exact match
**printable triage forms**	Broad match
**referral management software**	Broad match
**human health program**	Broad match
**agile methodology in healthcare**	Broad match
**cdpas program**	Broad match
**phizer studies**	Exact match (close variant)
**lab appointment scheduling system**	Broad match
**cerner health software**	Phrase match (close variant)
**what is meant by population health**	Broad match
**moderna research study**	Broad match
**strata healthcare software**	Phrase match (close variant)
**browardhealth org pages patient portal**	Broad match
**compare and contrast paper based versus electronic medica**	Broad match
**healthcare supply chain consulting**	Broad match
**epic emr certification course**	Broad match
**patient training**	Broad match
**types of electronic medical records systems**	Exact match
**medical health records**	Exact match (close variant)
**healow open access**	Broad match
**hospital management information**	Broad match
**syntellis health**	Broad match
**personal health information**	Broad match
**emergency medical responder certification course**	Exact match (close variant)
**cphims study guide**	Broad match
**health information handler**	Broad match
**medical lab program**	Broad match
**electronic health record definition**	Exact match
**phizer papers**	Broad match
**health data entry**	Broad match
**health and information management**	Broad match

**patient registration system software**	Broad match
**optum hospital and healthcare**	Broad match
**research in healthcare management**	Broad match
**careradius**	Broad match
**free meditech training online**	Broad match
**health management**	Broad match
**clinical research**	Broad match
**health insurance options**	Broad match
**john hopkins vaccine study**	Broad match
**virtual doctor symptom checker**	Broad match
**population health platform**	Exact match (close variant)
**medical screening exam form**	Broad match
**patient education website**	Broad match
**xifin**	Broad match
**pfizer clinical trials**	Broad match
**ema emr training**	Broad match
**emr systems for home health**	Phrase match (close variant)
**clinic management system**	Broad match
**care management companies near me**	Broad match
**covide pcr test**	Broad match
**electronic health records tutorial**	Broad match
**patient care report sample**	Broad match
**laboratory reporting software**	Broad match
**health information schools**	Broad match
**best medical billing software for home based business**	Broad match
**how to get into healthcare technology**	Broad match
**online clinical trials paid**	Broad match
**health management systems**	Broad match
**psychotherapy software**	Broad match
**electronic health records ehr system**	Exact match (close variant)
**doxy me telemedicine app**	Broad match
**free electronic medical records software download**	Phrase match (close variant)

**what is the difference between emr and practice manageme**	Phrase match (close variant)
**my unity netsmart**	Broad match
**population health vendors**	Broad match
**phreesia check in system**	Broad match
**digital vaccine card**	Broad match
**patient data form**	Broad match
**health it programs**	Broad match
**patient tracking systems**	Broad match
**home health care training programs**	Broad match
**healthcare management job requirements**	Broad match
**my health one registration**	Broad match
**patient navigator**	Broad match
**mychart download test results**	Broad match
**myhealthone account**	Broad match
**nursing documentation tools**	Broad match
**gilead remdesivir clinical trials**	Broad match
**free medical database software**	Broad match
**emr charting**	Exact match (close variant)
**top ehr systems 2020**	Broad match
**health information and management systems society**	Broad match
**https mychart trinity health org mychart**	Broad match
**mychart medical records**	Broad match
**population health division**	Broad match
**medical training software**	Broad match
**epic system for healthcare**	Exact match
**help stop the spread**	Broad match
**emr prescription software**	Phrase match (close variant)
**epic systems medical software**	Phrase match (close variant)
**manager population health**	Broad match
**healthcare audit software**	Broad match
**compulink emr**	Broad match
**pfizer clinical trial data release**	Broad match

**health management resource program**	Broad match
**vaccines a measured**	Broad match
**medical billing software free**	Broad match
**health care domain testing**	Broad match
**rhit medical coding**	Broad match
**medical patient intake form**	Phrase match (close variant)
**duke mychart account log in nc**	Broad match
**enli care manager**	Broad match
**emr electronic medical record**	Exact match
**nursing care plan software**	Broad match
**coronavit study**	Exact match (close variant)
**what is electronic health records**	Broad match
**pediatric new patient forms**	Broad match
**healthcare software programs**	Exact match (close variant)
**my mercy health portal**	Broad match
**health**	Broad match
**health engine**	Broad match
**my medical**	Broad match
**free home care software**	Broad match
**mental health therapy software**	Broad match
**best medical billing software**	Phrase match (close variant)
**healthcare grc software**	Phrase match (close variant)
**ucm digital health**	Phrase match (close variant)
**where to get a health screening**	Broad match
**autoimmune screening quiz**	Broad match
**medical his**	Broad match
**free online clinic management system**	Broad match
**emr for psychiatrists**	Phrase match (close variant)
**echo health inc provider portal**	Broad match
**epic hospital software training**	Phrase match
**medical apps for patients**	Broad match
**medical record systems**	Exact match (close variant)

**medical claims review manager**	Broad match
**ehr website**	Broad match
**cerner powerchart training**	Broad match
**medical patient forms**	Broad match
**patient charting systems**	Broad match
**patient access specialist**	Broad match
**allscripts demo**	Broad match
**best patient portal**	Broad match
**pfeiser**	Broad match
**diabetes population health management**	Broad match
**healthcare data management system**	Phrase match (close variant)
**athena health emr tutorial**	Broad match
**health tech**	Broad match
**harris health system houston tx**	Broad match
**aht nursing home software**	Broad match
**moderna vs pfizer**	Broad match
**medical billing**	Broad match
**patient data sheet**	Broad match
**health information system challenges**	Broad match
**free personal health record software**	Phrase match (close variant)
**hms health management systems**	Broad match
**epic emr training videos**	Phrase match (close variant)
**epic charting system for nurses**	Phrase match (close variant)
**population health initiative examples**	Broad match
**tuberculosis tb test**	Exact match (close variant)
**paragon charting system**	Broad match
**fda meeting on pfizer 5 11**	Broad match
**ob gyn software**	Broad match
**setting up a patient portal**	Broad match
**emr charting systems**	Broad match
**care management program**	Broad match
**healthcare forms**	Broad match

**free epic training for nurses**	Broad match
**health care information**	Broad match
**covid19study**	Exact match (close variant)
**healthcare emr systems**	Exact match
**is health information management a good career**	Broad match
**ehospital systems**	Broad match
**patient care coordinator**	Broad match
**why was ehr created**	Exact match
**low cost emr**	Phrase match (close variant)
**c19study com**	Broad match
**free electronic medical records classes**	Broad match
**hospital patient management system**	Broad match
**electronic health records challenges**	Exact match
**mso healthcare**	Broad match
**anthem care management platform**	Phrase match (close variant)
**ehr user support specialist**	Phrase match (close variant)
**epic medical charting training**	Broad match
**two step tb test form**	Broad match
**myuhcare personal health record**	Phrase match (close variant)
**healthcare prm**	Broad match
**therapist billing software**	Broad match
**medical information specialists**	Broad match
**healthcare data standards**	Broad match
**mmis healthcare**	Broad match
**point click care training videos**	Broad match
**health screening**	Broad match
**health first portal**	Broad match
**medical record certification form**	Phrase match (close variant)
**molnupiravir clinical trial**	Broad match
**free medical records request form**	Broad match
**web based hospital management system**	Phrase match (close variant)
**clinical trials in denver**	Broad match

**patient care assistance program**	Broad match
**emr mental health**	Broad match
**ehr for private practice**	Broad match
**free printable medical records forms**	Phrase match (close variant)
**hr software for healthcare canada**	Broad match
**your medical records**	Broad match
**pfizer clinical trial document**	Broad match
**point click care patient care**	Broad match
**care management system software**	Exact match (close variant)
**johns hopkins stufy**	Broad match
**counsol ehr**	Broad match
**population health data analytics**	Broad match
**population health platforms**	Broad match
**epic electronic medical record tutorial**	Broad match
**c19vaccine ri org**	Broad match
**prism healthcare partners**	Broad match
**learning epic emr**	Broad match
**data management in healthcare**	Broad match
**zelenko protocol**	Broad match
**health it companies**	Broad match
**population health conference**	Broad match
**nextgen emr training**	Phrase match (close variant)
**patients charts**	Broad match
**apps for medical records**	Broad match
**credentialing system**	Broad match
**medical software for doctors**	Broad match
**medical paper charts**	Phrase match (close variant)
**health information management systems**	Phrase match (close variant)
**clinic managment**	Broad match
**hospital patient database**	Broad match
**wtc health program**	Broad match
**mychart atlantic health system**	Broad match

**medical diagnostic software**	Phrase match (close variant)
**electronic medical record system examples**	Exact match
**my ehr**	Broad match
**health information technology companies**	Broad match
**using epic in healthcare**	Exact match
**orthopedic new patient forms**	Broad match
**moderna vaccine study**	Broad match
**patient education handouts**	Broad match
**midwestern health management**	Broad match
**director of health information services**	Broad match
**well360 virtual health**	Broad match
**epic healthcare software training online**	Phrase match (close variant)
**patient education system**	Broad match
**pra studies lenexa**	Broad match
**people soft baptist**	Broad match
**online patient registration software**	Broad match
**ehr for mental health**	Broad match
**patient portal system**	Broad match
**crm for medical sales**	Broad match
**epic emr training**	Broad match
**hospital management system overview**	Broad match
**medical forums**	Broad match
**utilization management software**	Broad match
**hospital erp software**	Broad match
**how to use mychart**	Broad match
**eclinicalworks training front desk**	Broad match
**care access group**	Broad match
**epic population health module**	Broad match
**netsmart evolv**	Broad match
**management of healthcare organizations free pdf**	Broad match
**certified electronic health records specialist exam**	Phrase match (close variant)
**best emr for urgent care**	Phrase match (close variant)

**community health outreach program**	Broad match
**wellsky competitors**	Broad match
**health evaluation program**	Broad match
**best medical billing software**	Broad match
**conviva health portal login**	Broad match
**free patient registration forms**	Broad match
**health data management**	Broad match
**hospital billing software**	Broad match
**electronic medical record journal**	Exact match
**how to do medical billing**	Broad match
**healthcare iot**	Broad match
**population health challenges**	Broad match
**therapy emr**	Phrase match (close variant)
**emr systems training free**	Phrase match (close variant)
**md health connection log in**	Broad match
**electronic health record systems**	Exact match (close variant)
**community health**	Broad match
**lab results reading**	Broad match
**my mychart login**	Broad match
**him program online**	Broad match
**ema emr system**	Phrase match (close variant)
**cerner electronic health record**	Broad match
**college of population health**	Broad match
**healthcare information and management systems society**	Broad match
**medical record management**	Broad match
**mychart trinity**	Broad match
**how to use epic charting for nurses**	Phrase match (close variant)
**affordable ehr**	Broad match
**population health tools**	Broad match
**examples of healthcare informatics**	Broad match
**free ppd test**	Broad match
**health assessment questionnaire for adults**	Broad match

**what are hospital information systems**	Broad match
**cerner healthcare software**	Phrase match (close variant)
**medical diagnosis software**	Phrase match (close variant)
**pacs medical software**	Phrase match (close variant)
**net health systems jobs**	Broad match
**free medical coding course online**	Broad match
**titer testing near me**	Phrase match (close variant)
**why are electronic health records important**	Exact match
**ob gyn emr software**	Phrase match (close variant)
**ecw medical program**	Broad match
**hospital scheduling systems**	Broad match
**my ehealth sign in**	Phrase match (close variant)
**social work software**	Broad match
**comprehensive health assessment**	Broad match
**health care program**	Broad match
**electronic health and medical records**	Exact match (close variant)
**mychart**	Broad match
**health portal**	Broad match
**top healthcare analytics companies**	Broad match
**learn epic emr**	Broad match
**ehr records**	Exact match
**new patient paperwork**	Broad match
**digital health issues**	Broad match
**clinical studies overland park**	Broad match
**what hospitals use epic systems**	Exact match
**swedish study pfizer vaccine**	Broad match
**medical screening questionnaire**	Broad match
**how much does health information technology make**	Broad match
**health information records director**	Broad match
**epic user web**	Broad match
**john hopkins lockdown study**	Broad match
**epic software healthcare**	Exact match

**where to get the vaccine near me**	Broad match
**how long does it take for the body to produce antibodies aga**	Broad match
**onpatient**	Broad match
**new patient questionnaire**	Broad match
**clinical systems**	Broad match
**complex care management program**	Broad match
**nextgen healthcare jobs**	Broad match
**surescripts log in**	Broad match
**advanced hospital management system**	Phrase match (close variant)
**american institute of health technology**	Broad match
**patient pre screening questionnaire**	Broad match
**free federal dental grants**	Broad match
**blockchain health records**	Broad match
**ehr software vendors**	Broad match
**value based care program**	Broad match
**kareo ehr reviews**	Phrase match (close variant)
**best electronic medical records**	Exact match
**population health management companies**	Broad match
**meditech charting tips**	Broad match
**test results and electronic health records**	Phrase match (close variant)
**how do electronic health records improve patient care**	Exact match
**family health records**	Broad match
**what is the remdesivir treatment**	Broad match
**free training for healthcare workers**	Broad match
**vaccine administration technique**	Broad match
**medical diagnostic software**	Broad match
**portal health**	Broad match
**physical therapy software**	Broad match
**healthcare electronic medical records**	Exact match
**home care management software**	Phrase match (close variant)
**patient registration forms**	Broad match
**benefits of ehr for patients**	Exact match

**scheduling systems in healthcare**	Broad match
**patient tracking software**	Broad match
**tb test**	Exact match (close variant)
**emr healthcare software**	Exact match (close variant)
**openemr**	Broad match
**health information management consultant**	Broad match
**billing software medical**	Phrase match (close variant)
**ppd screening test**	Broad match
**patients information**	Broad match
**health chart**	Broad match
**facets healthcare software**	Phrase match (close variant)
**azd7442**	Broad match
**nexgen computer program**	Broad match
**care management programs**	Broad match
**patient education tools**	Broad match
**roxbury institute for medical management**	Broad match
**what is healthcare data analytics**	Broad match
**employee health management systems**	Broad match
**healthcare software sales**	Broad match
**epic medical records company**	Exact match
**clinical practice management**	Broad match
**health care management programs**	Exact match (close variant)
**nexgen medical systems**	Broad match
**medical library software**	Broad match
**emr programs near me**	Exact match
**cerner health provider portal**	Broad match
**healthcare contract lifecycle management united states**	Broad match
**pfizer vaccine michigan**	Broad match
**hipaa compliant crm**	Broad match
**pfizer side effects released 2022 9 pages**	Broad match
**sigma care emr**	Broad match
**free medical form templates**	Broad match

**population health platforms**	Exact match (close variant)
**harris health learning management system**	Broad match
**health companion**	Broad match
**sap healthcare**	Broad match
**cerner millennium laboratory information system**	Broad match
**eclinicals**	Broad match
**ehr training**	Broad match
**cantata health llc**	Broad match
**transition from paper records to electronic health records**	Exact match
**electronic healthcare services platform**	Broad match
**healthcare information technology**	Broad match
**community health and dental patient portal**	Broad match
**clinical trials pfizer**	Broad match
**epic emr help**	Exact match
**epic charting system training**	Broad match
**idx medical software tutorial**	Phrase match (close variant)
**emt programs near me**	Broad match
**patient portal emr**	Broad match
**best patient portals**	Broad match
**ehr applications**	Exact match (close variant)
**electronic health record policy**	Exact match
**patient portal**	Broad match
**medical screening exam template**	Broad match
**eclinicalworks patient portal**	Broad match
**dataq health**	Broad match
**compulink**	Broad match
**best databases for medical research**	Broad match
**healthcare technology platforms**	Broad match
**health information websites**	Phrase match (close variant)
**free medical program**	Broad match
**healthcare case management software**	Broad match
**vaccine records**	Broad match

**medical forms pdf**	Broad match
**patient engagement solutions**	Broad match
**healthcare case study**	Broad match
**nextgen practice management**	Broad match
**medical history software**	Broad match
**population health examples**	Broad match
**smartcare**	Broad match
**trucare**	Broad match
**health care numbers**	Broad match
**health passport**	Broad match
**getting a physical exam**	Broad match
**introduction to healthcare financial management**	Broad match
**log into mychart account**	Broad match
**health care information systems**	Broad match
**top medical billing software**	Phrase match (close variant)
**healthcare revenue cycle management software**	Phrase match (close variant)
**conduent care solutions**	Broad match
**medical practice management software uses**	Phrase match (close variant)
**phmc programs**	Broad match
**healthcare it companies**	Broad match
**patient care training programs**	Broad match
**student health management system**	Phrase match (close variant)
**nextgen population health**	Broad match
**benefits of emr for patients**	Broad match
**electronic health records specialist**	Broad match
**what are electronic health records**	Exact match
**future of population health**	Broad match
**electronic health records improve quality of care**	Exact match
**digital healthcare products**	Broad match
**medical charge capture**	Broad match
**mychart premier health partners**	Broad match
**practice management system**	Broad match

**request healthcare records**	Broad match
**smart hospital software**	Broad match
**updox patient portal sign in**	Broad match
**my health record registration**	Broad match
**electronic health systems**	Exact match
**patient tracking software free**	Broad match
**population health management strategies tools applications**	Broad match
**athena healtg**	Broad match
**caretracker**	Broad match
**healthcare domain knowledge**	Broad match
**healthcare management software**	Exact match
**medicare wellness visit questionnaire**	Broad match
**medical management program**	Exact match (close variant)
**pms patient management system**	Broad match
**oracle health management system**	Phrase match (close variant)
**ehr demos**	Broad match
**reengineering of health care**	Broad match
**medical study prep app**	Broad match
**healthcare population health**	Broad match
**how long should you wait to get booster after having covid**	Broad match
**open dcm medical files**	Broad match
**epic health records**	Exact match
**epic healthcare training**	Broad match
**healthcare payer platforms**	Broad match
**clinical practice management software**	Phrase match (close variant)
**american healthcare technologies**	Broad match
**clinical decision unit**	Broad match
**ehr systems benefits**	Exact match
**how do i get a patient portal**	Broad match
**emr system training free**	Broad match
**sweden vaccine study**	Broad match
**snapmd**	Broad match

**care management solutions**	Broad match
**enterprise health systems**	Broad match
**c19study**	Exact match (close variant)
**learning electronic medical records**	Exact match
**hospital training programs**	Broad match
**allscripts course**	Broad match
**ehr certified application analyst iii**	Broad match
**population health management software**	Exact match
**health data tracking**	Broad match
**personal health records**	Exact match (close variant)
**sap healthcare module**	Broad match
**best rated emr**	Phrase match (close variant)
**medical records free**	Broad match
**medical records medical records**	Broad match
**idx medical software**	Phrase match (close variant)
**health training programs**	Broad match
**software for medical offices**	Broad match
**best home healthcare software**	Phrase match (close variant)
**patient portals for healthcare**	Broad match
**vaccine control**	Broad match
**medical software providers**	Broad match
**online medical record software**	Exact match
**medical consulting and solutions**	Broad match
**medical information websites**	Phrase match (close variant)
**medical informatics companies**	Broad match
**digital health tools**	Broad match
**personal health record forms**	Phrase match (close variant)
**epic mychart patient portal**	Broad match
**allscripts careport login**	Broad match
**health information records administration supervisor**	Phrase match
**patient needs assessment**	Broad match
**home care policy and procedure template**	Broad match

**what is a healthcare platform**	Broad match
**why is population health important**	Broad match
**doctor referral**	Broad match
**altapoint**	Broad match
**patient advocate training programs**	Broad match
**clinical finding**	Broad match
**digital health product**	Broad match
**emory healthcare portal**	Broad match
**population health strategy examples**	Broad match
**johns hopkins medical information**	Phrase match (close variant)
**hospital management system software**	Exact match (close variant)
**medical records specialist**	Broad match
**how to be a patient**	Broad match
**patient portal service**	Broad match
**medical records central supply clerk**	Phrase match (close variant)
**epic software for hospitals**	Phrase match (close variant)
**patient management app**	Broad match
**epic training courses online**	Broad match
**lightbeam health**	Broad match
**ea health san diego ca**	Broad match
**hospital management systems**	Exact match (close variant)
**chronic care management company**	Broad match
**epion health reviews**	Broad match
**healthcare it solutions**	Broad match
**healthcare vendor management**	Broad match
**my chart kettering health**	Broad match
**emr systems training**	Broad match
**epiccare inpatient**	Broad match
**electronic medical records definition**	Exact match
**health data utility**	Broad match
**free medical billing**	Broad match
**medical assessment test**	Broad match

**clinical study kansas city**	Broad match
**umms learning management system**	Broad match
**epic system certification**	Broad match
**epic scheduling and registration**	Broad match
**allscripts referral management**	Broad match
**health solutions info**	Broad match
**phizer report**	Broad match
**medical intake forms download free**	Broad match
**medical software training**	Broad match
**medical billing software**	Exact match (close variant)
**electronic health records ehr**	Exact match
**metavision**	Broad match
**epic patient access training**	Broad match
**health information records director**	Phrase match
**health management strategies**	Broad match
**emr specialist**	Broad match
**what hospitals use epic emr**	Exact match
**documentation for medical records**	Exact match
**hospital information management system**	Broad match
**lenus health**	Broad match
**healthcare appointment scheduling**	Broad match
**medical questions and answers**	Broad match
**veradigm**	Broad match
**when is the flu vaccination generally available for patients**	Broad match
**doctor clinic software**	Broad match
**healthcare operations research**	Broad match
**therapy documentation software**	Broad match
**health integration partners**	Broad match
**assessment of pain in nursing**	Broad match
**emr systems**	Exact match (close variant)
**clinical studies near me**	Broad match
**open patient**	Broad match

**medical forms online**	Broad match
**pfizer study**	Exact match (close variant)
**enterprise healthcare software**	Phrase match (close variant)
**population health registry**	Broad match
**hospital scheduling software**	Broad match
**free healthcare**	Broad match
**crm clinical**	Broad match
**lab requisition form blood tests**	Broad match
**eclinicalworks tutorial**	Broad match
**pivot health insurance provider portal**	Broad match
**insync healthcare solutions**	Broad match
**health services research**	Broad match
**digital health pass costa rica**	Broad match
**swedish study mrna vaccine**	Broad match
**precyse**	Broad match
**care coordinator case manager**	Broad match
**software medical**	Exact match (close variant)
**laboratory billing software**	Broad match
**clintrac**	Broad match
**redox interoperability**	Broad match
**arcadia healthcare solutions**	Broad match
**healthcare erp system**	Broad match
**austin research studies**	Broad match
**medidata**	Broad match
**electronic medical records analyst**	Phrase match (close variant)
**patient information handouts**	Broad match
**epic medical training**	Broad match
**healthcare supply chain software**	Broad match
**epic electronic health record system**	Broad match
**hiv vaccine trials**	Phrase match (close variant)
**patient flow management system**	Broad match
**care coordination training programs**	Broad match

**simple practice alternatives**	Broad match
**health records**	Exact match
**epic health system**	Broad match
**how to use epic charting system**	Broad match
**ambulatory testing**	Broad match
**ehr implementation checklist**	Phrase match (close variant)
**free therapy notes**	Broad match
**patient portal website**	Broad match
**cavitch acute care and telemedicine**	Broad match
**myhealthrecord**	Broad match
**healthcare billing software**	Phrase match (close variant)
**epic billing system training**	Broad match
**hcs healthcare**	Broad match
**pra clinical trials**	Broad match
**health services program**	Broad match
**top hospital software**	Broad match
**athena emr**	Broad match
**soarian healthcare**	Broad match
**iqvia study**	Broad match
**medical results**	Broad match
**small hospital software**	Broad match
**xsolis**	Broad match
**online lab appointment booking system**	Broad match
**patagonia health**	Broad match
**health care staffing software**	Phrase match (close variant)
**patient assessment form template**	Broad match
**remdesivir trials**	Broad match
**care management definition**	Broad match
**ventilator survival rate**	Broad match
**hospital management software india**	Phrase match (close variant)
**clinical chart auditor**	Broad match
**managed care**	Broad match

**cocid**	Broad match
**swedish pfizer study**	Broad match
**healthcare epic**	Exact match
**phizer cdc**	Broad match
**covaxin study results**	Broad match
**managed care software**	Broad match
**how to use epic emr system**	Phrase match (close variant)
**diagnose me**	Broad match
**health development program**	Broad match
**get my medical records online**	Broad match
**dashboard healthcare**	Broad match
**emr for psychiatrists**	Broad match
**online health program**	Broad match
**conversion to electronic health records**	Exact match
**electronic medical records classes**	Broad match
**epic er**	Broad match
**test results and electronic medical records**	Phrase match
**emr classes**	Exact match (close variant)
**electronic health record purpose**	Exact match
**population health registries**	Broad match
**doctor report example**	Broad match
**health level 7**	Broad match
**static practicefusion com**	Broad match
**epic training for physicians**	Broad match
**iclinic**	Broad match
**benefits of electronic health record systems**	Exact match
**medical software mac**	Phrase match (close variant)
**mental health treatment plan software**	Broad match
**definitive healthcare competitors**	Broad match
**point click care training videos for nurses**	Broad match
**medical data managment**	Broad match
**hospital laboratory management system**	Broad match

**rrh enterprise health**	Broad match
**tims software medical**	Broad match
**free ehr for mental health**	Phrase match (close variant)
**epic ehr software**	Exact match
**patient health record**	Broad match
**logistics and supply chain management in healthcare**	Broad match
**hcue**	Broad match
**electronic health and medical records**	Broad match
**university of md medical system**	Broad match
**b virtual health and wellness**	Broad match
**hospital management software**	Broad match
**health care incentive programs**	Broad match
**ipatientcare**	Broad match
**medical records field technician**	Broad match
**epic medical records**	Exact match
**patient survey questions**	Broad match
**goldcare telemed**	Broad match
**epic hospital software training**	Broad match
**nextmd patient portal enrollment form**	Broad match
**patient advocacy program**	Broad match
**mental health ehr software**	Phrase match (close variant)
**together trial lancet**	Broad match
**medical and health services managers**	Broad match
**eclinicalworks emr**	Phrase match (close variant)
**med assets**	Broad match
**mychart bcm edu**	Broad match
**medical coding automation software**	Phrase match (close variant)
**what is the livongo program**	Broad match
**free home health care software**	Phrase match
**lytec medical software**	Broad match
**healthrecords**	Exact match
**patient medical profile**	Broad match

**understanding medical records**	Phrase match (close variant)
**new patient registration form online**	Broad match
**epic program for hospitals**	Phrase match (close variant)
**american health tech user guide**	Broad match
**free ppd testing**	Broad match
**context media health**	Broad match
**protenus**	Broad match
**introduction to medical records**	Phrase match (close variant)
**virtual nurse**	Broad match
**health information technology programs near me**	Broad match
**electronic medical record programs**	Broad match
**mychart health usf edu mychart**	Broad match
**home health billing software**	Broad match
**prism health lab**	Broad match
**health insurance software solutions**	Broad match
**access medical records online**	Phrase match (close variant)
**borgess my health**	Broad match
**inpatient clinical trials**	Broad match
**what is electronic health records**	Exact match
**practice fusin**	Broad match
**prehospital assessment**	Broad match
**population health management certification**	Broad match
**virtual trials**	Broad match
**free patient history forms**	Broad match
**my personal health record**	Broad match
**how to create patient portal**	Broad match
**human health service program**	Broad match
**john and johnson vaccine**	Broad match
**register for vaccine**	Broad match
**surgery assistance programs**	Broad match
**johns hopkins research studies**	Broad match
**healthcare software vendor**	Broad match

**online nurse case management programs**	Broad match
**vaccination healthcare appointment scheduling system**	Broad match
**best app to track lab results**	Broad match
**what are the major issues in healthcare**	Broad match
**patient hospital**	Broad match
**epic patient station**	Broad match
**e clinical works**	Broad match
**ut austin research studies**	Broad match
**why use electronic health records**	Exact match
**healthcare information**	Broad match
**claims processing system**	Broad match
**personal health record template**	Broad match
**paid clinical trials oklahoma city**	Broad match
**medical authorization form**	Broad match
**electronic medical records systems**	Exact match
**patients education**	Broad match
**integrity login emr**	Broad match
**electronic medical records training online free**	Broad match
**medicare wellness visit patient questionnaire**	Broad match
**my health record patient portal**	Phrase match (close variant)
**professional patient advocate certificate training program**	Broad match
**medical record emr**	Exact match (close variant)
**scanning medical charts**	Broad match
**health stream learning center**	Broad match
**check your medical symptoms**	Phrase match
**best ehr system**	Phrase match (close variant)
**together clinical trial**	Broad match
**health commerce system**	Broad match
**personal health information management**	Broad match
**healthecrm**	Broad match
**end of the pandemic**	Broad match
**digital health pdf**	Broad match

**healthcatalyst com**	Broad match
**health care provider**	Broad match
**colcorona clinical trial**	Broad match
**medical software systems**	Exact match (close variant)
**arcadia pop health**	Broad match
**healthcare strategies**	Broad match
**credible behavioral health login**	Broad match
**healthcare it training and placement**	Broad match
**heath catalyst**	Broad match
**utilization management vendors**	Broad match
**electronic medical records software**	Exact match
**medical brochures for patients**	Broad match
**heath records**	Broad match
**healthcare operating systems**	Broad match
**patient advocate programs**	Broad match
**tuberculosis screening**	Exact match (close variant)
**johns hopkins social distancing study**	Broad match
**epic medical**	Exact match
**digital health blog**	Broad match
**medpace studies**	Broad match
**open ended health questions**	Broad match
**health informatics certificate**	Broad match
**integrated care management program**	Broad match
**occupational health assessment**	Broad match
**useful medical information**	Phrase match (close variant)
**american healthtech ltc login**	Broad match
**home clinical trials done online**	Broad match
**digital health company**	Broad match
**outpatient clinical trials**	Broad match
**medical information**	Exact match (close variant)
**home healthcare software**	Phrase match (close variant)
**cvd trials org**	Broad match

**best electronic health record system**	Exact match
**what are disease management programs**	Broad match
**medical patient forms**	Phrase match (close variant)
**american healthtech portal**	Broad match
**medical records specialist i**	Phrase match (close variant)
**carenotify**	Broad match
**mhealth app for obesity**	Broad match
**optimus emar**	Broad match
**primary care compliance**	Broad match
**population health risk stratification**	Broad match
**how to get hospital records**	Broad match
**valera health**	Broad match
**information about epic**	Exact match
**epic training healthcare**	Broad match
**patient care navigator**	Broad match
**homecare agency software**	Broad match
**hospital software systems**	Exact match (close variant)
**epic software for healthcare**	Exact match
**online patient intake forms**	Broad match
**patient interview guide**	Broad match
**different medical software systems**	Broad match
**best pain management emr**	Broad match
**vaccine health risk management software**	Broad match
**managed care contract modeling**	Broad match
**24lik mychart**	Broad match
**eclipsys**	Broad match
**healthcare and computer science**	Broad match
**functional medicine patient questionnaire**	Broad match
**enterprise health management platform**	Exact match
**how pfizer works in the body**	Broad match
**digital health solutions companies**	Broad match
**human and health service program**	Broad match

**drug database for population health**	Broad match
**point click care skilled nursing log in**	Broad match
**free medical office forms printable**	Broad match
**small practice ehr**	Broad match
**population health management definition**	Broad match
**emr software epic**	Exact match
**healthcare preparedness program**	Broad match
**acurian health**	Broad match
**relimed**	Broad match
**protected health information management tool**	Broad match
**free medical forms online**	Broad match
**data abstraction healthcare**	Broad match
**health login**	Broad match
**health record online**	Broad match
**follow my health website**	Broad match
**point click care skilled nursing charting**	Broad match
**athenahealth emr**	Phrase match (close variant)
**social services software**	Broad match
**addendum medical record**	Broad match
**doctors website**	Broad match
**paid clinical research studies**	Broad match
**nextgen billing**	Broad match
**healtheintent**	Broad match
**patient case management software**	Phrase match (close variant)
**hospital management system**	Phrase match (close variant)
**wound management software**	Broad match
**budgeting in healthcare management**	Broad match
**cerner healthintent**	Broad match
**physician liaison**	Broad match
**lab information system**	Broad match
**change healthcare eft enrollment**	Broad match
**health information management clerk home care**	Broad match

**c 19 studies com**	Phrase match (close variant)
**electronic health record implementation**	Exact match
**independent vaccine study**	Broad match
**best home health care software**	Phrase match
**patient care management**	Broad match
**health information manager**	Broad match
**hsv studies**	Broad match
**healthcare accounting software**	Phrase match (close variant)
**patient app**	Broad match
**digital health supplies**	Broad match
**find medical records**	Broad match
**healthcare assessment form**	Broad match
**healthnote**	Broad match
**athenanet training**	Broad match
**american health tech**	Broad match
**ehealth emr**	Exact match
**paid clinical trials near me**	Broad match
**medical laboratory information system**	Broad match
**pfizer data released**	Broad match
**hospital rounding software**	Broad match
**healthcare delivery systems**	Broad match
**java healthcare projects**	Broad match
**public health software**	Broad match
**medical chart software**	Broad match
**uab medical records request form**	Phrase match (close variant)
**patient web portal**	Broad match
**meditech training for nurses**	Broad match
**emr programs near me**	Broad match
**arcadia solutions**	Broad match
**hospital patient record**	Broad match
**therapynotes**	Broad match
**emr course**	Exact match (close variant)

**arcadia analytics**	Broad match
**health information management system**	Phrase match (close variant)
**medical data abstraction training**	Broad match
**top medical software**	Phrase match (close variant)
**charmhealth**	Broad match
**nhs clinical trials**	Broad match
**epic computer charting system training**	Broad match
**biosoftworld medical scheduler**	Broad match
**improving population health**	Broad match
**cerner hospital software**	Broad match
**average cost of ehr system**	Broad match
**health record**	Exact match
**population health service organization**	Broad match
**medical business**	Broad match
**electronic health records example**	Exact match
**health care it**	Broad match
**eclinicalworks training**	Broad match
**free allscripts training**	Broad match
**patient portal implementation project plan**	Broad match
**emr healthcare software**	Phrase match (close variant)
**emr electronic medical record software**	Broad match
**medical records administrator**	Broad match
**mychart sign up page**	Broad match
**ehr and billing software united states**	Broad match
**health information examples**	Broad match
**pfizer safety data release**	Broad match
**echo health remittance provider portal**	Broad match
**pay for performance programs in healthcare**	Broad match
**pop healthcare providers**	Broad match
**medical clinic forms**	Broad match
**medical documentation app**	Broad match
**care link health**	Broad match

**employee health record form**	Broad match
**epic software for hospitals**	Exact match
**primary care emr**	Broad match
**healthfusion training videos**	Broad match
**top health it companies**	Broad match
**free online healthcare management courses**	Broad match
**get the vaccine**	Broad match
**medical records director**	Broad match
**phreesia inc**	Broad match
**relyhealth**	Broad match
**medical patient registration forms**	Phrase match (close variant)
**electronic health records in healthcare**	Broad match
**healthware**	Broad match
**software for medical offices**	Exact match (close variant)
**epic emr system demo**	Broad match
**population management healthcare**	Broad match
**manager clinic**	Broad match
**raintree systems**	Broad match
**population health certificate**	Broad match
**internet of medical things**	Broad match
**online health care programs**	Broad match
**electronic medical records emr software**	Broad match
**patient engagement solution**	Broad match
**patient portal login**	Broad match
**one healthcare id optum**	Broad match
**school health software**	Broad match
**cerner medical billing software**	Broad match
**ros and physical exam template**	Broad match
**online medical records for patients**	Exact match
**hospital appointment management**	Broad match
**medtracker**	Broad match
**connect care**	Broad match

**epic documentation system**	Broad match
**the lancet study**	Broad match
**how to become an anaplastologist**	Broad match
**patient management problem**	Broad match
**health information careers**	Broad match
**epic emr training manual pdf**	Broad match
**health information management company**	Broad match
**study c4591007**	Broad match
**diagnostic medical online**	Broad match
**medsphere**	Broad match
**doe health screening app**	Broad match
**patient demographics form**	Broad match
**health catalysts**	Broad match
**point of care charting cna**	Broad match
**population management in healthcare**	Broad match
**medical devices development app**	Broad match
**electronic healthcare software**	Exact match
**athenia health**	Broad match
**mental health notes software**	Broad match
**software for medical records**	Exact match
**azalea health**	Broad match
**epic software healthcare training**	Phrase match (close variant)
**patient portal software**	Broad match
**healthcare scenarios**	Broad match
**health ehr**	Broad match
**adracare**	Broad match
**examples of digital health**	Broad match
**health management system project**	Broad match
**patient registration software**	Exact match
**patient system**	Broad match
**medical equipment inventory software**	Broad match
**medical records supervisor**	Phrase match (close variant)

**electronic medical record law**	Exact match
**population analytics**	Broad match
**telehealth studies**	Broad match
**what is jiva software**	Broad match
**my chart mercy medical login**	Broad match
**integrated care**	Broad match
**epic healthcare login**	Exact match
**integrated health record**	Phrase match (close variant)
**crispr and vaccines**	Broad match
**clinical trials in africa**	Broad match
**clinic management**	Broad match
**can u catch omicron twice**	Broad match
**risk assessment template healthcare**	Broad match
**md now ambra health access**	Broad match
**epic system for healthcare training**	Broad match
**ehr developer**	Broad match
**referral forms for doctors**	Broad match
**electronic health book**	Phrase match
**government health care assistance program**	Broad match
**ensemble clinical trial**	Broad match
**patient crm**	Broad match
**patient check in software**	Broad match
**medical report**	Broad match
**seroprevalence studies**	Broad match
**patient health record system**	Broad match
**workflow process in healthcare**	Broad match
**ciox health medical records**	Phrase match (close variant)
**dermatology practice management software**	Broad match
**medical appointment scheduling software programs**	Broad match
**how to work epic system**	Broad match
**saas healthcare software**	Broad match
**online health records**	Exact match

**tb testing**	Exact match (close variant)
**care software for medical**	Exact match (close variant)
**ehr podiatry**	Broad match
**caregiver software**	Broad match
**patient self scheduling**	Broad match
**emr programs near me**	Phrase match (close variant)
**emr trainer**	Phrase match (close variant)
**patient electronic medical records**	Exact match
**iot healthcare**	Broad match
**care coordination software vendors**	Broad match
**baycare online portal**	Broad match
**cerner patient accounting training manual**	Broad match
**do i have to wait to get booster if i had covid**	Broad match
**pcr test in stock**	Broad match
**health assessment form**	Broad match
**insync healthcare solutions revenue**	Broad match
**teledoc software**	Broad match
**coordinated care management**	Broad match
**phreesia software**	Broad match
**bronson hospital my chart**	Broad match
**mckesson emr software**	Broad match
**mental health emr**	Broad match
**medical questionnaire form**	Broad match
**epic emr certification training**	Broad match
**johns hopkins studies**	Broad match
**cerner training**	Broad match
**whats telemed**	Broad match
**care management program description**	Broad match
**health information management pdf**	Broad match
**what is population health definition**	Broad match
**population health in healthcare**	Broad match
**personal health record template pdf**	Phrase match

**nextgen healthcare software**	Phrase match (close variant)
**epic clinical training**	Broad match
**primary care assessment tool questionnaire**	Broad match
**electronic medical records for dummies**	Exact match
**health services description**	Broad match
**learning epic charting**	Broad match
**what is athenahealth patient portal**	Broad match
**medical records online**	Exact match
**phreesa**	Broad match
**medical software free**	Broad match
**health information management companies**	Broad match
**eclinical works**	Broad match
**patient care software**	Exact match
**counsol vs simple practice**	Broad match
**request medical records online**	Broad match
**strategies for population health**	Broad match
**populaton health**	Broad match
**appointment management system in hospital**	Broad match
**dallas research studies**	Broad match
**end of pandemic**	Broad match
**certification programs healthcare**	Broad match
**healthcare referral management software**	Phrase match (close variant)
**epic emr system training**	Broad match
**patient portal healthcare**	Broad match
**does the meaningful use of electronic health records improv**	Exact match
**mychart patient portal account**	Broad match
**health information management him**	Phrase match (close variant)
**i2i healthcare solutions**	Broad match
**patient portal emr**	Phrase match (close variant)
**electronic medical records industry**	Exact match
**learn how to use epic software**	Exact match
**online masters degree programs in healthcare administratio**	Broad match

**epic training for nurses**	Broad match
**home health software**	Phrase match (close variant)
**tuberculosis test**	Exact match (close variant)
**medical evaluation forms**	Broad match
**current health company**	Broad match
**patient test results**	Broad match
**health information management degree**	Broad match
**patient engagement platform**	Broad match
**health system software**	Exact match (close variant)
**health information services director**	Broad match
**harris caretracker patient portal registration**	Broad match
**example of electronic medical records**	Exact match
**primary care emr**	Exact match
**medical history software**	Exact match (close variant)
**computer assisted coding 3m**	Broad match
**tb screening tests**	Exact match (close variant)
**university hospital**	Broad match
**covaxin trials in us**	Broad match
**healthcare domain knowledge for testers**	Broad match
**epic computer system training manual**	Broad match
**home health agency software**	Phrase match (close variant)
**free online personal medical records**	Broad match
**my health history**	Exact match (close variant)
**best urgent care ehr**	Broad match
**health & fitness program consultants baltimore md**	Broad match
**epic patient check in**	Exact match
**paid clinical research studies near me**	Broad match
**examples of population health management**	Broad match
**hospital information system software**	Broad match
**patient record system**	Broad match
**medical manager software training**	Broad match
**https phreesia com**	Broad match

**digital health practice**	Broad match
**comprehensive physical examination**	Broad match
**population health definition**	Broad match
**personal health management app**	Broad match
**medical record system**	Exact match (close variant)
**phn clinics net**	Broad match
**patient portal lab results**	Broad match
**icanotes behavioral health ehr**	Phrase match (close variant)
**healthcare practice management software**	Exact match (close variant)
**patient information form**	Broad match
**doctors forms**	Broad match
**healthcare computer programs**	Broad match
**manager of medical records coding**	Phrase match (close variant)
**epic ehr training**	Broad match
**behavioral health ehr**	Broad match
**how to set up e prescribing**	Broad match
**patient blood management program**	Broad match
**medical records information**	Broad match
**patient managment**	Exact match (close variant)
**medical schedule software**	Phrase match (close variant)
**access to healthcare**	Broad match
**health management information systems book**	Phrase match (close variant)
**health information records supervisor**	Phrase match
**my patient portal**	Broad match
**impact of electronic health records**	Exact match
**medical questions**	Broad match
**united healthcare disease management programs**	Broad match
**electronic health record ehr**	Exact match
**app for health care**	Broad match
**patient health system**	Broad match
**patient record**	Exact match (close variant)
**what is an electronic medical record**	Exact match

**healthcare finance**	Broad match
**hospital business intelligence**	Broad match
**electronic medical records for patients**	Exact match
**tb testing near me free**	Broad match
**medical software list**	Broad match
**eclypsis**	Broad match
**software health management**	Exact match (close variant)
**conviva health portal**	Broad match
**healthcare e learning companies**	Broad match
**optima net health**	Broad match
**carecloud**	Broad match
**research on pandemic**	Broad match
**occupational health software**	Broad match
**elixia clinical research collaborative**	Broad match
**free multiple symptom checker**	Broad match
**kivicare**	Broad match
**top health information technology companies**	Broad match
**emr startups**	Broad match
**disease management program description**	Broad match
**excela health greensburg pa**	Broad match
**medical records coding course**	Phrase match (close variant)
**medical software free**	Phrase match (close variant)
**mychart medical records**	Phrase match (close variant)
**telemedicine**	Broad match
**software used in medical offices**	Broad match
**health information management field**	Broad match
**electronic health records company**	Broad match
**healthcare platforms examples**	Broad match
**test results and electronic health records**	Broad match
**hospital management system**	Broad match
**population health overview**	Broad match
**best population health programs**	Broad match

**medical patient database**	Broad match
**cgm sa**	Broad match
**athenahealth com**	Broad match
**salesforce for healthcare**	Broad match
**health management systems of america**	Broad match
**medical staff credentialing software**	Broad match
**how electronic health records improve quality of care**	Exact match
**medical tourism management software**	Broad match
**pre register patients**	Broad match
**criterions ehr**	Broad match
**what is a medical management program**	Broad match
**hospital medical software**	Broad match
**patient care assessment**	Broad match
**patient portal software cost**	Phrase match (close variant)
**best practice management software**	Broad match
**salesforce healthcare**	Broad match
**doctor appointment scheduling system**	Broad match
**hims software**	Phrase match (close variant)
**patient schedule software**	Broad match
**health screening survey**	Broad match
**health care services**	Broad match
**valcare health management systems inc**	Broad match
**disease management programs for diabetes**	Broad match
**american health tech smart charting login**	Broad match
**medical information online**	Phrase match (close variant)
**electronic medical records electronic health records**	Exact match
**clinic office**	Broad match
**patient chart audit form**	Broad match
**district health information system**	Broad match
**patient appointment software**	Broad match
**nextgen healthcare**	Broad match
**population health management metrics**	Broad match

**epic nursing charting tutorial**	Broad match
**how to get your medical records**	Phrase match (close variant)
**tb testing procedure**	Broad match
**health information technology examples**	Broad match
**health information major**	Broad match
**check in epion health**	Broad match
**patient health care services**	Phrase match (close variant)
**tb test form free**	Broad match
**telehealth app for laptop**	Broad match
**electronic medical records assessment**	Broad match
**hospital database free**	Broad match
**epic emr for dummies**	Exact match
**software for healthcare providers**	Exact match (close variant)
**electronic health records specialist training**	Broad match
**patient health records**	Broad match
**electronic health record case study**	Exact match
**medical billing system software**	Broad match
**care management vendor**	Broad match
**healthcare data analyst**	Broad match
**computerized physician order entry**	Broad match
**care management support**	Broad match
**mental health management system**	Broad match
**general wellness exam**	Broad match
**health care staffing software**	Broad match
**altruista health**	Broad match
**novavax fda**	Broad match
**healthcare cloud computing united states**	Broad match
**care continuity program**	Broad match
**emr lite**	Broad match
**nextgen knowledge base healthwise**	Broad match
**cerner population health management**	Broad match
**emr systems**	Exact match

**medi tech**	Broad match
**my chart medical records**	Broad match
**join vaccine trial**	Broad match
**what is medical records**	Broad match
**health informatics applications**	Broad match
**studies on remdesivir**	Broad match
**vaccine trial information**	Broad match
**healthpro com**	Broad match
**healthcare software implementation jobs**	Broad match
**epic ehr**	Exact match
**epic systems**	Broad match
**epic health software**	Phrase match (close variant)
**remote clinical studies**	Broad match
**heath care**	Broad match
**samples for medical professionals**	Broad match
**personal medical record**	Exact match (close variant)
**healthcare crm software**	Phrase match (close variant)
**mychart questionnaires**	Broad match
**software used in hospitals**	Broad match
**best medical specialities for the future**	Broad match
**cms acute hospital care at home program**	Broad match
**physician documentation**	Broad match
**soarian hospital software**	Broad match
**epic ehr pricing**	Exact match
**ecw training**	Broad match
**electronic medical records timeline**	Exact match
**care everywhere medical records**	Broad match
**introduction to health information management pdf**	Broad match
**how to improve healthcare**	Broad match
**degrees in health information technology**	Broad match
**c19 clinical trials**	Phrase match (close variant)
**healthcare management**	Broad match

**what does ehr mean in medical terms**	Broad match
**conemaugh mychart sign in**	Broad match
**rural healthcare program**	Broad match
**population health certification**	Broad match
**copilot health management system**	Phrase match (close variant)
**john hopkins risk stratification tool**	Broad match
**software mental health**	Broad match
**medical records online courses**	Phrase match (close variant)
**department health guidelines**	Broad match
**next gen health**	Broad match
**american medical software**	Phrase match (close variant)
**data entry in healthcare**	Broad match
**patient communication platform**	Broad match
**medical laboratory management software**	Phrase match (close variant)
**medical software name**	Phrase match (close variant)
**healthcare scheduling**	Broad match
**nextgen emr**	Broad match
**wound care documentation software**	Broad match
**intergy emr**	Phrase match (close variant)
**manager technical & operations health nutrition and populat**	Broad match
**medical record service**	Broad match
**hospital patient records**	Exact match
**meditech training courses**	Broad match
**types of healthcare data analytics**	Broad match
**chronic care management program cms**	Broad match
**medical practice management systems**	Broad match
**kinnser home health software**	Phrase match (close variant)
**medical mutual disease management program**	Broad match
**care connect epic**	Broad match
**clevelandclinlc org mychart**	Broad match
**medical record information**	Broad match
**electronic health record examples**	Exact match

**how to evaluate patient understanding**	Broad match
**health care data quality**	Broad match
**ehr corn**	Phrase match (close variant)
**medical record programs**	Broad match
**free online medical records courses**	Broad match
**healthcare management software solutions**	Exact match (close variant)
**how to check patients vital signs**	Broad match
**hhs ehr**	Exact match
**free online medical forms**	Broad match
**patient health record system**	Exact match (close variant)
**what is an empi**	Broad match
**rio medical records**	Broad match
**best dermatology emr software**	Phrase match (close variant)
**natural immunity study cleveland clinic**	Broad match
**medical billing program**	Broad match
**saas clinical trials**	Broad match
**epic emr training online**	Broad match
**new patient onboarding software**	Broad match
**how to use electronic health records**	Exact match
**cdc guidelines for tb testing**	Broad match
**healthcare consulting firms**	Broad match
**privacy medical**	Broad match
**tb skin test**	Exact match (close variant)
**private healthcare equity**	Broad match
**care coordination platform**	Broad match
**list down five possible solution that research can do for us t**	Broad match
**patient billing system**	Broad match
**advancedmd**	Broad match
**epic medical software training online**	Broad match
**lean daily management for healthcare**	Broad match
**software for home health agency**	Phrase match (close variant)
**patient chart template**	Broad match

**medical records courses online**	Broad match
**medical staffing software**	Phrase match (close variant)
**athenahealth training modules**	Broad match
**unstructured data healthcare**	Broad match
**emr medical terminology**	Broad match
**electronic health information**	Exact match
**digital doctor program**	Broad match
**medical records template free**	Broad match
**best crm for healthcare**	Phrase match (close variant)
**ehr systems**	Exact match (close variant)
**improving electronic health records**	Exact match
**cova 19**	Broad match
**emr electronic medical record software**	Exact match (close variant)
**workday psa healthcare**	Broad match
**medical billing and coding program**	Broad match
**academic electronic health record**	Exact match
**hospital automation**	Broad match
**urgent care emr systems**	Broad match
**pfizer clinical trial documents**	Broad match
**balanced scorecard in healthcare**	Broad match
**healthcare management app state**	Broad match
**athena health billing services**	Broad match
**epic electronic health record training**	Broad match
**patient scheduling technology**	Broad match
**population health strategy**	Broad match
**best ehr for pain management**	Phrase match (close variant)
**epic emr**	Exact match
**ehr health and medical records system**	Exact match
**xsolis com**	Broad match
**personify healthcare**	Broad match
**medical software company epic**	Phrase match (close variant)
**intella health**	Broad match

**how to obtain a copy of medical records**	Broad match
**what are the symptoms of this new variant**	Broad match
**electronic emr**	Exact match (close variant)
**what is titer testing**	Phrase match (close variant)
**medical decision making documentation examples**	Broad match
**best urgent care emr**	Broad match
**how to use epic medical software**	Exact match
**treatment center software**	Broad match
**folx health coupon**	Broad match
**healthcare governance**	Broad match
**hcs healthcare systems**	Broad match
**updox**	Broad match
**electronic patient forms**	Broad match
**benefits systems manager healthcare**	Broad match
**patient portal account access**	Broad match
**hospital software price**	Broad match
**human technology interface in nursing**	Broad match
**md logic health**	Broad match
**health information mgmt**	Broad match
**advance md**	Broad match
**list of disease management programs**	Broad match
**epic ehr for small practice**	Broad match
**sotrovimab clinical trials**	Broad match
**how do i access my medical records**	Broad match
**vision software healthcare**	Broad match
**population health management pdf**	Broad match
**hospital management software reviews**	Phrase match (close variant)
**computerized physician order entry cpoe**	Broad match
**ehr**	Broad match
**what are the advantages of electronic medical records**	Exact match
**how can i learn epic software**	Exact match
**emr epic training**	Broad match

**virtual research studies**	Broad match
**elation health**	Broad match
**ucsc e health**	Phrase match (close variant)
**coivd pcr test**	Broad match
**listed healthcare software companies**	Broad match
**chime college of healthcare information management**	Broad match
**medical record keeping cme online**	Broad match
**behavioral health ehr**	Phrase match (close variant)
**meditech emr**	Phrase match (close variant)
**patient relationship software**	Broad match
**conduent case management**	Broad match
**medication management platform**	Broad match
**best healthcare crm**	Phrase match (close variant)
**my lab test results**	Broad match
**healthcare billing systems**	Broad match
**patient portal software**	Phrase match (close variant)
**electronic medical record**	Exact match
**medical records coordinator remote**	Broad match
**electronic medical record definition**	Exact match
**how to test immune system strength**	Exact match (close variant)
**online patient records**	Exact match
**qualifacts**	Broad match
**surgical information system**	Broad match
**healthcare informatics consultant**	Broad match
**medical forms template**	Broad match
**medtrainer credentialing**	Broad match
**patient navigator program**	Broad match
**online patient portal medical records**	Broad match
**enable healthcare**	Broad match
**activ 2 study**	Broad match
**hospital system**	Broad match
**emr lab**	Phrase match (close variant)

**medical record access**	Broad match
**electronic medical record emr**	Exact match
**medical software names**	Phrase match (close variant)
**healthcare reporting**	Broad match
**titer**	Exact match (close variant)
**ophthalmology ehr**	Phrase match (close variant)
**nursing home software**	Broad match
**best statistical software for medical research**	Broad match
**home health charting software**	Phrase match (close variant)
**electronic health record forms**	Exact match
**comorbidity management**	Broad match
**clinical trials 2022**	Broad match
**medical patient software**	Exact match (close variant)
**blockchain medical records**	Broad match
**owner of epic software**	Exact match
**allscripts competitors**	Broad match
**healthcare billing software**	Broad match
**american healthtech**	Broad match
**cerner certification program**	Broad match
**medical diagnosis software for doctors**	Broad match
**health records management**	Broad match
**health site**	Broad match
**medical booking software**	Phrase match (close variant)
**why digital health is important**	Broad match
**epic system training**	Broad match
**digital healthcare statistics**	Broad match
**tidy health phr**	Broad match
**molecular laboratory software**	Broad match
**pfizer clinical trials documents**	Broad match
**health primary care**	Broad match
**rehabilitation documentation software**	Broad match
**management problems in healthcare**	Broad match

**hospital management**	Broad match
**health care health services**	Broad match
**benefits of an electronic health record**	Exact match
**best ehr for mental health private practice**	Broad match
**physical therapist software**	Broad match
**rheumatologist**	Broad match
**practice fussion**	Broad match
**health care management systems**	Exact match (close variant)
**functional medicine software**	Phrase match (close variant)
**credible emr**	Phrase match (close variant)
**healthcare research**	Broad match
**evolve telemed**	Broad match
**orthopedic ehr**	Broad match
**best ehr systems**	Broad match
**health information management careers**	Broad match
**what is an emr**	Exact match (close variant)
**endocrinology learning management system**	Broad match
**definition meaningful use**	Broad match
**patient access**	Broad match
**epic mychart sign in**	Broad match
**hospital readmission risk assessment tool**	Broad match
**hypertension disease management program**	Broad match
**medicare annual wellness questionnaire form**	Broad match
**how to get my medical records**	Broad match
**hcq studies**	Broad match
**epic emr training videos**	Broad match
**mychart university of chicago medicine**	Broad match
**healthcare hr software**	Broad match
**new treatments for depression 2021**	Broad match
**tb testing requirements by state**	Broad match
**moderna vaccine**	Broad match
**emrx**	Broad match

**healow patient portal sign in**	Broad match
**first healthcare compliance**	Broad match
**alberta health records online**	Phrase match (close variant)
**my medical records free online**	Broad match
**health tracking software**	Broad match
**health services management**	Broad match
**medical equipment tracking software tool canada**	Broad match
**medical patient software**	Broad match
**patient record management system**	Broad match
**healthcare patient portal**	Broad match
**patient satisfaction questionnaires**	Broad match
**health catalyst partners**	Broad match
**how to get your medical records online**	Broad match
**program management healthcare**	Broad match
**pfizer trial**	Broad match
**epic system ehr**	Exact match
**medilab**	Broad match
**nursing home compare website cms**	Broad match
**eprescribing systems**	Broad match
**hospital app**	Broad match
**healthcare saas**	Phrase match (close variant)
**advantages of electronic health records**	Exact match
**get your medical records**	Broad match
**doctor appointment booking platform**	Broad match
**medical manager program**	Broad match
**managing health services organizations and systems pdf fre**	Broad match
**personal health record**	Exact match (close variant)
**free epic emr training online**	Broad match
**epic systems healthcare**	Broad match
**digital health news**	Broad match
**population health models**	Broad match
**lab clinical trials**	Broad match

**patient access to lab results**	Broad match
**patient care advocate**	Broad match
**medhost hms training manual**	Broad match
**free epic training online**	Broad match
**patient estimation software**	Broad match
**medical records course**	Broad match
**va health records**	Exact match
**telemedicine platform**	Broad match
**health science**	Broad match
**clinically integrated network**	Broad match
**the future of electronic medical records**	Exact match
**advantages and disadvantages of the internet as a platform**	Broad match
**how to get allscripts certification**	Broad match
**autoimmune screening tests**	Broad match
**what is epic systems for hospitals**	Broad match
**health information management services**	Broad match
**caregiver scheduling software**	Broad match
**pfizer clinical trial**	Exact match (close variant)
**heath care management**	Broad match
**emr ehr systems**	Exact match
**importance of electronic health records**	Exact match
**healthcare data management companies**	Broad match
**3m medical coding software**	Phrase match (close variant)
**epic charting for nurses 2020**	Broad match
**vaccination experimental study**	Broad match
**fhcp**	Broad match
**myhealthone patient portal**	Broad match
**healthcare facilities normally schedule services that are**	Broad match
**compumed**	Broad match
**how long does it take to get a doctor referral**	Broad match
**electronic health records requirements**	Exact match
**johns hopkins study**	Broad match

**what is in medical records**	Exact match
**free hospital software**	Phrase match (close variant)
**transitional care program**	Broad match
**nextech emr training**	Broad match
**vaccine study 55 years**	Broad match
**medical records manager**	Phrase match (close variant)
**employee benefit plan management software**	Broad match
**ehr benefits**	Exact match
**practice software**	Broad match
**harris health system forms**	Broad match
**medical scheduling programs**	Broad match
**population health management trends**	Broad match
**how to get your medical license**	Phrase match
**electronic referral management system**	Broad match
**what is population health management**	Broad match
**cerner lab system**	Broad match
**health assessment online**	Broad match
**epic systems**	Exact match
**medical billing systems**	Broad match
**acurian health near me**	Broad match
**effects of the pandemic on students**	Broad match
**epic ambulatory training videos**	Broad match
**hospice documentation tools**	Broad match
**mobile apps related to health**	Broad match
**athenahealth**	Broad match
**medical document**	Exact match (close variant)
**epic computer program**	Broad match
**best opd management software**	Phrase match (close variant)
**safety management software**	Broad match
**best ehr for behavioral health**	Phrase match (close variant)
**clevelandclinic org mychart**	Broad match
**patient portal account setup**	Broad match

**doctor office software**	Broad match
**epic hyperspace training**	Exact match
**how to use epic charting for nurses**	Broad match
**free printable medical history forms**	Broad match
**moderna research study**	Phrase match (close variant)
**pfizer leaked**	Broad match
**long term care ehr**	Broad match
**p4p programs**	Broad match
**how to learn epic medical software**	Phrase match (close variant)
**medical information & research san antonio tx**	Phrase match (close variant)
**electronic health records articles**	Exact match
**digital health records**	Exact match
**free printable medical forms**	Broad match
**myhealthone hca**	Broad match
**point of care charting**	Broad match
**medical records tech jobs**	Phrase match (close variant)
**first line medical login**	Broad match
**mcleodhealth org your patient portal**	Broad match
**personal medical record software**	Broad match
**vaccine trials**	Exact match (close variant)
**phm healthcare**	Broad match
**electronic medical records standards**	Exact match
**platform healthcare solutions**	Broad match
**cerner electronic medical record**	Broad match
**patient affordability programs**	Broad match
**epic system for hospitals**	Exact match
**what is the foundation of the pathways to population health**	Broad match
**software applications in healthcare**	Exact match (close variant)
**patient care training program**	Broad match
**how to assess patient**	Broad match
**medical database management**	Broad match
**medical software demo**	Broad match

**hospital crm software**	Broad match
**evolent**	Broad match
**occupational health emr**	Phrase match (close variant)
**learn epic healthcare software free**	Phrase match (close variant)
**ehr health and medical records system**	Broad match
**free e prescribing**	Broad match
**focused patient assessment**	Broad match
**health care management companies**	Broad match
**docutap**	Broad match
**how to manage a medical office**	Broad match
**open emr**	Broad match
**personal health history form**	Phrase match (close variant)
**greenway computer program**	Broad match
**my uh personal health record**	Phrase match (close variant)
**healthcare job training programs**	Broad match
**medical billing courses online free**	Broad match
**epic healthcare software training**	Phrase match (close variant)
**electronic health records management**	Exact match (close variant)
**epic healthcare software training online**	Broad match
**epic medical charting tutorial**	Broad match
**best medical coding software**	Phrase match (close variant)
**community health program**	Broad match
**cerner health**	Broad match
**home care learning management system**	Broad match
**free medical records training online**	Broad match
**health management company**	Broad match
**queensland university vaccine**	Broad match
**wellsky charting**	Broad match
**ehr health records management system**	Phrase match (close variant)
**my chart doctor**	Broad match
**zeomega**	Broad match
**care pathway**	Broad match

**health questionnaire template**	Broad match
**healthcare grc software**	Broad match
**pandemic study**	Broad match
**emt and paramedic**	Broad match
**world health organization solidarity trial**	Broad match
**health management software**	Exact match
**medical trials near me**	Broad match
**healthcare and software**	Exact match (close variant)
**kyruus**	Broad match
**phizer data**	Broad match
**vistalink health**	Broad match
**workflow examples in healthcare**	Broad match
**pfizer trials**	Exact match (close variant)
**healthcare consulting software**	Broad match
**paid clinical trials 2022**	Broad match
**care management companies**	Broad match
**patient manager software**	Broad match
**cerner course**	Broad match
**medical md website**	Broad match
**what is adt in healthcare**	Broad match
**online patient appointment**	Broad match
**mental health ehr**	Broad match
**electronic health record ehr system**	Exact match
**healthcare applications**	Broad match
**best ehr for mental health**	Phrase match (close variant)
**point of care software**	Broad match
**how to use nextgen software**	Broad match
**advanced data systems**	Broad match
**system development life cycle in healthcare**	Broad match
**care healthcare**	Broad match
**my health record patient portal**	Broad match
**eclinicalworks emr login**	Broad match

**medical records training courses**	Broad match
**health cloud consultant**	Broad match
**charting patient care**	Broad match
**high paying medical trials**	Broad match
**care program requirements**	Broad match
**process used to monitor and control health care quality and**	Broad match
**ehr systems for therapists**	Broad match
**app for medical students**	Broad match
**medical checklist template**	Broad match
**optum and change healthcare**	Broad match
**best medical software**	Phrase match (close variant)
**pfizer trials**	Broad match
**population health news**	Broad match
**medical history questionaire**	Broad match
**coreo health**	Broad match
**medical case management software**	Phrase match (close variant)
**telemedicine study**	Broad match
**health systems**	Broad match
**plastic surgery emr**	Broad match
**free online medical information**	Phrase match (close variant)
**cleveland clinic study on natural immunity**	Broad match
**medical education software**	Broad match
**cdc vaccine study**	Broad match
**patient portals**	Broad match
**health care access program**	Broad match
**managers in health care**	Broad match
**creating a patient portal**	Broad match
**hospital to home program**	Broad match
**crspr vaccine**	Broad match
**mawson vaccine study**	Broad match
**my health records portal**	Phrase match (close variant)
**cancer patient assistance program**	Broad match

**medical tracking software**	Broad match
**medical record week**	Phrase match
**clinical key**	Broad match
**free emr training online**	Broad match
**length of time to keep medical records**	Broad match
**personal health**	Broad match
**penn medicine endocrinology**	Broad match
**population health model of care**	Broad match
**global vaccine access**	Broad match
**future of digital healthcare**	Broad match
**healthcare database systems**	Broad match
**online patient portal service**	Broad match
**medical record management system**	Broad match
**interactive patient care**	Broad match
**epic medical records training video**	Broad match
**pcr test in the bronx**	Broad match
**how to get medical records online**	Phrase match
**patient health portal**	Broad match
**the result of isolation**	Broad match
**patient medical records coordinator**	Broad match
**healthcare analytics companies**	Broad match
**blood management program**	Broad match
**doctor's office questionnaire**	Broad match
**healthcare data mining**	Broad match
**healthcare software startups**	Phrase match (close variant)
**affordable medical billing**	Broad match
**medical data technology**	Broad match
**laboratory software**	Broad match
**new patient forms printable**	Broad match
**electronic mental health records software canada**	Broad match
**facets healthcare tutorial**	Broad match
**patient feedback software**	Broad match

**medical coding software**	Phrase match (close variant)
**patient education websites**	Broad match
**healthicity**	Broad match
**best care management software**	Phrase match (close variant)
**epic workload acuity score**	Broad match
**athena one**	Broad match
**what are health records**	Exact match
**dhhs program grant 2021**	Broad match
**athena software tutorial**	Broad match
**what are the advantages of electronic health records**	Exact match
**case study in health care**	Broad match
**best app for medical students**	Broad match
**medical records training online free**	Broad match
**medical chart**	Phrase match (close variant)
**clinical systems in healthcare**	Broad match
**methodist patient portal login**	Broad match
**medical notes**	Broad match
**healthcare data entry**	Broad match
**mha graduate programs**	Broad match
**healthcare audit software**	Phrase match (close variant)
**medical records training online free**	Phrase match (close variant)
**my chart login page**	Broad match
**start medical billing program**	Broad match
**medical studies in my area**	Broad match
**best emr for pain management**	Broad match
**free medical records training**	Broad match
**nvax vaccine**	Broad match
**sweden natural immunity study**	Broad match
**prognos health revenue**	Broad match
**health management website**	Broad match
**medical records system**	Exact match (close variant)
**healthcare certificate program**	Broad match

**how to write a medical chart**	Broad match
**electronic medical records information**	Exact match
**population health company**	Broad match
**offering telemedicine**	Broad match
**medical dashboard**	Broad match
**free cehrs practice test**	Broad match
**health record**	Exact match
**health record template**	Broad match
**precision medicine software**	Phrase match (close variant)
**redox ehr**	Broad match
**school electronic health records**	Exact match
**therapist notes software**	Broad match
**mynovant org**	Broad match
**dm education for patients**	Broad match
**best emr for urgent care**	Broad match
**abbott id now test pcr**	Broad match
**simple admit patients start here**	Broad match
**meritus health my chart**	Broad match
**physician documentation templates**	Broad match
**primesuite**	Broad match
**electronic health record project implementation manager**	Broad match
**virtual health and wellness center**	Broad match
**phreesia cost estimation**	Broad match
**epic emr system**	Phrase match (close variant)
**electronic medical records uses**	Exact match
**patient tracking**	Broad match
**ehr systems training**	Broad match
**remote medical records jobs**	Phrase match (close variant)
**healthcare management pdf free download**	Broad match
**medical practice scheduling software**	Broad match
**apex emr**	Phrase match (close variant)
**syntellis change healthcare**	Broad match

**dossia personal health record**	Broad match
**free electronic medical records training online**	Broad match
**find medical information**	Broad match
**aetna case management program**	Broad match
**best ophthalmology emr**	Broad match
**epic ehr training video**	Broad match
**medical chart analysis**	Broad match
**medical report sample**	Phrase match (close variant)
**nextech emr tutorial**	Broad match
**list of epic certifications**	Broad match
**eclinicalworks version 11 user guide**	Broad match
**my health record sign up**	Broad match
**swedish study pfizer dna liver**	Broad match
**what are the benefits of electronic health records**	Exact match
**healthcare assistance programs**	Broad match
**medical records technician certification training**	Phrase match (close variant)
**digital health landscape 2021**	Broad match
**mgma certificate program**	Broad match
**public health information systems**	Broad match
**go to my chart**	Broad match
**patient care software**	Broad match
**what is healthec**	Broad match
**sunwave health**	Broad match
**medical questions and answers free**	Broad match
**about medical**	Broad match
**healthcare professional account management software**	Phrase match (close variant)
**hospital billing system software**	Broad match
**medical billing system**	Broad match
**electronic medical records**	Exact match (close variant)
**sharecare medical records**	Phrase match (close variant)
**make a doctor's appointment**	Broad match
**health information system**	Exact match (close variant)

**epic emr training 2020**	Broad match
**scrum methodology in healthcare**	Broad match
**excel patient database template**	Broad match
**performance measures in healthcare**	Broad match
**behavioral health emr**	Broad match
**health records online**	Broad match
**pre screening clinical trial**	Broad match
**medical claims software**	Phrase match (close variant)
**electronic health record and patient safety**	Exact match
**electronic health**	Broad match
**medical records director**	Phrase match (close variant)
**trinity medical center electronic medical record emr**	Broad match
**molnupiravir study data**	Phrase match (close variant)
**myhealthone**	Broad match
**what is patient registration**	Broad match
**enterprise applications for healthcare**	Phrase match (close variant)
**lcmc health login**	Broad match
**how to start a chronic care management program**	Broad match
**why electronic medical records are important**	Exact match
**pre screening checklist**	Broad match
**course of remdesivir**	Broad match
**clinical quality assessment**	Broad match
**medical software online**	Broad match
**director of population health**	Broad match
**goldcare telemedicine**	Broad match
**patient records online**	Broad match
**emr ehr systems**	Broad match
**health fusion claims**	Broad match
**population health navigator**	Broad match
**healthcare management consulting firm**	Broad match
**history of electronic health records in usa**	Exact match
**medical staffing software**	Broad match

**release of pfizer vaccine data**	Broad match
**online health tests**	Broad match
**amita health**	Broad match
**mdsave program**	Broad match
**organizing in healthcare management**	Broad match
**hiims**	Broad match
**health care population**	Broad match
**free emr systems**	Phrase match (close variant)
**athena emr training**	Broad match
**medical biller**	Broad match
**healthfusion emr**	Broad match
**emr system**	Exact match (close variant)
**omnicell**	Broad match
**medical records coordinator**	Phrase match (close variant)
**benefits of electronic health record**	Exact match
**medical software**	Broad match
**envision healthcare**	Broad match
**electronic health and billing records**	Exact match
**ethics of healthcare a guide for clinical practice**	Broad match
**health systems information**	Broad match
**hospital report format**	Broad match
**paid clinical research**	Broad match
**remdesivir clinical data**	Broad match
**avail clinical research**	Broad match
**epic certification free online training**	Broad match
**patient initial assessment form**	Broad match
**presenting a patient**	Broad match
**shot testing**	Exact match (close variant)
**condition management programs**	Broad match
**telemedicine memphis**	Broad match
**emr companies**	Phrase match (close variant)
**rgv healthcare management systems**	Phrase match (close variant)

**telehealth case studies**	Broad match
**vaccines and other biological products advisory committee**	Broad match
**fda pfizer**	Broad match
**emr medical records**	Exact match (close variant)
**health insurance management system**	Broad match
**medical e learning platform**	Broad match
**enterprisehealth**	Broad match
**medical records online courses**	Broad match
**health management information system hmis**	Broad match
**healtharc**	Broad match
**medical practice assessment**	Broad match
**digital health record**	Broad match
**point and click emr**	Broad match
**epic clindoc training**	Broad match
**population health management services**	Broad match
**simple practice ehr**	Phrase match (close variant)
**medical software solutions**	Broad match
**public health guidelines**	Broad match
**e clinical emr system**	Phrase match (close variant)
**pfizer documents side effects**	Broad match
**msk digital health companies**	Broad match
**population health care management**	Broad match
**patient records example**	Phrase match (close variant)
**medical access program**	Broad match
**patient healthcare**	Broad match
**best pain management emr**	Phrase match (close variant)
**software for medical records**	Broad match
**free patient management software**	Phrase match (close variant)
**best medical information app**	Broad match
**epic ehr training video 2021**	Broad match
**charting systems in hospitals**	Broad match
**how to create a hospital management database**	Broad match

**epic certification classes**	Broad match
**free patient records software**	Phrase match (close variant)
**athena emr training videos**	Broad match
**medical coder program online**	Broad match
**care360**	Broad match
**jefferson mychart**	Broad match
**medical form templates**	Broad match
**medical scheduling software**	Broad match
**paid online university research studies**	Broad match
**medical health records**	Broad match
**telehealth research articles**	Broad match
**business intelligence for healthcare**	Broad match
**population health education**	Broad match
**what is medical data systems**	Broad match
**novavax how it works**	Broad match
**odoo for healthcare**	Broad match
**electronic patient information**	Exact match
**personal health record**	Exact match
**free medical scribe certification course courses**	Broad match
**chronic care management companies**	Broad match
**using epic emr**	Exact match
**epic experience in healthcare**	Broad match
**free medical office software**	Broad match
**medical clinic system**	Broad match
**healtrh**	Broad match
**public health tech companies**	Broad match
**medical transportation software**	Broad match
**medical billing program online**	Broad match
**emr ehr**	Exact match (close variant)
**health information degree**	Broad match
**ehr charting**	Broad match
**my account on healthcare**	Broad match

**point click care for nurses**	Broad match
**electronic health records information**	Exact match
**population health program**	Exact match (close variant)
**doctor patient forms**	Broad match
**icu assessment sheet**	Broad match
**athena medical software training**	Broad match
**kareo billing pricing**	Broad match
**lawson healthcare software**	Phrase match (close variant)
**healthcare information technology company**	Broad match
**emd ehr**	Broad match
**how to be checked for lupus**	Broad match
**cerner charting system**	Broad match
**your health record**	Broad match
**how to become a vaccine researcher**	Broad match
**acep clinical guidelines**	Broad match
**online patient registration system**	Exact match (close variant)
**ucb cares patient assistance program**	Broad match
**patient triage form**	Broad match
**learn medical coding**	Broad match
**patient fusion portal**	Broad match
**nextgen emr**	Phrase match (close variant)
**icanotes behavioral health ehr**	Broad match
**ehr referral management**	Broad match
**matrix emr tutorial**	Phrase match (close variant)
**patient access training**	Broad match
**medical case management software**	Exact match (close variant)
**medcompass software**	Broad match
**data scientist healthcare**	Broad match
**healthcare software system**	Exact match (close variant)
**healthcare information technology companies**	Broad match
**health information database**	Broad match
**eclinicalworks com**	Broad match

**optum secure portal**	Broad match
**population heath**	Broad match
**population healthy agency**	Broad match
**prognocis emr tutorial**	Broad match
**vaccine jsu**	Broad match
**meditech charting**	Broad match
**patient portal account**	Broad match
**steward health care saba cloud**	Broad match
**management information system in hospitals**	Broad match
**healthcare vms systems**	Phrase match (close variant)
**clinicom**	Broad match
**share everywhere**	Broad match
**tb test**	Phrase match (close variant)
**epic computer system for hospitals**	Phrase match (close variant)
**reading tb tests**	Broad match
**harvard mask study**	Broad match
**my health one patient portal**	Broad match
**medical records clerk remote**	Phrase match (close variant)
**pfizer vaccine study**	Broad match
**patient assessment medical**	Phrase match (close variant)
**examples of health information systems**	Broad match
**pace university patient portal**	Broad match
**health information management education programs**	Broad match
**basic health assessment questionnaire**	Broad match
**my health one hca**	Broad match
**health information technology online program**	Broad match
**how to use epic emr**	Exact match
**athena health**	Broad match
**patient eligibility**	Broad match
**patient safety rounds checklist**	Broad match
**patient tracking system**	Broad match
**online lab reports**	Broad match

**epic healthcare software**	Exact match
**medical documents online**	Broad match
**hospital modules**	Broad match
**case management care coordination certificate program**	Broad match
**fill out medical forms online**	Broad match
**epic health care**	Exact match
**free online health risk assessment questionnaire**	Broad match
**digital health care services**	Broad match
**medical checklist**	Broad match
**life care planning software**	Broad match
**enterprise healthcare systems**	Broad match
**doctor appointment booking system**	Broad match
**telemedicine apps free**	Broad match
**sharing medical information**	Broad match
**electronic health record**	Exact match
**diabetes management software**	Broad match
**healthcare software companies jobs**	Broad match
**business intelligence healthcare**	Broad match
**health gorilla**	Broad match
**mychart sign in page**	Broad match
**secure telehealth platforms**	Broad match
**r e h**	Exact match
**genesis healthcare mychart**	Broad match
**what is emr**	Phrase match (close variant)
**uc research studies**	Broad match
**health care web design**	Broad match
**upmc research studies**	Broad match
**healthcare service design**	Broad match
**eclincal**	Broad match
**epic emr training for physicians**	Broad match
**electronic health records cons**	Exact match
**my medical records**	Broad match

**clinical information systems**	Phrase match (close variant)
**medical coding classes online free**	Broad match
**emr electronic medical record software**	Exact match
**practise management system**	Broad match
**epic system for healthcare**	Phrase match (close variant)
**what training and education programs are vital for healthcar**	Broad match
**doctor software**	Broad match
**patient record systems**	Exact match
**nextgen emr tutorial 2020**	Broad match
**prevounce**	Broad match
**myhealthone com**	Broad match
**university patient portal**	Broad match
**lumeris**	Broad match
**ehr systems**	Broad match
**clinical progress notes template**	Broad match
**gift card vaccine**	Broad match
**lexicomp**	Broad match
**healthcare analytics software**	Phrase match (close variant)
**healthelife portal registration**	Broad match
**patient management software**	Exact match (close variant)
**online health assessment**	Broad match
**medical booking software**	Broad match
**paid test studies**	Broad match
**medical coding for beginners**	Broad match
**health management information**	Exact match (close variant)
**advancedmd mental health**	Broad match
**novavax available us**	Broad match
**healthcare claims platform**	Broad match
**health management case studies**	Broad match
**medical practice software**	Broad match
**epic platform healthcare**	Broad match
**healthcare claims management software**	Phrase match (close variant)

**health management practices**	Broad match
**population health colloquium 2022**	Broad match
**ehr software company**	Broad match
**health fusion**	Broad match
**docutap training**	Broad match
**learn medical coding online free**	Broad match
**nurse charting guidelines**	Broad match
**health cloud**	Broad match
**online healthcare program**	Broad match
**medical facts to know**	Phrase match (close variant)
**ehr experience**	Broad match
**utilization management career**	Broad match
**articles on electronic health records**	Exact match
**software for medical coding**	Broad match
**electronic medical records vs electronic health records**	Exact match
**healthpoint**	Broad match
**moderna clinical trial**	Exact match (close variant)
**tb shot**	Broad match
**reading a tb skin test**	Broad match
**patient lab portal**	Broad match
**revenue cycle management system**	Broad match
**epic modules 2022**	Broad match
**how to learn epic software**	Exact match
**vaccination locations near me**	Broad match
**idx medical software**	Broad match
**mychart ku**	Broad match
**health tracking software**	Phrase match (close variant)
**healthcare administration training programs**	Broad match
**medical app for patients**	Broad match
**my health**	Broad match
**health care software system**	Exact match (close variant)
**emr for behavioral health**	Phrase match (close variant)

**population health companies**	Broad match
**health information health information**	Exact match (close variant)
**healthcare risk assessment template**	Broad match
**patient portal account login**	Broad match
**harris county health system**	Broad match
**health one tutorials**	Broad match
**patient advocacy programs**	Broad match
**master patient index mpi**	Broad match
**hospital bed management system**	Broad match
**clinical trials duke**	Broad match
**allscripts emr training**	Broad match
**healthcare grc**	Broad match
**what is epic company**	Exact match
**hospital work**	Broad match
**inpatient physician**	Broad match
**clinical decision support manager**	Broad match
**medisoft medical**	Broad match
**st davids myhealthone**	Broad match
**healow online patient portal**	Broad match
**how long does it take to make a vaccine**	Broad match
**examples of population health initiatives**	Broad match
**integrated hospital management system**	Broad match
**prevent 19 phase 3 trial**	Broad match
**electronic patient record software**	Broad match
**universal healthcare**	Broad match
**sales medical software**	Broad match
**allscripts cheat sheet**	Broad match
**baptist health mychart sign up**	Broad match
**cdi program for physicians**	Broad match
**electronic health records training**	Broad match
**billing software for medical offices**	Phrase match (close variant)
**patient portal health**	Broad match

**free printable tb test forms**	Broad match
**free healthcare forms**	Broad match
**infusion center software**	Broad match
**small practice management software**	Broad match
**online medical record**	Broad match
**clinical trials**	Broad match
**walk in lab tests**	Broad match
**doctor appointment scheduling app**	Broad match
**healthcare ehr system training specialist**	Phrase match (close variant)
**public healthcare technology companies**	Broad match
**health and wellness programs**	Broad match
**epic healthcare software training online courses**	Phrase match (close variant)
**is the pfizer shot safe**	Broad match
**goldcare health and wellness**	Broad match
**medical computer program**	Broad match
**electronic medical records**	Broad match
**cms healthcare learning management system**	Broad match
**epic healthcare software**	Phrase match (close variant)
**emr dermatology**	Broad match
**propath**	Broad match
**best medical crm**	Broad match
**healthcare incentive programs**	Broad match
**medical booklet**	Broad match
**ehr specialist jobs**	Broad match
**phelps health**	Broad match
**healthloop**	Broad match
**essentris**	Broad match
**healthcare analytics for quality and performance improveme**	Broad match
**what does a health information manager do**	Broad match
**epic tutorial for nurses**	Broad match
**epion health**	Broad match
**advantages of electronic medical records**	Exact match

**clinical management software**	Phrase match (close variant)
**advanced md**	Broad match
**university health system patient online**	Broad match
**johns hopkins stidy**	Broad match
**medical software consultant**	Broad match
**ehr systems for mental health providers**	Phrase match (close variant)
**epic charting for nurses**	Broad match
**danmask 19 study**	Broad match
**medical software developers**	Broad match
**mhs genesis**	Broad match
**dental clinical trials 2022**	Broad match
**ge api healthcare**	Broad match
**medical records coding technician**	Phrase match (close variant)
**mobile digital health**	Broad match
**patient intake software**	Broad match
**electronic health record article**	Exact match
**zeomega jiva**	Broad match
**leantaas**	Broad match
**new health management**	Broad match
**kareo software training**	Broad match
**opeeka**	Broad match
**oxford trials**	Broad match
**medical record systems**	Broad match
**world health organization remdesivir**	Broad match
**epic resolute certification**	Broad match
**medical record form**	Phrase match
**vitalworks**	Broad match
**free personal health record**	Broad match
**healthcare online programs**	Broad match
**symplr api healthcare**	Broad match
**cerner certification**	Broad match
**moderna trials**	Exact match (close variant)

**mental health care management**	Broad match
**hospitals that use cerner**	Broad match
**how to do electronic medical records**	Broad match
**personal health record service**	Broad match
**mental health emr systems**	Broad match
**population health software**	Broad match
**health information management**	Broad match
**electronic health records**	Exact match
**epic health record system**	Exact match
**caresync medical group**	Broad match
**hospital discharge forms**	Broad match
**population health technology companies**	Broad match
**cerner ehr training**	Broad match
**athenal health**	Broad match
**hcms**	Broad match
**medical record management**	Exact match (close variant)
**patient registration form**	Broad match
**medication knowledge base**	Broad match
**pharmacy software training**	Broad match
**physician concierge program**	Broad match
**emory blue portal**	Broad match
**israel antibody study**	Broad match
**healthcare billing**	Broad match
**case management ehr**	Broad match
**ohreesia**	Broad match
**conversa health**	Broad match
**inpatient medical coding**	Broad match
**future public health leaders program**	Broad match
**health usf edu care**	Broad match
**medical records technician**	Broad match
**epic emr pricing**	Exact match
**drug study uic edu**	Broad match

**digital health platform**	Exact match (close variant)
**c19study**	Phrase match (close variant)
**emory blue healthcare portal**	Broad match
**careconnect**	Broad match
**c digital health**	Broad match
**lockdown effectiveness study**	Broad match
**epic analyst training**	Broad match
**disease management companies**	Broad match
**care management assessment**	Broad match
**free personal health record software**	Broad match
**software solutions for hospitals**	Broad match
**electronic medical records conversion**	Broad match
**access my health record**	Broad match
**enterprise health**	Broad match
**hospital management system in java**	Broad match
**population health consulting**	Broad match
**health catalyst stock**	Broad match
**arcadia health**	Broad match
**family grant support program**	Broad match
**my medical record**	Exact match
**immune deficiency testing**	Phrase match (close variant)
**best ehr for psychiatry**	Broad match
**healthcare revenue cycle management companies**	Broad match
**healthcare survey**	Broad match
**pfizer report adverse effects**	Broad match
**how to get remdesivir treatment**	Broad match
**free emr for small practices**	Broad match
**health medical questions**	Broad match
**medecision**	Broad match
**dta healthcare solutions**	Broad match
**e clincal works**	Broad match
**leon medical connect**	Broad match

**healthcare cloud**	Broad match
**electronic patient record systems**	Exact match
**medical billing tools**	Broad match
**cons of electronic health records**	Exact match
**emr solutions**	Phrase match (close variant)
**best population health software**	Broad match
**hospital administration programs**	Broad match
**medical data entry**	Broad match
**center for population health**	Broad match
**epic training online**	Broad match
**healthy research studies**	Broad match
**pfizer vaccine injuries**	Broad match
**tracking patients in hospitals**	Broad match
**elctronic health records**	Exact match
**behavioral health documentation software**	Broad match
**patient management software open source**	Phrase match (close variant)
**nigeria healthcare system**	Broad match
**healthcare management system**	Exact match (close variant)
**doctors exam**	Broad match
**clinical study**	Broad match
**johns hopkins online medical information**	Broad match
**epic ehr mobile app**	Broad match
**titers testing**	Exact match (close variant)
**autoimmune testing**	Broad match
**how to learn emr**	Broad match
**emr mental health**	Phrase match (close variant)
**login to mychart**	Broad match
**epic emr**	Phrase match (close variant)
**health information management him analyst**	Broad match
**emdeon**	Broad match
**telemedicine software companies**	Broad match
**kareo patient portal**	Broad match

**wellness programs for healthcare workers**	Broad match
**elationemr com**	Broad match
**hospital softwares**	Broad match
**nextgen health fusion**	Broad match
**home healthcare software systems**	Phrase match (close variant)
**what is epic computer program**	Exact match
**health information management director**	Broad match
**accessing your own medical records**	Phrase match (close variant)
**what charting system does ascension use**	Phrase match (close variant)
**health screening questions**	Broad match
**epic medical**	Broad match
**jiva healthcare software**	Broad match
**optum behavioral health provider portal**	Broad match
**online patient registration software**	Exact match (close variant)
**go forward health**	Broad match
**how to promote a medical practice**	Broad match
**living with the virus**	Broad match
**patient tracking software**	Phrase match (close variant)
**doctors questionnaire**	Broad match
**mychart sign in**	Broad match
**home healthcare software solutions**	Phrase match (close variant)
**medical software system**	Exact match (close variant)
**best ehr for mental health**	Broad match
**pfizer clinical trial**	Broad match
**pcr test in columbus ohio**	Broad match
**paid research studies richmond va**	Broad match
**how to access my medical records online**	Broad match
**director population health**	Broad match
**collecting patient data**	Broad match
**pfizer clinical trial data**	Phrase match (close variant)
**patient fusion account**	Broad match
**emr ehr experience**	Broad match

**chronic care management program**	Broad match
**incident report health care**	Broad match
**medical charting**	Broad match
**scid screening test**	Broad match
**digital medical records software**	Exact match
**online medical record services**	Phrase match (close variant)
**electronic health record**	Exact match
**top practice management software**	Broad match
**treatment software**	Broad match
**rl solutions healthcare**	Broad match
**reverse transcription pfizer vaccine**	Broad match
**survey questions for healthcare**	Broad match
**epic emr login**	Exact match
**health managment**	Broad match
**free medical charting templates**	Broad match
**75health reviews**	Broad match
**myunity training**	Broad match
**mychart help desk**	Broad match
**health software development**	Broad match
**online medical lab tech program**	Broad match
**health mangement**	Broad match
**best online healthcare certificate programs**	Broad match
**oms organization**	Broad match
**meaningful use of electronic health record**	Exact match
**list of practice management software**	Broad match
**individual health plans**	Broad match
**healthcare management consulting**	Broad match
**epic emr system**	Broad match
**ut antibody study**	Broad match
**hospital management projects**	Broad match
**phr nursing**	Broad match
**practice management system software**	Broad match

**cerner emr**	Phrase match (close variant)
**center for cocid control**	Broad match
**free population health courses**	Broad match
**electronic health record ehr**	Exact match (close variant)
**health management information**	Broad match
**what is a health record**	Exact match
**how can i get medical records online**	Exact match
**programs for healthcare administration**	Broad match
**clinical erp**	Broad match
**patient self check in system**	Broad match
**medical record software**	Exact match (close variant)
**epic software for healthcare**	Phrase match (close variant)
**medical infor**	Broad match
**mental health ehr systems**	Phrase match (close variant)
**medical care**	Broad match
**patient pain assessment**	Broad match
**largest healthcare technology companies**	Broad match
**emr hospital software**	Exact match (close variant)
**epic connect care training**	Broad match
**medical waste management software**	Broad match
**epic hospital system training manual**	Broad match
**elationemr**	Broad match
**him health information management**	Phrase match (close variant)
**i2i population health**	Broad match
**medent emr tutorial**	Broad match
**prophylactic study**	Broad match
**healthcare consumerism**	Broad match
**consumer health platform**	Broad match
**healthcare software careers**	Broad match
**population focused nursing**	Broad match
**retrieve medical records**	Phrase match (close variant)
**aco management services llc**	Broad match

**epic hyperspace training**	Broad match
**med recs**	Broad match
**online healthcare training programs**	Broad match
**medical symptom checker**	Broad match
**free health care software**	Phrase match (close variant)
**software for clinic**	Broad match
**healthcare software systems**	Exact match (close variant)
**long term care nursing documentation**	Broad match
**electronic medical records physical therapy**	Broad match
**epic electronic health record software**	Phrase match (close variant)
**is there still a pandemic in the us**	Broad match
**medical software certification**	Broad match
**prevaccination screening questionnaire**	Broad match
**aethna health**	Broad match
**pros and cons of personal health records**	Broad match
**working at epic healthcare**	Exact match
**patients medical records**	Broad match
**encite emr**	Phrase match (close variant)
**admission of patients**	Broad match
**private laboratory testing**	Broad match
**ehr in healthcare**	Broad match
**centralized electronic health records**	Exact match
**dr question**	Broad match
**care plan**	Broad match
**medical softwear**	Exact match (close variant)
**home care management software**	Broad match
**epic population health**	Broad match
**paid medical studies near me**	Broad match
**cerner**	Broad match
**digital medical records**	Exact match
**research your health**	Broad match
**health care software**	Exact match

**wellsky home health**	Broad match
**fully funded healthcare degree programs**	Broad match
**healthcare access**	Broad match
**institute of health policy management and evaluation**	Broad match
**mckesson charting system**	Broad match
**medicine software**	Broad match
**medical office software free**	Phrase match (close variant)
**clinical decision support system software**	Broad match
**what is ehr in healthcare**	Broad match
**iot medical**	Broad match
**vaccine study exposes possible health concerns**	Broad match
**my patient portal online**	Broad match
**cerner charting training**	Broad match
**medical referral form pdf**	Broad match
**cleveland clinic health information**	Phrase match (close variant)
**my health records**	Phrase match (close variant)
**zynx health**	Broad match
**mental health software**	Broad match
**how to do a tb test**	Broad match
**epic electronic health record tutorial**	Broad match
**nextgen care**	Broad match
**patient portal nextmd com**	Broad match
**stay at home study at home**	Broad match
**followmyhealth account**	Broad match
**healthcare vendors list**	Broad match
**disease management platform**	Broad match
**patient relationship manager**	Broad match
**health information & referral programs**	Broad match
**who remdesivir study**	Broad match
**e health benefits**	Phrase match (close variant)
**medical documentation software**	Broad match
**behavioral health electronic medical records software unitec**	Phrase match (close variant)

**pfizer clinical trial data 2022**	Broad match
**medicare form cms 1490s**	Broad match
**epic for medical records**	Exact match
**physicians forms**	Broad match
**healthcare administration program**	Broad match
**patient care forms**	Broad match
**laboratory information system**	Broad match
**business intelligence in healthcare**	Broad match
**patient engagement programs**	Broad match
**login to patient portal**	Broad match
**lab reqs**	Broad match
**medical incident reports**	Broad match
**ohio health university learning management system**	Broad match
**raydiant health care**	Broad match
**online patient appointment system**	Broad match
**data analytics in healthcare**	Broad match
**kareo billing training**	Broad match
**how to get your medical history**	Broad match
**highest paid clinical trials near me**	Broad match
**hospital based clinical practice management**	Broad match
**patient information form template**	Broad match
**free electronic health records**	Phrase match (close variant)
**rn care manager**	Broad match
**novavax trials in us**	Broad match
**free rrt clinical simulation practice**	Broad match
**patient medical records**	Broad match
**free printable medical forms pdf**	Broad match
**start emmi program**	Broad match
**pfizer booster shot**	Broad match
**free software for patient record keeping**	Broad match
**the difference between pfizer and moderna shots**	Broad match
**online medical coder program**	Broad match

**chartmaxx**	Broad match
**medical records review jobs**	Phrase match (close variant)
**my chart help**	Broad match
**medical record certification form**	Broad match
**myhealthone com login**	Broad match
**best free emr**	Phrase match (close variant)
**medical crm system**	Broad match
**care continuum alliance**	Broad match
**verihealth**	Broad match
**prevent 19 clinical trial**	Broad match
**clinical reporting**	Broad match
**new patient registration forms**	Broad match
**portable health records**	Exact match
**mental health practice management**	Broad match
**electronic health records essay**	Exact match
**medical scheduler software**	Broad match
**vaccine data release**	Broad match
**medical studies in dallas tx**	Broad match
**efferent health platform**	Broad match
**healthcare applications examples**	Broad match
**emr in healthcare**	Exact match
**pku test**	Broad match
**patient software**	Broad match
**mychart now**	Broad match
**healthcare data platform**	Broad match
**emr prescription software**	Broad match
**healthcare technology**	Broad match
**epic medical billing software training pdf**	Broad match
**emr benefits for patients**	Exact match
**ehr for small practices**	Phrase match (close variant)
**tomorrow health**	Broad match
**medical records 2021**	Broad match

**new patient forms**	Broad match
**medical records emr**	Exact match
**patient data base**	Broad match
**how to get my medical records online**	Broad match
**free healthcare training programs**	Broad match
**epic ehr system**	Phrase match (close variant)
**free electronic health records software**	Phrase match (close variant)
**nursing software programs**	Broad match
**adz1222**	Broad match
**hospital information system providers in the philippines**	Broad match
**wound software**	Broad match
**population health management market**	Broad match
**clinicnotes**	Broad match
**phressia**	Broad match
**public health record**	Broad match
**emr training**	Exact match (close variant)
**population health director**	Broad match
**patients registration**	Broad match
**examples of population health**	Broad match
**hospital epic training**	Broad match
**health risk stratification**	Broad match
**medical records online training**	Broad match
**electronic health records book pdf**	Exact match
**digital health resources**	Broad match
**who enters the data into a personal health record phr**	Broad match
**how electronic health records work**	Exact match
**electronic health records ehr**	Exact match (close variant)
**epic emr for small practice**	Broad match
**e m t classes**	Exact match (close variant)
**healthcare inventory**	Broad match
**lockdown study**	Broad match
**emt**	Broad match

**medical staff scheduling and coverage coordinator**	Broad match
**medical records service**	Exact match (close variant)
**medical assistance programs**	Broad match
**service lines in healthcare**	Broad match
**is there a test to see if you already had covid 19**	Broad match
**personal medical records software**	Broad match
**hospital electronic medical records**	Exact match
**acurian health study**	Broad match
**praxissoftware**	Phrase match (close variant)
**paragon medical software**	Phrase match (close variant)
**health connections**	Broad match
**epic charting for nurses tutorial**	Broad match
**digital healthcare companies**	Broad match
**pfizer johnson**	Broad match
**programs for medical students**	Broad match
**cheapest emr**	Phrase match (close variant)
**lab diagnosis management system**	Broad match
**point click care log in**	Broad match
**population health programs**	Broad match
**map health management**	Broad match
**primary care assessment survey**	Broad match
**population health sciences cambridge**	Broad match
**novavax approval usa**	Broad match
**electronic health record systems software**	Broad match
**athena health training**	Broad match
**what is a care management program**	Broad match
**john hopkins atudy**	Broad match
**health information & services**	Broad match
**patients**	Broad match
**patient electronic health records**	Broad match
**rsv vaccine clinical trials**	Phrase match (close variant)
**epic computer system training**	Broad match

**pop health**	Broad match
**epic hospital software**	Phrase match (close variant)
**pfizer release data**	Broad match
**healthcare training programs**	Broad match
**free printable vitals log sheet**	Broad match
**medical billing coding program**	Broad match
**medical appointment management**	Broad match
**patient adherence programs**	Broad match
**top population health companies**	Broad match
**patient flow management**	Broad match
**health catlyst**	Broad match
**electronic medical records tutorial**	Broad match
**modmed ema login**	Broad match
**epic electronic medical records training**	Broad match
**autoimmune disease questionnaire**	Broad match
**health insurance software**	Broad match
**electronics health records**	Exact match
**liquid emr**	Phrase match (close variant)
**health advocate program**	Broad match
**telehealth clinical trials**	Broad match
**electronic health records then now and in the future**	Exact match
**online lab software**	Broad match
**hipaa compliant online forms**	Broad match
**cloud md**	Broad match
**healthcare revenue cycle management**	Broad match
**hospital info system**	Broad match
**my health records online**	Broad match
**meditech charting system**	Phrase match (close variant)
**electronic health records course**	Broad match
**healthcare surveys**	Broad match
**how long does it take for a pandemic to end**	Broad match
**advantages to electronic medical records**	Exact match

**iot healthcare use cases**	Broad match
**well 360 virtual health**	Broad match
**home health software vendors**	Broad match
**medical practice assessment tools**	Broad match
**utmb health my chart**	Broad match
**meditech live**	Broad match
**medical results template**	Broad match
**healthcare implementation project manager**	Broad match
**medical information research detroit mi**	Phrase match (close variant)
**proficient life healthcare**	Broad match
**hospital tracking systems**	Broad match
**hms healthcare**	Broad match
**digital health definition**	Broad match
**athenahealth tutorial**	Broad match
**health catalyst competitors**	Broad match
**john hopkins transplant vaccine study**	Broad match
**questions to ask patients**	Broad match
**the pfizer documents**	Broad match
**population health service**	Broad match
**epic software healthcare training**	Broad match
**medseek**	Broad match
**doctors scheduling software**	Broad match
**john's hopkins study**	Broad match
**population level health issues**	Broad match
**definition of an electronic health record**	Exact match
**novavax us trials**	Phrase match (close variant)
**learning during a pandemic**	Broad match
**epic systems corp**	Exact match
**healthcare it consulting**	Broad match
**how to study in quarantine**	Broad match
**hospital management programs**	Broad match
**ehr electronic health records system**	Exact match

**patient records**	Broad match
**pfizer clinical trials results**	Broad match
**electronic health records ehr systems**	Broad match
**dental record keeping courses**	Broad match
**credentialing platform**	Broad match
**mcn healthcare**	Broad match
**electronic health records essays**	Exact match
**doctor appointment booking software**	Broad match
**healthcare professionals**	Broad match
**epic training**	Broad match
**hospital management program**	Broad match
**medhealth**	Broad match
**how to access patient portal**	Broad match
**pre employment health screening questionnaire**	Broad match
**care access clinical trials**	Broad match
**medical training programs**	Broad match
**medical learning platform**	Broad match
**medical practice management software used to print patient**	Phrase match (close variant)
**hospital patient records**	Exact match (close variant)
**medical records request**	Broad match
**lippincott clinical experiences community public and populat**	Broad match
**emory medical records release form**	Broad match
**epic in healthcare**	Broad match
**ehr charting systems**	Broad match
**medical profile**	Broad match
**medicare annual wellness visit questionnaire**	Broad match
**ehr software systems**	Exact match (close variant)
**epic charting**	Broad match
**online patient registration system**	Broad match
**medical record certification form**	Phrase match
**free medical record access database**	Broad match
**moderna israel study**	Broad match

**hospital software programs**	Exact match (close variant)
**medical software used in hospitals**	Broad match
**enli health intelligence**	Broad match
**wuhan lab study**	Broad match
**medhost software**	Phrase match (close variant)
**health records online patient portal**	Broad match
**mybch org**	Broad match
**health information managment**	Exact match (close variant)
**patient forms**	Broad match
**johns hopkins medical center locations**	Broad match
**arcadia population health**	Broad match
**clinical communications**	Broad match
**healthcare it services**	Broad match
**medical health records**	Exact match
**medical database software**	Exact match (close variant)
**antigen test appointment booking**	Broad match
**free hospital discharge templates**	Broad match
**phm healthcare**	Phrase match (close variant)
**advancedmd log**	Broad match
**epic certification courses**	Broad match
**psychiatrist software**	Broad match
**hotdoc**	Broad match
**evolution of electronic health record**	Exact match
**define electronic medical record**	Exact match
**manager of medical records coding**	Broad match
**medical questions website**	Broad match
**whats a emr**	Phrase match (close variant)
**patient management system**	Exact match (close variant)
**medical record online**	Exact match
**ehr pros**	Exact match
**soarian**	Broad match
**healthvana**	Broad match

**healthcare accounting**	Broad match
**health care for families**	Broad match
**exscribe patient portal login**	Broad match
**patient information portal**	Broad match
**electronic medical record systems**	Exact match
**free healthcare benefits**	Broad match
**medical records electronic**	Exact match (close variant)
**physician critical care unit**	Broad match
**sigmacare training for nurses**	Broad match
**iot healthcare devices list**	Broad match
**patient review software**	Broad match
**medical patient form template**	Phrase match (close variant)
**medical simulation software**	Broad match
**emr for psychiatry**	Broad match
**analytical tools used to process data collection in healthcare**	Broad match
**patient access competency test**	Broad match
**healthcare marketing software**	Phrase match (close variant)
**use of electronic health records in u s hospitals**	Exact match
**eclinic**	Broad match
**unite us social determinants of health**	Broad match
**health check program**	Broad match
**population health management dashboard**	Broad match
**swedish study mrna dna**	Broad match
**healthcare compliance solutions**	Broad match
**allied health online programs**	Broad match
**medical software dictionary**	Broad match
**ob gyn emr**	Phrase match (close variant)
**online health management tools**	Phrase match (close variant)
**clinical management system**	Broad match
**epic modules training**	Broad match
**electronic health record features**	Exact match
**patient care technician**	Broad match

**epic health systems**	Exact match
**fda approved health apps**	Broad match
**clinical trials for money**	Broad match
**patient information**	Broad match
**patient**	Broad match
**printable health history questionnaire**	Broad match
**healthcare portal vendors**	Broad match
**digital healthcare company**	Broad match
**requirements for health records course**	Broad match
**patient registration process steps**	Broad match
**healthcare certification programs online**	Broad match
**patient discharge forms**	Broad match
**medical record review from home**	Phrase match
**patient now software**	Phrase match (close variant)
**epic healthcare reviews**	Broad match
**pfizer documents**	Broad match
**study on natural immunity**	Broad match
**cerner charting system for nurses**	Broad match
**admission protocols in hospitals**	Broad match
**best emr systems**	Phrase match (close variant)
**medical billing software free**	Phrase match (close variant)
**electronic medical records online courses**	Exact match
**ehr analyst**	Phrase match (close variant)
**st davids com myhealthone**	Broad match
**hospital computer system**	Broad match
**medical records electronic**	Exact match
**intergy ehr training**	Phrase match (close variant)
**prescribing guidelines for doctors**	Broad match
**healthcare database software**	Broad match
**mckesson lytec**	Broad match
**c19 study**	Exact match (close variant)
**referral management healthcare**	Broad match

**docutap login**	Broad match
**health language**	Broad match
**introduction to health information systems**	Broad match
**medical records clerk home care**	Phrase match (close variant)
**medical app**	Broad match
**medical device crm**	Broad match
**vaccine efficacy studies**	Broad match
**point click care**	Broad match
**free medical coding refresher courses**	Broad match
**expert medical opinion program**	Broad match
**digital health care companies**	Broad match
**login patient fusion**	Broad match
**clinical information system specialist**	Broad match
**health screenings by age**	Broad match
**medrio**	Broad match
**inpatient management software**	Broad match
**use of electronic health records**	Exact match
**pcr test st clair shores**	Broad match
**view medical records online**	Broad match
**medical file management**	Broad match
**patient health information**	Broad match
**knowledge sharing medical coding**	Broad match
**health information management technology**	Exact match (close variant)
**employee health software**	Broad match
**sas public health**	Broad match
**cms hospital at home program**	Broad match
**hospital workforce optimization**	Broad match
**medical chart templates free**	Broad match
**medical software systems**	Exact match
**medical advocate program**	Broad match
**best emr for physical therapy**	Broad match
**a patient portal is**	Broad match

**patient scheduling software**	Broad match
**mychart view my chart**	Broad match
**medline clinical trials**	Broad match
**crm and healthcare**	Broad match
**livongo program**	Broad match
**free tuberculosis testing**	Broad match
**discharge planning software**	Broad match
**hospital patient records**	Broad match
**pfizer vaccine data**	Broad match
**ehr softwares**	Exact match
**omron healthcare software**	Phrase match (close variant)
**cove study moderna**	Broad match
**hospital managment**	Broad match
**health dialog services corporation**	Broad match
**nextgen emr training**	Broad match
**epic ehr tutorial**	Broad match
**legal aspects of healthcare administration pdf free**	Broad match
**clinical data repository**	Broad match
**collective medical technologies**	Broad match
**dr peter mccullough reinfection**	Broad match
**health and informatics**	Broad match
**can electronic medical records be altered**	Exact match
**acurian clinical trial center**	Broad match
**learn vault health com nj**	Broad match
**medication therapy management platforms**	Broad match
**home health care software systems**	Phrase match
**healthcare utilization management software**	Phrase match (close variant)
**moderna booster**	Broad match
**free printable medical history form**	Broad match
**examples of physician queries**	Broad match
**medical practice management definition**	Broad match
**host benefits ehr com**	Exact match

**total health program**	Broad match
**learn epic emr online**	Phrase match
**smartcare for employees**	Broad match
**inovio**	Broad match
**medicare ccm program**	Broad match
**population health systems**	Broad match
**how to get epic certified**	Broad match
**medical record assistant**	Phrase match
**docdelta**	Broad match
**epic charting for nurses**	Phrase match (close variant)
**mychart account**	Broad match
**aht charting system**	Broad match
**medical patient registration form**	Phrase match (close variant)
**nextgen emr tutorial**	Broad match
**health records online**	Exact match
**hinge health program**	Broad match
**behavioral health emrs**	Broad match
**medical saas**	Broad match
**epic electronic health record tutorial**	Phrase match (close variant)
**patient appointment scheduling software**	Broad match
**health care computer programs**	Exact match (close variant)
**https mychart**	Broad match
**become a patient**	Broad match
**henry ford study**	Broad match
**advanced hospital management system**	Broad match
**hipaa compliant emr software**	Phrase match (close variant)
**surge immunity test**	Phrase match
**what is the purpose of electronic health records**	Exact match
**covishield clinical trials**	Broad match
**population health informatics**	Broad match
**data integrity issues in pfizer's vaccine trial**	Phrase match (close variant)
**epic ehr certification**	Phrase match (close variant)

**care partner program**	Broad match
**credentialing solution**	Broad match
**health management information system**	Broad match
**health care testing**	Broad match
**online healthcare management programs**	Broad match
**population health masters**	Broad match
**patient experience software**	Broad match
**crm in healthcare**	Broad match
**medical records on line**	Exact match (close variant)
**followmyhealth patient access**	Broad match
**memorial managed care plan provider portal**	Broad match
**chronic disease management apps**	Broad match
**epic charting for er nurses**	Broad match
**athena medical software**	Phrase match (close variant)
**kareo clinical ehr software**	Phrase match (close variant)
**what is a tb test**	Exact match (close variant)
**unite us healthcare**	Broad match
**novavax approved usa**	Broad match
**epic systems training**	Broad match
**virtual clinic software**	Broad match
**epic medical software**	Phrase match (close variant)
**isalus healthcare**	Broad match
**emr for physical therapy**	Broad match
**novavax clinical trials**	Broad match
**mmr titer test quest diagnostics**	Phrase match (close variant)
**neurology new patient questionnaire**	Broad match
**medical call center software**	Phrase match (close variant)
**medical records retrieval specialist**	Phrase match (close variant)
**oncology information system**	Broad match
**oscar medical software tutorial**	Phrase match (close variant)
**what is a electronic medical record**	Exact match
**therapy notes ehr**	Phrase match (close variant)

**onc certification**	Broad match
**cerner software**	Broad match
**how to use epic ehr system**	Broad match
**ehr companies**	Phrase match (close variant)
**patient studies**	Broad match
**health management companies**	Broad match
**patient flow system**	Broad match
**philips population health management**	Broad match
**medical records system**	Exact match
**medical record**	Exact match
**patient scheduling**	Broad match
**patient scheduling system**	Broad match
**practice fusion emr cost**	Broad match
**free patient access training**	Broad match
**telehealth software companies**	Broad match
**gateway health plan provider portal**	Broad match
**healthcare competency tool**	Broad match
**allscripts tutorials**	Broad match
**eclinicalworks ehr**	Broad match
**my medical records online**	Broad match
**why do we need electronic health records**	Exact match
**companies like phreesia**	Broad match
**medical records indexer**	Broad match
**new patient paperwork pdf**	Broad match
**covidm**	Broad match
**epic emr training free**	Phrase match (close variant)
**healthcare crm**	Broad match
**real health**	Broad match
**my continulink point of care**	Broad match
**epic emr training courses**	Broad match
**printable medical record form**	Broad match
**blood test for crohn's disease**	Broad match

**emr systems for hospitals**	Broad match
**medical practice management system**	Broad match
**health care records**	Broad match
**population focused**	Broad match
**epic software healthcare**	Phrase match (close variant)
**medconnect**	Broad match
**informatics healthcare**	Broad match
**c 19 clinical trials**	Phrase match (close variant)
**medaccess**	Broad match
**nextmed**	Broad match
**emr administrator**	Phrase match (close variant)
**patient scheduling online**	Broad match
**chiropractic billing software**	Broad match
**novavax research study**	Broad match
**patient management program**	Broad match
**epic medical system**	Phrase match (close variant)
**different types of emr systems**	Broad match
**mychart jefferson login**	Broad match
**health system**	Broad match
**remdesivir treatment**	Broad match
**emr trainer**	Broad match
**webpt tutorial**	Broad match
**electronic medical records lawsuit**	Exact match
**lis healthcare**	Broad match
**iot devices in healthcare**	Broad match
**epic resolute**	Broad match
**new patient forms for doctors**	Broad match
**medical crm software**	Phrase match (close variant)
**hospital admission software**	Broad match
**perspectives in care program**	Broad match
**chronic care management platform**	Phrase match (close variant)
**medical records assistant**	Phrase match (close variant)

**john hopkins population health**	Broad match
**misys healthcare**	Broad match
**telehealth products**	Broad match
**learn epic emr online**	Broad match
**employee benefits management system**	Broad match
**patient care portal**	Broad match
**case study telemedicine**	Broad match
**pfizer side effects**	Broad match
**hospital document**	Broad match
**epic outpatient tutorial**	Broad match
**health information pdf**	Broad match
**centricity electronic medical record**	Broad match
**lion healthcare management**	Broad match
**what is health information**	Broad match
**health information management specialist**	Broad match
**washington university study on natural immunity**	Broad match
**medical records free**	Phrase match (close variant)
**healthcare ehr systems**	Broad match
**wavemark**	Broad match
**medical research studies**	Broad match
**medical documentation examples**	Broad match
**intella triage**	Broad match
**health information technology programs**	Broad match
**medical coding platforms**	Broad match
**yourhealthfile patient portal**	Broad match
**login mychart com**	Broad match
**pcr test downtown chicago**	Broad match
**epic information system**	Broad match
**emr training**	Broad match
**patient relations**	Broad match
**amazing charts emr**	Phrase match (close variant)
**how to learn epic emr**	Broad match

**care radius**	Broad match
**electronic documentation in healthcare**	Broad match
**covd 19**	Broad match
**data entry healthcare**	Broad match
**medicaid care management programs**	Broad match
**chinese lab**	Broad match
**parkview hospital dupont road fort wayne in**	Broad match
**medical record template**	Broad match
**health information records technician**	Phrase match
**check your lab results online**	Broad match
**cerner soarian training**	Broad match
**best therapy notes software**	Broad match
**patient portal services**	Broad match
**population health dashboard**	Broad match
**icanotes**	Broad match
**patients health records**	Broad match
**purpose of electronic health records**	Exact match
**history of medical record keeping**	Phrase match
**health management resources program**	Broad match
**health history assessment questions**	Broad match
**population health management journal**	Broad match
**athenahealth emr training manual**	Broad match
**health assistance program**	Broad match
**utilization management tools**	Broad match
**clinovations**	Broad match
**therapy practice management software**	Broad match
**cardiology pacs**	Broad match
**emr medical software**	Exact match (close variant)
**online lab appointment scheduling system**	Broad match
**lab electronic health records ehr system**	Broad match
**medical assistance program**	Broad match
**health informatic programs**	Broad match

**epic certification**	Broad match
**healthcare it training**	Broad match
**medical office software**	Broad match
**the impact of electronic health records**	Exact match
**one touch emr**	Phrase match (close variant)
**ehr facts**	Exact match
**phizer clinical trial**	Broad match
**clinical notes template**	Broad match
**population health management strategies**	Broad match
**patient education software**	Broad match
**patient medical information**	Exact match (close variant)
**epic tutorial healthcare**	Broad match
**clinical trials moderna**	Broad match
**phreesia check in on computer**	Broad match
**patient health records**	Exact match (close variant)
**omicell**	Broad match
**online medical records for patients**	Broad match
**electronic charting in hospitals**	Exact match
**intergy software training**	Broad match
**care management models**	Broad match
**sign up for patient portal**	Broad match
**online patient forms**	Broad match
**pfizer vaccine**	Broad match
**human and health services program**	Broad match
**free medical case studies**	Broad match
**healthcare erp solutions**	Broad match
**health status questionnaire**	Broad match
**how to be a good patient**	Broad match
**patient evaluation form sample**	Broad match
**request medical records**	Broad match
**occupational health manager software**	Broad match
**hospital management system india**	Broad match

**remdesivir trial studies**	Broad match
**vaccine mandate**	Broad match
**remote medical records medical data reviewer**	Phrase match (close variant)
**mvhealthsystem mychart**	Broad match
**patient billing services**	Broad match
**check in patients**	Broad match
**intergy ehr**	Broad match
**covidsheet**	Broad match
**how to request medical records**	Broad match
**electronic health care records**	Exact match
**free tb testing**	Broad match
**mychart saint francis healthcare system**	Broad match
**health general**	Broad match
**medent**	Broad match
**ehr systems**	Exact match
**nih study mixing vaccines**	Broad match
**medication systems management**	Broad match
**chronic care program**	Broad match
**nextgen healthcare tutorial**	Broad match
**eclinic labs**	Broad match
**medical form template**	Broad match
**rfp healthcare information system**	Broad match
**mrna side effects long term**	Broad match
**nextgen healthcare patient portal**	Broad match
**myhealth**	Broad match
**patient forms online**	Broad match
**how do i get a copy of my medical records from the hospital**	Broad match
**population health com**	Broad match
**my chart**	Broad match
**lms systems for healthcare**	Broad match
**patient management**	Broad match
**definition of population health management**	Broad match

**healow com login**	Broad match
**medical encounter forms**	Broad match
**patient portal software vendors**	Phrase match (close variant)
**pfizer data release side effects**	Broad match
**electronic medical charting systems**	Exact match
**how to use mychart app**	Broad match
**epic emr training 2021**	Broad match
**epic electronic medical record system**	Exact match
**vaccine control group**	Broad match
**jane ehr**	Broad match
**best crm software for healthcare industry**	Broad match
**e health records**	Exact match
**lab results portal**	Broad match
**hospital safety programs**	Broad match
**credible behavioral health software**	Broad match
**electronic medical record mandate**	Exact match
**moderna study**	Broad match
**medez**	Broad match
**valant emr**	Broad match
**emr epic system**	Exact match
**health catalyst revenue**	Broad match
**what are emrs**	Exact match (close variant)
**patient profiles**	Broad match
**patient check in procedure**	Broad match
**epic electronic health record system**	Phrase match (close variant)
**occupational therapy emr**	Phrase match (close variant)
**hospital contract management**	Broad match
**covi usa**	Broad match
**vitera medical manager**	Broad match
**www fdohpatientportal org registration**	Broad match
**personal health record software free**	Broad match
**electronic health record app**	Broad match

**template for medical records**	Broad match
**medical management programs**	Exact match (close variant)
**hospital certification programs**	Broad match
**eclinicalworks training free**	Broad match
**complete physical examination form**	Broad match
**retrieve medical records**	Broad match
**patient portal+**	Broad match
**antigen test near me for travel**	Broad match
**disease managment programs**	Broad match
**population health insurance**	Broad match
**source medical**	Broad match
**moderna studies**	Broad match
**population health**	Broad match
**patient referral management software**	Broad match
**secure my health**	Broad match
**patient appointment scheduling**	Broad match
**medicare wellness questionnaire 2021**	Broad match
**home care program**	Broad match
**chronic disease management software**	Broad match
**patient portal vendors**	Broad match
**astrazeneca research studies**	Broad match
**pop healthcare jacksonville fl**	Broad match
**phreshia**	Broad match
**how do you set up mychart**	Broad match
**electronic medical records benefits**	Exact match
**cost electronic health records**	Broad match
**home health compare**	Broad match
**emr systems for hospitals**	Exact match
**athen health**	Broad match
**medical network management software**	Phrase match (close variant)
**pcr test in my area**	Broad match
**healthcare it service**	Broad match

**health healow your patient portal**	Broad match
**digital health tools for patients**	Broad match
**paid research trial**	Broad match
**paciente**	Broad match
**ehr flow**	Broad match
**roles of stakeholders in healthcare**	Broad match
**what is general healthcare**	Broad match
**clinical decision support tools**	Broad match
**telemedicine locations**	Broad match
**experity patient portal**	Broad match
**chiropractic ehr systems**	Phrase match (close variant)
**asymtematic**	Broad match
**medical records jobs**	Phrase match (close variant)
**new patient forms primary care**	Broad match
**pittsburgh research studies**	Broad match
**hospital websites**	Broad match
**top 10 ehr implementation challenges and how to overcome**	Broad match
**health information technology management**	Phrase match (close variant)
**denmark study vaccine effectiveness**	Broad match
**nextgen healthcare usa**	Broad match
**healthcare data platforms**	Broad match
**electronic therapy notes**	Broad match
**healthcare systems management**	Exact match (close variant)
**adventhealth clinical trials**	Broad match
**optimize health**	Broad match
**health record software**	Broad match
**electronic health information systems**	Exact match
**home health care management software**	Phrase match (close variant)
**patient access software**	Broad match
**interface engines for healthcare**	Broad match
**is phizer effective**	Exact match (close variant)
**dental information system**	Broad match

**care management platforms**	Exact match (close variant)
**self assessment tools in healthcare**	Broad match
**population health mental health**	Broad match
**epic healthcare management**	Broad match
**hyland health**	Broad match
**healow patient portal app**	Broad match
**community and population health**	Broad match
**meditech expanse training manual pdf**	Broad match
**medical credentialing training programs**	Broad match
**erp for healthcare industry**	Broad match
**healthcare technology management**	Broad match
**api healthcare app**	Broad match
**student health record management system**	Phrase match
**what is himss**	Broad match
**epic emr**	Broad match
**software in healthcare**	Exact match (close variant)
**software emr**	Broad match
**online medical record services**	Broad match
**online medical billing coding programs**	Broad match
**medical patient forms free**	Phrase match (close variant)
**epic computer system for hospitals**	Exact match
**novavax us clinical trial**	Broad match
**best ehr systems**	Phrase match (close variant)
**health information tools**	Broad match
**livongo diabetes management program**	Broad match
**pfizer adverse event report**	Broad match
**idx healthcare software**	Phrase match (close variant)
**medical lab programs**	Broad match
**idx medical software tutorial**	Broad match
**patient appointment booking system**	Broad match
**seroprevalence study**	Broad match
**general electric healthcare**	Phrase match (close variant)

**https mynm nm org mychart**	Broad match
**online patient booking system**	Broad match
**health connect kaiser**	Broad match
**mychart log in**	Broad match
**electronic health records**	Broad match
**what is epic used for**	Exact match
**phizer studies**	Broad match
**phreesia sign on**	Broad match
**epic documentation training for nurses**	Broad match
**epichealth**	Exact match
**home health and hospice software**	Phrase match (close variant)
**online patient appointment scheduling**	Broad match
**how do i get my medical records**	Broad match
**electronic health records management**	Exact match
**psychiatry emr systems**	Broad match
**healthcare software developer**	Phrase match (close variant)
**digital health**	Phrase match (close variant)
**research on electronic health records**	Exact match
**epic health**	Exact match
**digital health technology**	Broad match
**john hopkins variant list**	Broad match
**masters of population health**	Broad match
**ehr application analyst ii**	Phrase match (close variant)
**phreesia training**	Broad match
**hospital administration system**	Broad match
**clevelandclinic org online services**	Broad match
**patient record template**	Broad match
**epiccare ambulatory certification**	Broad match
**livongo programs**	Broad match
**utilization management platform**	Broad match
**pfizer data release**	Broad match
**population health management ppt**	Broad match

**citigroup vaccine study**	Broad match
**health it specialist**	Broad match
**what is health information management**	Phrase match (close variant)
**clinical management**	Broad match
**printable medical forms**	Broad match
**focused health assessment**	Broad match
**healow health and online wellness**	Broad match
**hospital pharmacy software**	Broad match
**federal law electronic medical records**	Exact match
**program for appropriate technology in health**	Broad match
**tb testing free**	Broad match
**allscripts emr system**	Broad match
**epic mychart training**	Broad match
**urology emr**	Broad match
**bmc digital health**	Phrase match (close variant)
**ehr benefits**	Broad match
**synergy emr**	Phrase match (close variant)
**free online medical billing and coding certification programs**	Broad match
**athena emr system**	Phrase match (close variant)
**medical history template**	Broad match
**pupulation health**	Broad match
**epic for nurses**	Broad match
**programs healthcare**	Broad match
**john hopkins long haulers**	Broad match
**medical patient scheduling software**	Broad match
**free medical software**	Broad match
**healthcare development services**	Broad match
**health records**	Broad match
**what is the difference between emr and ehr**	Phrase match (close variant)
**electronic medical record vendor**	Exact match
**introduction of electronic health records**	Exact match
**top hospital software systems**	Broad match

**oncology emr**	Phrase match (close variant)
**epic emr training for nurses**	Broad match
**health records patient fusion**	Phrase match (close variant)
**healthcare scheduling software systems**	Broad match
**phr system**	Exact match (close variant)
**vaccination learning management system**	Broad match
**digitalhealth**	Broad match
**health management information system hmis**	Phrase match (close variant)
**medical websites health information**	Phrase match (close variant)
**tb skin test**	Phrase match (close variant)
**cerner log in powerchart**	Broad match
**sign up for mychart**	Broad match
**vivify health pricing**	Broad match
**online diagnosis free**	Broad match
**medical records technician school**	Broad match
**home health nurse narrative notes**	Broad match
**what is digital health**	Broad match
**telemedicine appointment booking system**	Broad match
**new patient medical form**	Broad match
**patient appointment scheduling system**	Broad match
**health software**	Exact match (close variant)
**aria healthcare management**	Broad match
**co immunity**	Broad match
**healthcare referral management system**	Broad match
**free medical office software**	Phrase match (close variant)
**digital health organizations**	Broad match
**care management platform**	Exact match (close variant)
**medical coding for beginners free**	Broad match
**computer programs used in healthcare**	Broad match
**health information management**	Phrase match (close variant)
**digital health projects**	Broad match
**medical necessity software**	Broad match

**gaps in care program**	Broad match
**what is an example of population health management**	Broad match
**healthcare information management**	Exact match (close variant)
**health information management technology salary**	Broad match
**sample healthcare survey questions**	Broad match
**volunteer for medical research**	Broad match
**population health staffing**	Broad match
**arc clinical research**	Broad match
**john hopkins research studies**	Broad match
**emr electronic medical records**	Exact match
**chronic care management codes**	Broad match
**emr companies**	Broad match
**medical problem list pdf**	Broad match
**average cost of epic emr**	Exact match
**best psychotherapy notes software**	Broad match
**healthcare it**	Broad match
**best patient management software**	Phrase match (close variant)
**epic electronic health record**	Broad match
**student health form**	Broad match
**health professional programs**	Broad match
**clinical care management**	Broad match
**medicare palliative care program**	Broad match
**what is stewardship in healthcare**	Broad match
**home health care programs**	Broad match
**wellstar cma program**	Broad match
**what is a app medical**	Broad match
**medical disclosure**	Broad match
**healthcare consultant**	Broad match
**healthcare management training programs**	Broad match
**ovid 19**	Broad match
**j&j booster study**	Broad match
**ssm my chart login**	Broad match

**how to check online medical**	Broad match
**new patient medical history questionnaire**	Phrase match
**what is epic software**	Exact match
**clinical training software**	Broad match
**medical technician online programs**	Broad match
**together study**	Broad match
**novavax fda approval**	Broad match
**personal health a population perspective**	Broad match
**management of care**	Broad match
**pfizer news**	Broad match
**what is health informatics**	Broad match
**hospital bed management**	Broad match
**endpoint for exchanging healthcare information**	Broad match
**johns hopkins clinical trials**	Broad match
**ehr project manager**	Phrase match (close variant)
**population healthcare management**	Broad match
**digital health id**	Phrase match (close variant)
**electronic charting system**	Broad match
**mychart northeast georgia health system**	Broad match
**hospital information system companies**	Broad match
**vaccine record pdf**	Broad match
**how to organize medical records**	Phrase match (close variant)
**myunity emr**	Phrase match (close variant)
**personal health records management**	Phrase match (close variant)
**what is hospital information system**	Broad match
**veracity emr**	Phrase match (close variant)
**medical records degree online**	Broad match
**epic patient list**	Broad match
**https c19study com**	Phrase match (close variant)
**cdc risk**	Broad match
**epic company**	Exact match
**health information portal**	Broad match

**emerging health information technology**	Broad match
**personal health records**	Broad match
**nephrology emr**	Broad match
**medical coding software**	Broad match
**e medical records**	Exact match
**health survey**	Broad match
**test results and electronic medical records**	Broad match
**insync ehr**	Broad match
**health history questionnaire**	Broad match
**emory healthcare patient blue portal**	Broad match
**medrecs com**	Broad match
**medical data abstraction**	Broad match
**electronic health care resources**	Exact match
**medical case study examples free**	Broad match
**myhealth chart**	Broad match
**e clinic software**	Broad match
**healthicity compliance**	Broad match
**health reporting system**	Broad match
**patient fusion patient portal**	Broad match
**free medical billing software programs**	Phrase match (close variant)
**baylor vaccine study**	Broad match
**hospital software systems**	Broad match
**epic training video**	Broad match
**what systems do hospitals use**	Broad match
**hr software for healthcare**	Broad match
**acupuncture clinic software**	Broad match
**benefit of electronic health record**	Exact match
**patient health record portal**	Phrase match (close variant)
**software healthcare**	Exact match (close variant)
**drawbacks of electronic health records**	Exact match
**trinity health mychart sign in**	Broad match
**epic medical records software**	Phrase match (close variant)

**population health services**	Broad match
**care management system**	Exact match (close variant)
**patient history forms**	Broad match
**patient registration systems**	Broad match
**clinical abstraction specialist**	Broad match
**medical records coder**	Phrase match (close variant)
**electronic health products**	Broad match
**ehr systems in healthcare**	Exact match (close variant)
**epic certification training schedule**	Broad match
**www medical records com**	Exact match
**patient portal application**	Broad match
**hospital software engineer**	Broad match
**employee medical questionnaire**	Broad match
**axxess home health software**	Phrase match (close variant)
**population health analytics companies**	Broad match
**practice management systems**	Broad match
**medical referral**	Broad match
**occupational health software**	Phrase match (close variant)
**top gym management software**	Broad match
**emr health care software**	Broad match
**electronic health record**	Exact match (close variant)
**mental health ehr systems**	Broad match
**healthcare epic mychart**	Broad match
**allscripts tutorial 2020**	Broad match
**population health articles**	Broad match
**medical assistance program online**	Broad match
**one health platform**	Broad match
**mychart leehealth**	Broad match
**full patient assessment**	Broad match
**pfizer vaccine trials**	Phrase match (close variant)
**how to manage a hospital**	Broad match
**medical charting systems**	Exact match

**digital health brands**	Broad match
**health service management**	Broad match
**research about pandemic**	Broad match
**printable health record template**	Broad match
**health record**	Broad match
**epic medical billing software**	Phrase match (close variant)
**centricity emr tutorial**	Broad match
**public health tb test**	Phrase match (close variant)
**free health assessment**	Broad match
**how hospitals work**	Broad match
**epic software**	Broad match
**electronic medical forms**	Broad match
**caresync**	Broad match
**epic system**	Broad match
**epic software training for nurses**	Broad match
**new patient registration**	Broad match
**electronic medical record**	Exact match (close variant)
**cancer patient assistance programs**	Broad match
**the medical record**	Exact match
**health data mining**	Broad match
**population health startups**	Broad match
**patient information sheet**	Broad match
**population health conferences**	Broad match
**modernizing medicine software**	Phrase match (close variant)
**online patient portal**	Broad match
**sign up my chart**	Broad match
**medical billing and coding online program**	Broad match
**north shore health system**	Broad match
**digital health platforms**	Exact match (close variant)
**ehr consulting companies**	Phrase match (close variant)
**patient information system**	Broad match
**how to document medical necessity**	Broad match

**personal health record template**	Phrase match (close variant)
**test results and electronic medical records**	Exact match
**pfizer study**	Broad match
**healthcare software**	Exact match (close variant)
**canopy healthcare software**	Broad match
**clinical decision support software fda guidance**	Broad match
**northwell health medical records**	Broad match
**medical iot**	Broad match
**free e prescribing software**	Broad match
**health electronic response data system**	Phrase match (close variant)
**athena charting**	Broad match
**learn epic software**	Exact match
**digital medical records**	Exact match (close variant)
**ehr certified application analyst iii**	Phrase match (close variant)
**himmss**	Broad match
**medical records spreadsheet**	Phrase match (close variant)
**hospital management ppt**	Broad match
**phresia**	Broad match
**population health solution**	Broad match
**epic anesthesia**	Broad match
**quest testing labs**	Broad match
**mybaptisthealth**	Broad match
**allscripts charting**	Broad match
**intergy ehr training**	Broad match
**list of all emr systems**	Broad match
**referral management**	Broad match
**hospital software programs**	Broad match
**patient form template**	Broad match
**search heath**	Broad match
**clinic quality**	Broad match
**livongo**	Broad match
**access my medical records online**	Broad match

**emr systems in healthcare**	Exact match
**https mychart stdavids com**	Broad match
**electric health records**	Exact match
**health care administration program**	Broad match
**cactus credentialing software**	Broad match
**athena healtj**	Broad match
**diabetes care management programs**	Broad match
**electronic patient record systems uk**	Broad match
**life care plan software**	Broad match
**care plan template**	Broad match
**diagnosis based screening**	Broad match
**remdesivir research trials**	Broad match
**free clinics for tb testing**	Broad match
**top 10 emr systems**	Exact match (close variant)
**free medical forms templates**	Broad match
**what is a cms in healthcare**	Broad match
**medical analyst**	Broad match
**meditech software**	Phrase match (close variant)
**healthcare population management**	Broad match
**hospital policies**	Broad match
**ppd test**	Broad match
**patient management software canada**	Phrase match (close variant)
